# Centering Indigenous knowledge in suicide prevention: a critical scoping review

**DOI:** 10.1186/s12889-022-14580-0

**Published:** 2022-12-19

**Authors:** Erynne Sjoblom, Winta Ghidei, Marya Leslie, Ashton James, Reagan Bartel, Sandra Campbell, Stephanie Montesanti

**Affiliations:** 1 School of Public Health, University of Alberta, 3-300 Edmonton Clinic Health Academy, 11405 - 87 Ave, Edmonton, AB T6G 1C9 Canada; 2 Métis Nation of Alberta, #100 Delia Gray Building, 11738 Kingsway Avenue NW, Edmonton, AB T5G 0X5 Canada; 3 Librarian, Health Sciences, University of Alberta, Edmonton, AB T6G 2R7 Canada; 4 School of Public Health, University of Alberta, 3-266 Edmonton Clinic Health Academy, 11405 - 87 Ave NW, Edmonton, AB T6G 1C9 Canada

**Keywords:** Indigenous populations, Suicide prevention, Community-based research, Culture, Scoping review, Indigenous knowledge

## Abstract

**Background:**

Indigenous peoples of Canada, United States, Australia, and New Zealand experience disproportionately high rates of suicide as a result of the collective and shared trauma experienced with colonization and ongoing marginalization. Dominant, Western approaches to suicide prevention—typically involving individual-level efforts for behavioural change via mental health professional intervention—by themselves have largely failed at addressing suicide in Indigenous populations, possibly due to cultural misalignment with Indigenous paradigms. Consequently, many Indigenous communities, organizations and governments have been undertaking more cultural and community-based approaches to suicide prevention. To provide a foundation for future research and inform prevention efforts in this context, this critical scoping review summarizes how Indigenous approaches have been integrated in suicide prevention initiatives targeting Indigenous populations.

**Methods:**

A systematic search guided by a community-based participatory research (CBPR) approach was conducted in twelve electronic bibliographic databases for academic literature and six databases for grey literature to identify relevant articles. the reference lists of articles that were selected via the search strategy were hand-searched in order to include any further articles that may have been missed. Articles were screened and assessed for eligibility. From eligible articles, data including authors, year of publication, type of publication, objectives of the study, country, target population, type of suicide prevention strategy, description of suicide prevention strategy, and main outcomes of the study were extracted. A thematic analysis approach guided by Métis knowledge and practices was also applied to synthesize and summarize the findings.

**Results:**

Fifty-six academic articles and 16 articles from the grey literature were examined. Four overarching and intersecting thematic areas emerged out of analysis of the academic and grey literature: (1) engaging culture and strengthening connectedness; (2) integrating Indigenous knowledge; (3) Indigenous self-determination; and (4) employing decolonial approaches.

**Conclusions:**

Findings demonstrate how centering Indigenous knowledge and approaches within suicide prevention positively contribute to suicide-related outcomes. Initiatives built upon comprehensive community engagement processes and which incorporate Indigenous culture, knowledge, and decolonizing methods have been shown to have substantial impact on suicide-related outcomes at the individual- and community-level. Indigenous approaches to suicide prevention are diverse, drawing on local culture, knowledge, need and priorities.

**Supplementary Information:**

The online version contains supplementary material available at 10.1186/s12889-022-14580-0.

## Background

Suicide is a pressing health concern that continues to disproportionately impact Indigenous populations around the globe. Indigenous peoples of Canada, United States, Australia, and New Zealand experience rates of suicide approximately two to three times higher than the general population of their respective countries [1–5]. At the individual-level, the primary risk factors for suicide are mental health disorders, traumatic/stressful life events, and substance abuse [6–8]. All of these risk factors occur at disproportionately high rates in Indigenous populations as a result of the collective and shared trauma experienced with colonization and contemporary experiences of oppression and social exclusion including dispossession and disconnection from the land, loss of language and culture, grief and loss, and racism [9–14].

The rate of suicide among First Nations, Métis, and Inuit peoples in Canada is at least two times that of Canada’s general population [1]. In the United States, the rate of suicide among the American Indian/Alaskan Native population is approximately 3.5 times higher than those among racial/ethnic groups with the lowest rates and about 1.7 times higher than the overall US rate [2, 3]. In Australia, suicide rates for Indigenous people ranged from 1.4 to 2.4 times that of non-Indigenous Australians in 2020 [4]. In New Zealand, suicide among Māori from 2010 to 2012 was 1.8 times greater than among non-Māori, with particularly high rates among Māori men [5].

Nevertheless, national-level data can obscure considerable variability in suicide rates and patterns between communities and wider regions [1, 4, 15–17]. While there is evidence demonstrating the connection to broader protective factors like employment status, educational attainment, and social support networks at the individual-level [6] this paper focuses on the community-level risk and protective factors for Indigenous populations. Research has identified a number of community-level factors that have been demonstrated to create resiliency to suicide in Indigenous populations, and can explain much of the observed variability in suicide rates across different communities. These protective factors include advances towards self-determination, efforts to secure Indigenous title to traditional lands, and activities that promote and protect Indigenous culture and language [15, 16, 18]. Research has also demonstrated that integrating Indigenous knowledge into mental wellness promotion, prevention and intervention initiatives has been associated with positive outcomes, including in suicide rates [19–21]. Calls continue to grow for upholding Indigenous peoples’ right to self-determination in defining effective and culturally-grounded means to address health and wellness needs in their respective communities [22, 23]. Moreover, a growing body of research contends that standard suicide prevention programs—primarily rooted in Western individual-level efforts for behavioural change via mental health professional intervention—are culturally misaligned with Indigenous paradigms of health, mental wellbeing, and relationality [24, 25]. For these reasons, many Indigenous communities, organizations and governments have been moving away from initiatives designed for the general population and moving towards more cultural and community-based approaches for mental health promotion [26].

Despite these advancements in knowledge and understanding of the unique factors impacting Indigenous peoples’ risk and resilience for suicide, suicide prevention initiatives continue to fall short of meeting the needs of Indigenous peoples who are at a higher risk of adverse mental health outcomes and experience limited access to appropriate care and resources. Overall, there is a notable gap in comprehensive community-based, culturally safe suicide prevention resources for Indigenous communities. In recent years, many suicide prevention programs targeting Indigenous populations and incorporating Indigenous approaches have been developed; however, a thorough review of these initiatives has yet to be conducted. While a number of reviews of Indigenous suicide prevention initiatives have been conducted, they have focused on specific Indigenous groups (e.g., Indigenous youth, Inuit, or American Indian/ Alaska Native populations), particular programs employed in Indigenous populations (e.g. Adolescent Suicide Prevention Project), or focused on specific types of evidence (e.g. case files or evaluated programs only). To the authors’ knowledge, no reviews to-date have broadly examined suicide prevention efforts employed in Indigenous populations nor explicitly the contribution of Indigenous knowledge. A comprehensive exploration of how Indigenous approaches have been incorporated into suicide prevention efforts to-date could be instrumental in informing and supporting further development of Indigenous-driven suicide prevention. Consequently, this review explores how Indigenous knowledge and approaches have been incorporated in suicide prevention for Indigenous populations.

A critical scoping review was conducted to conceptualize, map and identify gaps in the literature and to assess if Indigenous knowledge was the guiding principle in developing these programs and interventions. Scoping reviews aim to map ‘the key concepts underpinning a research area and the main sources and types of evidence available [27, 28]. As such they differ from systematic reviews in focusing on broader topics and a range of study designs with little emphasis on quality; nor are they designed to perform detailed assessments or synthesis of findings [29]. Our critical scoping review aligned with the processes and objectives of a scoping review as recommended by Arksey and O’Malley [29] and Levac, Colquhoun, and O’Brien [30]. Additionally, we applied a two-step process to better align with ethical standards of research involving Indigenous peoples, and to enable Indigenous knowledge to inform the evidence appraisal and interpretation: 1) Indigenous and non-Indigenous co-authors synthesized the evidence; and 2) input was sought from a reference group of Indigenous community leaders with expertise in Indigenous knowledge systems.

## Methods

### Community engagement

This review utilized a community-based participatory research (CBPR) approach. The need for a critical scoping review arose out of an existing project to develop a Métis suicide knowledge awareness training program through extensive community engagement sessions led by the Métis Nation of Alberta (MNA). At the start, the MNA approached the academically-situated members of the research team (SM, WG) to work together to pursue research funding and work in partnership to develop a community-driven, culturally-grounded suicide prevention program. It was determined that this scoping review would provide a thorough knowledge base for how Indigenous approaches have been integrated into suicide prevention targeting Indigenous populations and inform the development of a Métis suicide knowledge awareness intervention. Consequently, the methods, emergent themes and subthemes, analysis of outcomes, and final manuscript were all co-developed between the MNA team members and University-situated research team members.

### Information sources and search strategy

A scoping review of both academic and grey literature was employed to examine relevant evidence on how Indigenous knowledge has been incorporated in suicide prevention initiatives. We felt it key to include an online grey literature search in recognition that many Indigenous communities may have implemented suicide prevention efforts that might not always be formalized in the academic literature. We first developed a list of search terms in consultation with a research librarian and used combinations of the following search terms and their synonyms: (suicid* or "self harm"); (communit* or family or families or caregiver* or gatekeeper*); (awareness or prevent* or know* or educat* or train or trained or training); (indigenous people/ or alaska native/ or american indian/ or canadian aboriginal/ or first nation/ or indigenous australian). Search strategies were designed to be suitable to the specific features of each database (Additional file 1: Appendix A). The following databases were searched: Prospero, Wiley Cochrane Library, Ovid Embase, Ovid Medline, Ovid Global Health, Ovid PsycInfo, EBSCO CINAHL, EBSCO Socindex, ProQuest Dissertations and Theses Global, and SCOPUS. All of the databases were searched from inception to November 2021. The search strategy included both text words and controlled vocabulary (eg: MeSH, EMTREE, etc.) for the concepts “Indigenous people” and “suicide prevention” and “community/caregiver awareness.” In addition, the reference lists of articles that were selected via the search strategy were hand-searched in order to include any further articles that may have been missed.

We also systematically searched for grey literature (Indigenous texts, songs, videos, artform, reports, etc.) in the following online databases and resource hubs: University of Alberta-Native Studies Databases, National Collaborating Center for Indigenous Health, International Journal for Indigenous Health, Health Canada’s National Aboriginal Youth Suicide Prevention Strategy (NAYSPS), Center for Suicide Prevention, and the Thunderbird Partnership Foundation. Additionally, we ran a customized Google Scholar search on the terms “Suicide Prevention” AND (Indigenous OR Aborigin OR First Nation OR Inuit OR Métis OR Native) and examined the first 20 pages of the returned results.

### Selection process

The articles resulting from the search were screened for relevance and subjected to a critical appraisal process by two reviewers (WG, ML). Only English language publications were considered. Relevance was established by the research team first by reviewing the title and abstracts of the identified literature against the review objectives. Specifically, articles were included if (1) the papers discussed programs and/or initiatives that aimed to prevent suicide in Indigenous populations and (2) the target population were Indigenous populations of Canada, United States, Australia and New Zealand. Canada, the United States, Australia, and New Zealand are commonly seen as natural comparators in terms of Indigenous well-being. These jurisdictions consistently rank highly on the United Nations Development Programme’s Human Development Index (HDI), yet all have minority Indigenous populations with much poorer health and social conditions than their non-Indigenous population [31], including a disproportionate burden of suicide. Moreover, Indigenous peoples in these countries share similar experiences as subjects of British colonialism, including comparable colonial histories, laws, policies, and political structures. Such similarities include processes of treaty making (except Australia), policies aimed at assimilation, paternal protectionism, dislocation from the land to make way for settlers, and loss of culture [32, 33]. These countries have also undertaken comparable efforts towards reconciliation with Indigenous peoples in recent years [34]. Where relevance could not be determined from the article title or abstract alone, a review of the full text was conducted. There was no limitation based on study design and source type (academic and grey literature were included). Any literature that did not fit into the above criteria, or that addressed other mental health prevention programs (other than suicide) was excluded. Grey literature articles were screened first on the basis of relevancy of their title to the research objectives of this scoping review, with further review of the full document led to the exclusion of any additional irrelevant grey literature articles.

### Article and data management

The following information was extracted from academic literature into a standard extraction form: authors, year of publication, type of publication, objectives of the study, country, target population, type of suicide prevention strategy, description of suicide prevention strategy, and main outcomes of the study. We also took detailed notes on whether suicide knowledge was defined from the perspective of the local Indigenous communities and the level of involvement of local Indigenous communities, including in project development and paper co-authorship. Findings from grey literature were summarized by the research team with consideration of the following questions: What does suicide and suicide prevention mean from an Indigenous perspective? How has Indigenous knowledge been incorporated in suicide prevention?

### Thematic analysis

We adopted a thematic analysis approach guided by Métis knowledge and practices to synthesizing and summarizing the findings. Our research team is composed of Indigenous (Métis) and non-Indigenous members. First, two researchers (WG and ML) worked together to read all the articles, annotate them, and identify broad thematic categories. Next, additional researchers (SM, AJ, and RB) discussed each theme and subtheme until they reached consensus in a team meeting. After this team meeting, WG and ML compared and contrasted the various findings to identify recurrent and unique themes. Findings from both academic and grey sources were merged and appropriate themes were applied. All team members reviewed themes, discussed disagreements between them and reached a consensus. Each term, phrase and/or meaning used to contract the categories and themes were confirmed with Métis knowledge holders. The academic literature and grey literature articles were then thematically analyzed by WG and ML simultaneously who used NVivo™ to apply codes to the articles on a consensus basis. This also involved examination of articles to document similarities for the purpose of identifying common themes across suicide prevention, while also detailing their distinctions and differences.

## Results

**Fig. 1:**
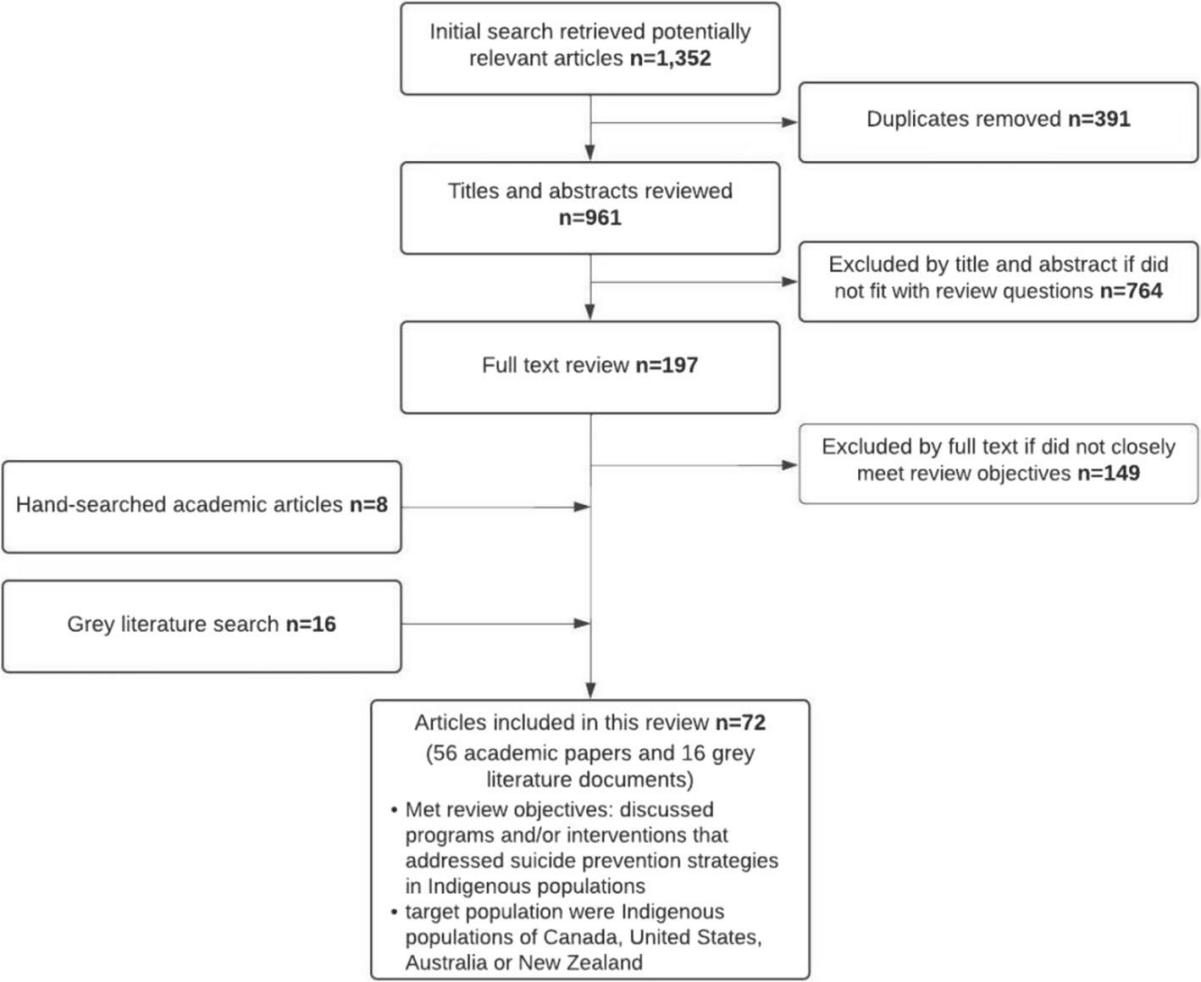
Flow chart describing included and excluded articles

The review process resulted in the collection of 1,352 academic papers and grey literature documents, with 391 articles ultimately considered after duplicates were removed. Of these, 961 were excluded as, despite appearing in the search, upon interrogation of the article title and abstract, it was determined that the content of the article fell outside of the scope of the review objectives. 197 articles necessitated full text review as inclusion could not be determined from the title and abstract alone. Of these, 149 further papers were excluded because they did not align with our review objectives nor did not meet inclusion criteria. 8 articles were identified via a hand-search of the reference lists of relevant reviews. Sixteen articles from the grey literature were ultimately included as well. Thus, our final search process resulted in 72 articles—56 of which were academic papers and 16 grey literature documents—that were included for extraction (Fig. 1). The characteristics of the articles along with the overarching themes identified through thematic analysis are summarized in the following section.

### Article characteristics

This section presents an overview of the suicide prevention strategies targeting Indigenous populations examined in this review, namely describing the Indigenous communities involved, target demographics, and the types of strategies employed. Articles from the academic literature were primarily peer-reviewed outcomes of primary research activities involving the above types of suicide prevention strategies along with several different types of academic reviews. Articles obtained from the grey literature were diverse and included for suicide prevention guides, strategies, toolkits, outcomes of community engagement, and more typically authored by Indigenous communities and organizations.

### Indigenous population and sample

Thirteen articles focused on Indigenous Australians; thirty-two on Indigenous peoples living in the United States (US) (specifically fourteen with Alaska Natives, four Alaska Natives and/or American Indians; eleven American Indians/Native Americans, and three Native Hawaiians); nine on Indigenous peoples in Canada; one on Indigenous peoples in North America; and one on Indigenous peoples in Canada, the US, Australia and New Zealand. No articles involved the Māori of New Zealand. The sample populations reported by studies included Indigenous youth, general community members, or specific subpopulations (such as Indigenous prisoners, students, or males). Notably, no papers incorporated considerations for lesbian, gay, bisexual, transgender, queer, and two-spirited (LGBTQ2 +) or gender diverse Indigenous persons.

### Types of articles

The main prevention strategies employed in articles examined in this scoping review comprised: culture as treatment; community prevention activities; gatekeeper training; and education/awareness initiatives. These articles presented peer-reviewed outcomes of primary research activities involving the above types of suicide prevention strategies (39 articles). Methods employed in the primary research articles included pre/post studies (8); randomized (4) and non-randomized (4) control trials; retrospective study design (1); qualitative methods including focus groups, workshops, interviews, and more (15); and mixed-methods approaches (7). The remaining articles were papers describing intervention development and implementation (11 articles) and different types of academic reviews (6 articles). See Additional file 2: Appendix B for a table detailing key information extracted from academic literature. Articles obtained from the grey literature concerned: guides, strategies, toolkits for suicide prevention in Indigenous populations, outcomes of community engagement on suicide prevention; curriculum documents; brochures, magazine or news articles describing suicide prevention projects; and literature reviews (see Additional file 3: Appendix C).

**Fig. 2:**
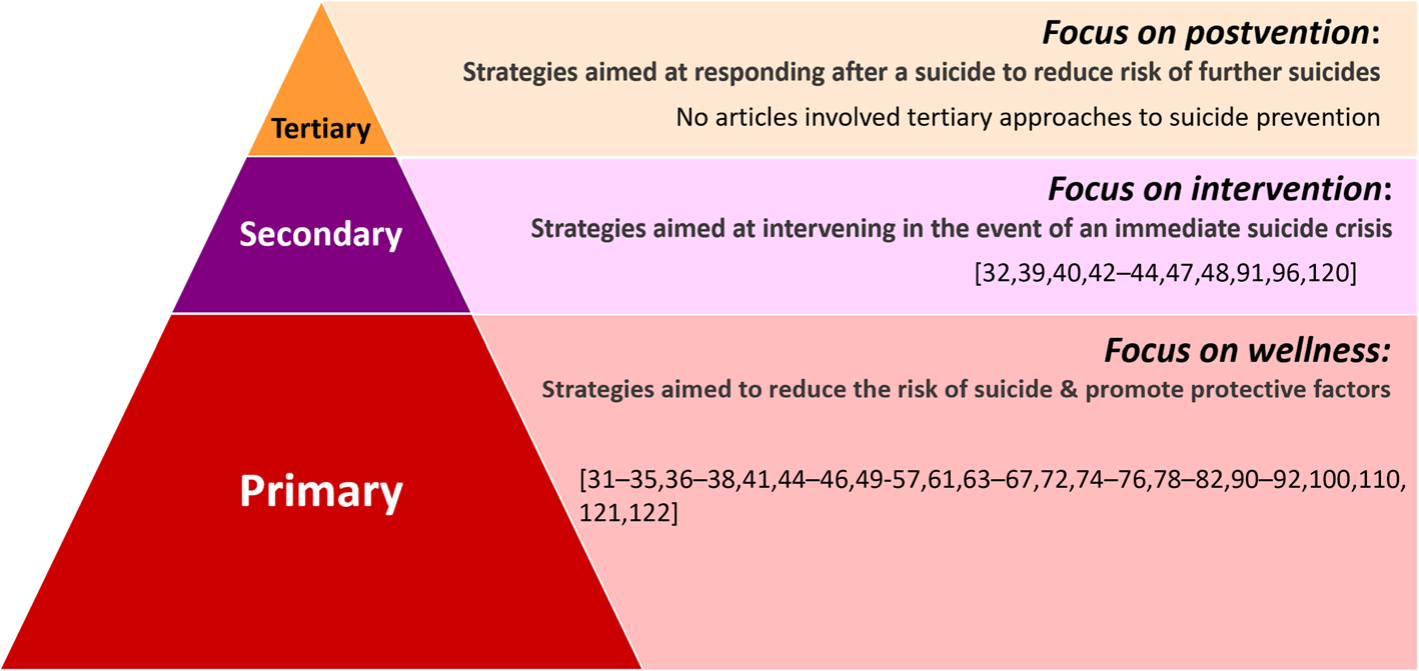
Articles by level of suicide prevention [35]

Culture as treatment specifically involved engaging Indigenous culture to mitigate suicide risk or “treating” suicidality among individuals, usually on a one-on-one basis. Community prevention initiatives typically involved empowerment programs, multi-level approaches, broader resiliency strategies targeting Indigenous groups and communities at high risk of suicide, or community-based participatory research to inform program development. Gatekeeper training strategies featured prominently. Gatekeeper training involved teaching specific groups of people in the community how to identify and support individuals at high risk of suicide. Education/awareness initiatives involved activities that explicitly aimed to improve suicide knowledge, attitudes, and/or awareness to develop knowledge/skills that are known to be protective against suicide via, for example, school-based programs for youth, multi-media education sessions to interested community members, or culturally-tailored life skills training for youth. 25 articles involved community prevention, 13 concerned educational/awareness initiatives, 7 featured gatekeeper trainings, 3 involved culture as treatment, 1 featured both community prevention and gatekeeper training components, and 1 involved community prevention, gatekeeper, and education/awareness approaches. The remaining 6 were scoping or systematic reviews of Indigenous suicide prevention projects/programs. Community prevention and education/awareness initiatives involved primary prevention that address upstream root causes and aim to prevent suicide ideation or attempts from even happening by reducing risk and promoting protective factors. Gatekeeper training and culture as treatment involved secondary prevention which endeavour to provide support to persons at immediate risk for suicide/self-harm. No articles involved tertiary prevention efforts which might involve postvention to reduce the risk of further suicides or clusters. Levels of suicide prevention and corresponding articles are detailed in Fig. 2.

### Themes

The findings are presented through four overarching and intertwining thematic areas that emerged out of analysis of the academic and grey literature. These thematic areas focus on (1) engaging culture and strengthening connectedness; (2) integrating Indigenous knowledge; (3) Indigenous self-determination; and (4) employing decolonial approaches. We also highlight components of strategies that exemplify each theme. Table 1 summarizes themes, subthemes and corresponding articles.

**Table 1:** Themes, subthemes, and corresponding articles

Theme	Subtheme	Articles
Engaging culture & strengthening connectedness	Culture as treatment	[36–44]
	Engaging cultural connections	[36, 41, 45–59]
	Intergenerational relationships (youth & Elders)	[38, 49, 59–70]
	Strengthening connectedness in families & communities	[62, 69, 71–73]
	Cultural continuity	[65, 71, 74, 75]
	Cultural intervention vs. Culturally-appropriate, -sensitive, or -safe intervention	[38, 39, 50, 53, 55, 68, 69, 73, 76–78]
Integrating Indigenous knowledge	Indigenous understandings of suicide defined by authors	[36, 41, 43, 44, 46–48, 50, 51, 56–58, 61, 62, 68, 70, 79–89]
	Indigenous understandings of suicide defined via community engagement	[45, 53–55, 60, 67, 69, 73, 76, 90–97]
Indigenous self-determination	Community-based participatory research	[38, 39, 44, 50, 53, 60, 68, 69, 73, 76]
	Other community-engaged approach	[37, 40–42, 44, 46–49, 51, 52, 54, 56–59, 61, 62, 67, 78–80, 82, 84–88, 90–92, 94, 95, 98]
	Indigenous ownership	[45, 57, 60, 65, 83, 97, 99]
Employing decolonial approaches	Cautioning against pan-Indigenous approaches	[56, 67, 71, 90]
	Integrating contextual considerations	[48, 55, 68, 76, 93, 98]
	Other decolonial approaches	[44, 45, 48, 49, 54, 59, 68, 83, 91, 92, 97, 98]

### Engaging culture and strengthening connectedness

Engaging culture and strengthening connectedness to prevent suicide emerged as an important theme across the articles examined in this critical scoping review. All articles highlighted connection to culture as a crucial component to meaningful and effective suicide prevention in Indigenous populations. Within this theme, we focused on analyzing the ways in which culture and efforts to strengthen connectedness are integrated into suicide prevention content and the resulting impacts.

Generally, engaging culture took on several different forms. First, some initiatives were built around the notion of “culture as intervention” or “culture as treatment,” where engaging Indigenous culture was seen as an important means for mitigating suicide risk or “treating” suicidality among individuals. Culture as intervention or treatment could be the main strategy, or a component of a broader strategy, and took on several forms including resilience retreats/culture camps, cultural teachings/values, ceremony, sharing circles, storytelling, creative arts, narrative approaches to psychotherapy, art therapy, other locally-relevant healing/coping strategies and more [36–44].

In these initiatives, individual-level effects on suicide risk via cultural connections included increases in the number of protective behaviours which authors argue were fostered by culture-specific beliefs and experiences that make life enjoyable, worthwhile, and meaningful [38]. Individual-level impacts among participants included reductions in distress, bolstering of protective factors, and reduction in suicide/self-harm behaviours [38, 40, 44]. Activities that involved local Indigenous culture as suicide prevention were observed to have a measurable impact on suicide-related outcomes such as increased positive mood, feelings of belongingness, and perceived coping of participants, even in programs where the specific topic of suicide was not breached [36, 37].

Second, engaging cultural connections arose as an important means to create new or adapt existing suicide prevention strategies to increase effectiveness and appropriateness in Indigenous contexts. Initiatives often took the form of gatekeeper trainings or educational/awareness initiatives and were typically created or adapted from the ground up either in partnership with or, less frequently, under the leadership of the respective Indigenous community [48–51]. Cultural inclusion in the design of programs or adaptation involved a multitude of factors including: acknowledgement of the impacts of colonization and ongoing marginalization on suicide in Indigenous contexts, integration of Indigenous pedagogies (i.e. team-teaching, land-based learning, experiential/hands-on activities, etc.), emphasis on the holistic aspects of wellbeing, focus on strengths-based approaches, incorporating art, and inclusion of Indigenous languages and cultural values [36, 45–48, 51–55].

Authors underlined the positive impacts of integrating local Indigenous culture at both the individual- and community-level. For instance, individual-level impacts for gatekeeper trainings included improved attitudes toward suicide, increases in participants’ knowledge and confidence in how to identify individuals at-risk of suicide, increases in intended and actual assisting behaviours, and significant improvements in understanding the links between cultural strengths, social and emotional wellbeing and suicide prevention [47, 48, 51, 52]. Moreover, participants in culturally-grounded suicide education and awareness initiatives were shown to have less suicidal ideation and “negative thinking”, expressed fewer feelings of hopelessness, could come to terms with the ‘cycle of grief,’ demonstrated reduced stigma towards suicide and increased willingness to seek help, and had an increase in psychological service utilization [49, 56–58]. Participants in community suicide prevention programs which integrated culture had significant increases in positive mood, feelings of belongingness, perceived coping, reasons for living, and overall resiliency [36, 38, 41].

Strengthening connectedness was consistently identified by articles as an important element for effective suicide prevention in Indigenous populations. We included it along with the theme of engaging culture as it was typically discussed as a key Indigenous cultural value which contrasted conventional Western approaches to suicide prevention. While there is much diversity in Indigenous ways of being and knowing, the ontologies of interconnectedness and relationality are shared across many of the Indigenous populations involved in articles reviewed. Strengthening connectedness comprised emphasis on encouraging intergenerational relationships, particularly between youth and Elders, strengthening connectedness within families and whole communities, and bolstering cultural continuity.

Fostering relationships between youth and Elders was a frequent community-identified means of prevention to support protective factors and promote healing among youth via opportunities to learn cultural teachings, language, and connect with the land and spirit with Elders who are the holders of a community’s traditional knowledge. Three strategies featured approaches that brought together youth and Elders as part of suicide prevention, which were noted to have implications for protective factors against suicide among youth such as strengthening youth reasons for living and combating “discontinuity” [38, 60, 61]. Furthermore, a participatory action research project that sought to explore community-identified risk factors as well as strategies to strengthen protective factors found connection between youth and Elders to be an important community-level strategy to suicide prevention [62].

This importance of bringing together youth and Elders was also echoed across the grey literature, typically as outcomes of community engagement on suicide prevention. Reports emphasized how Indigenous culture, knowledge, and language—which impart protection against suicide—are transferred from Elders to youth and suicide prevention thus needs to foster these relationships [63–66]. This sentiment is embodied in a quote from an Elder from an Australian Indigenous community experiencing high rates of youth suicide and self-harm: “The only way to stop suicide is to fulfill our cultural obligation to teach our young ….strength of character through strength of culture” [64]. Other suicide prevention initiatives did not necessarily bring together youth and Elders as an intervention component, but still created opportunities for connecting them as part of community engagement processes [44, 59, 66–70, 79].

Notably, academic and grey literature articles also spoke to the importance of strengthening connectedness within families and across community as part of suicide prevention [62, 69, 71–73]. For families, this could include restoring and strengthening connections within and between families through shared activities (especially cultural or spiritual activities); offering life skills programs; and providing access to education and/or training [62, 71]. For communities, fostering connections might involve a focus on youth (i.e., drop-in centres, camps, connect to Elders, health promotion and education sessions, parenting programs, restore sporting competitions); restoring and strengthening a sense of community through shared activities (i.e., community events, fun days, competitions, projects); upholding self-determination; men’s and women’s groups; and providing access to employment, education, housing and transport [62, 72].

Several articles noted the importance of efforts to bolster cultural continuity as part of suicide prevention. Authors stressed how strengthening “cultural continuity,” or the degree to which a community participates in actions symbolic of their sense of community as a cultural group, has positive implications for mental wellness, resilience, and thus suicide in Indigenous contexts [65, 71, 74, 75]. Many cite the research of Chandler and Lalonde [15, 16] to underline that a community’s effort to preserve the continuity of their collective culture can impact continuity at the individual-level and act as a hedge against suicide by facilitating individuals’ endurance through life’s routine hardships and build a connection to a sense of self and identity.

Despite the expressed importance of including cultural continuity in suicide prevention, no initiatives involved explicit efforts to support continuity of collective culture at the community-level to impact suicide. When culture was integrated into suicide prevention, it was primarily done so to impact the wellbeing, knowledge and/or behaviours of individuals, not the community as a whole. This was also reflected in the outcome measures captured in program evaluations.

A final subtheme around the definition of cultural intervention emerged from the literature reviewed in this scoping study. Many articles made the distinction between cultural intervention and culturally appropriate or culturally safe intervention. In the former, Indigenous culture is both a central focus of the intervention activities and underlies the theory guiding the intervention. In this sense, a cultural intervention is more likely to be transformative; underpinned by Indigenous ontologies, epistemologies, and/or worldviews; incorporate Indigenous notions of suicide; and be rooted in community defined and prioritized health issues [38, 39, 68, 69, 73].

Culturally-appropriate, -sensitive, -tailored, or -safe interventions, on the other hand, may incorporate Indigenous cultural activities, teachings, language and more but can still be dominated by and reproduce conventional Western/colonial understandings of mental wellness and perpetuate colonial power dynamics [38, 39, 55, 69, 73, 76–78]. As one author notes, the focus on culture by outsiders in health intervention has “too often been a shallow or surface translation describing more macro-level, formulaic, and ahistorical aspects of [Indigenous] life.” [53]. Cultural adaptations of conventional suicide prevention strategies may be more susceptible to reliance on the underlying Western/colonial assumptions of the original intervention and typically involve modifying “non-active” treatment components of the intervention for cultural acceptability such as language or style of the intervention, who delivers it, or the treatment setting [50]. Many adaptations also place importance on finding a balance between meeting community/cultural needs and preserving fidelity/standardization [54, 56, 71, 80].

### Integrating Indigenous knowledge

Integrating Indigenous knowledge into suicide prevention arose as a prominent theme across articles included in this scoping review. In this section, we specifically focus on how Indigenous knowledge impacts the conceptualizing of the issue of suicide and subsequently shapes how programs are designed and implemented. The majority of articles attempted to define the issue of suicide from an Indigenous perspective as a foundational step to developing an appropriately community-driven initiative. This was achieved through two methods: via author-driven definitions or via definitions acquired through community engagement processes. In cases where definitions were proposed by authors, suicide was commonly defined in connection to assumed Indigenous notions of wellness in general. For example, one study’s authors characterized suicide in alignment with Indigenous perspectives which “[focus] more on understanding and addressing what is going on around the individual than addressing what is going on inside” [76]. Focusing on what is going on around the individual meant consideration of the “complex socio-cultural, political, biological and psychological phenomenon that needs to be understood in the context of colonization, loss of land and culture, transgenerational trauma, grief and loss, and racism and discrimination” [46].

Some strategies informed by this notion employed multi-level approaches (community-wide events, policy efforts, educational programs for youth, and traditional ceremonies) that involved multiple sectors of the community simultaneously (individuals, families, wider community) [38, 69, 73, 76, 90]. Others took aim at intervening on one or more of these broader determinants like, for example, seeking to support knowledge transfer via intergenerational relationships or just generally integrating Indigenous culture into curriculum content [60]. Other initiatives incorporated locally-relevant content, information about Indigenous culture or colonization and ongoing marginalization and how they contribute to the issue of suicide in Indigenous contexts [46, 49, 81].

On the other hand, articles that sought community-based understandings of suicide via engagement processes tended to emphasize a focus on strengths and resilience in opposition to the typical focus on deficits and problems [45, 53–55, 60, 67, 69, 73, 76, 90–97]. This is illustrated in a statement from an Elder who co-led the development of a youth resiliency project and co-authored the resulting paper: “Why do we talk about suicide all the time!? Let’s talk about love!” [45]. Thus, strategies informed from Indigenous notions of suicide intentionally shifted from a focus on deficit—which is more typical of conventional Western suicide prevention—to a focus on life promotion with attention to existing community strengths and assets, while upholding community control and sovereignty and supporting local empowerment [44, 68, 76, 90, 91, 93]. This connects with the subsequent section in which we discuss the theme of Indigenous self-determination in suicide prevention.

### Indigenous self-determination

Self-determination in suicide prevention was frequently identified as an essential requirement for success. The majority of articles employed some means to ensure some level of self-determination was achieved via community engagement in the design and/or implementation of suicide prevention. This was commonly achieved by employing community-based participatory research (CBPR) or participatory action research approaches. CBPR was utilized “to address power differentials through shared learning ….[it] is a move toward reconciliation, reciprocity, and production of culturally relevant prevention measures” [68]. Community engagement was seen by authors as a key requirement for success, as it increases community relevance, appropriateness, and in particular, ownership of suicide prevention [38, 39, 45, 57, 68, 76, 82, 93, 97, 99, 100].

The emergent subtheme of ownership referred not to the legal sense of the word (i.e., the right to possess and control the initiative) [100] but more to the community stepping up to take on responsibility in executing an initiative while being invested in seeing out its success [45, 57, 65, 83, 97, 99]. In this sense, community ownership in a strategy has implications for acceptability, uptake, participation, and dedication to investing necessary resources. Another reason why community ownership was expressed as crucial to suicide prevention success is because it is correlated with longevity and sustainability [45, 57, 83].

Indigenous authorship also emerged as a notable subtheme of Indigenous self-determination in suicide prevention. Many of the articles reviewed here highlighted that they were co-authored by members of the specific target Indigenous communities or members of broader Indigenous communities [36, 39, 41, 43–47, 50, 51, 53, 56, 60, 67–69, 80, 84, 94, 97]. Indigenous authorship was important to ensure materials represent content as intended by community stakeholders, particularly when it comes to native language expertise [60].

### Employing decolonial approaches

Lastly, employing decolonial approaches in suicide prevention creation and implementation was a prominent theme in the literature. Decoloniality—or the creation of “locally-governed, community-based, and culturally-responsive systems of care” [55]—has been touched upon in many of the themes already discussed above (i.e., incorporating Indigenous knowledge and culture, upholding self-determination, community-engaged approaches). In this section, we hence focus on other attributes of decolonial approaches highlighted in the literature, namely avoidance of pan-Indigenous approaches, integrating contextual considerations, and more generally, approaches that might diverge from features of conventional Western suicide prevention.

Use of pan-Indigenous approaches was cautioned in the literature and authors advised against application of Indigenous-driven suicide prevention in contexts that they were not designed for [56, 67, 71, 90]. This is because pan-Indigenous strategies may not be reflective of diverse cultural practices, values, sociohistorical context, and geographic considerations unique to each group. Authors stress that programs need to be adaptable to the local community context. This is especially pertinent in the issue of suicide, which can vary significantly from community to community [56, 68].

In line with this, integrating contextual considerations into suicide prevention was also expressed as an important decolonial component. This subtheme was most prominent in recommendations for suicide prevention in Indigenous populations outlined in the grey literature. Contextual considerations included gaining the knowledge of a community’s unique risk and protective factors, incorporating local examples where possible, respect for and adherence to local protocol, involvement of local experts/Elders, developing culture-centered understanding of suicide, assessing community readiness, building and maintaining relationships, contemplating the impact of recent incidents of suicide, considering existing level of trauma and unresolved grief, and incorporating healing components [48, 55, 68, 76, 93, 98].

Other decolonial approaches detailed in the literature specifically took aim at breaking down some of the features common in conventional, Western programs that tend to persist in Indigenous programs (especially cultural adaptions) despite communities finding them unhelpful. This includes allowing for flexibility, especially in contrast to rigid, standardized procedures employed by some interventions like, for example, in Applied Suicide Intervention Skills Training (ASIST). Flexibility could include encouraging use of Indigenous language, carrying out sessions in outdoor settings, allowing for adjusting of required time commitment, creating space for ceremony/spirituality, and more [54, 59, 68, 79, 82, 92].

Articles also discussed avoidance of clinical language or jargon which can create barriers to understanding content. Finally, articles discussed efforts to break down power imbalances between Western and Indigenous approaches by, for example, focusing on local empowerment and capacity by training Indigenous facilitators, involving locally-recognized experts/leaders/healers, and employing Indigenous ways of learning versus a focus on employing clinical experts or utilizing the typical didactic educational models [44, 48, 54, 79, 91, 98]. According to authors, breaking down power imbalances could also involve creating a practice of reflexivity as part of the strategy, where researchers and developers actively reflect on their relationships, position, and privilege and how they are fulfilling their obligations to community [45, 54, 82, 91, 97].

## Discussion

This scoping review set out to identify and describe what is known about the types of Indigenous approaches to suicide prevention that are employed with Indigenous populations and their implications for program outcomes. All articles, from both the academic and grey literature, were from Canada, the United States, or Australia. Notably, no suicide prevention initiatives from New Zealand were identified in our search strategy. We hypothesize that this may be because a higher rate of suicide among Māori in comparison to non-Māori is a relatively newer phenomenon and no public-facing reports on prevention strategies have been published yet [101]. There was also a notable absence of inclusion of considerations for Indigenous sexual minorities and gender diverse persons in suicide prevention. While the suicide rates among these groups is not well-known, two-spirited, queer, gay, lesbian, bisexual, or transgendered Indigenous persons experience suicide-related risk factors at a much higher rate than cis-gendered, heterosexual Indigenous persons [102]. Indigenous sexual minorities and gender diverse persons’ experiences around suicide, risk and protective factors may also be unique—including compounded effects of discrimination, arguably necessitating special consideration in suicide prevention [103].

Another noteworthy gap was the absence of articles concerning Indigenous approaches to tertiary suicide prevention, namely suicide and crisis response and postvention. Conventional crisis response and acquiring care via the medical system for a suicide attempt or self-harm may be present problems for Indigenous peoples as these systems may be culturally unsafe and possibly less helpful than they could be. In order to respond to a lack of accessible and culturally safe crisis and suicide response services, many First Nations in Canada are establishing their own mobile crisis response teams (e.g., Manitoba Keewatinowi Okimakanak, n.d.; Southern Chiefs Organization, 2022; Thunderbird Partnership Foundation, 2018 [104–106]). The activities of these teams commonly have culturally inclusive elements, but still follow mostly Western crisis response models. We are unaware of any Indigenous approaches to suicide postvention; however, given the issues many Indigenous communities face following the suicide of a fellow community member including trauma, grief and loss, and possibilities of suicide clusters [107], further exploration is warranted.

The grey literature articles included toolkits, guides, information resources, and strategies to support Indigenous communities and/or organizations in developing and implementing suicide prevention programs and increasing suicide knowledge and awareness. Both academic and grey literature articles emphasized the role of culture, community connectedness and Indigenous knowledge in the prevention of suicide. Whereas the grey literature articles provided practical “how to” resources, information and guidance for delivering suicide prevention programs, the academic literature provided knowledge of “what works” by reviewing the outcomes of different suicide prevention strategies and interventions in Indigenous communities, including effective approaches to community engagement, and identifying key components of culturally-appropriate, -sensitive, -tailored, or -safe interventions.

Outcomes of this critical scoping review align with much of the work being done within the context of the broader field of Indigenous mental wellness promotion and Indigenous health research in general, which is increasingly looking to approaches that are decolonizing; founded on community engagement and self-determination; and inclusive of Indigenous culture, language and knowledge [26, 108, 109]. Moreover, in Indigenous mental wellness promotion, there is also an increasing focus on intervening beyond the individual to the family- and community-level, with an emphasis on fostering strength and resiliency [110, 111]. Family and community are important resources for developing a sense of belonging, connectedness, meaning, and identity, all of which are well-established protective factors for overall mental wellness and suicide in Indigenous populations [112]. It is within the family and the community where Indigenous culture, knowledge and language is transmitted, positive cultural identity forms, and where one can seek out support in a crisis [112]. However, in many Indigenous contexts, family and community environments have been severely damaged with colonization [13, 113]. Hence why community engagement outcomes consistently point to a need to move beyond the individual to support family and community strengths [53]. This need was echoed in articles examined in this review; however, we found that few suicide prevention initiatives employed broader family and community approaches.

Many of the suicide prevention programs identified as “community prevention” still primarily focused on protective and risk factors among individual participants and not on broader familial and community elements. As noted by Cox et al., [62] family-level intervention activities could involve a wide variety of initiatives—but especially cultural or spiritual activities—to restore and strengthen connections within and between families. Community-level suicide prevention activities might focus on bolstering the fabric of community by creating places and reasons to gather, unite, and build connection. This could be achieved through a variety of community-driven activities, for example via sports, competitions, ceremony, celebrations, gardening, learning programs, traditional land use, medicine picking, community groups, advocacy, and self-determination efforts [62].

This gap was also observed within program evaluations examined for this review, the majority of which did not incorporate any type of community-level outcome measure. The rare few that did attempt to measure, for example, community connectedness via social network characteristics [93], changes in community readiness for suicide prevention [39], or community-level changes in suicide and self-harm behaviours [59]. No evaluations included family-level outcome measures. Overall, we found that efforts to include family- and community-level initiatives within suicide prevention and evaluation did not adequately meet the need or sufficiently answer the consistent calls for such considerations from Indigenous communities [53, 83, 85]. Future suicide prevention development and implementation must do more to address this need.

In this review, we examined the ways in which Indigenous approaches have been incorporated into suicide prevention targeting Indigenous populations and the resulting impacts. Incorporation of Indigenous culture and knowledge as well as decolonizing efforts into suicide prevention was consistently shown to have positive implications for suicide-related outcomes. The meaningful inclusion of these components into suicide prevention, however, is dependent on the extent to which community self-determination is respected and upheld. From the articles reviewed here, which primarily involved partnerships between Western institutions and Indigenous communities, self-determination was best upheld where community engagement efforts were employed from before the development of a prevention program began all the way through implementation and evaluation to the finish. These engagement processes—especially those utilizing community-based participatory research (CBPR) approaches—also had a particular focus on restructuring the relationships between power, knowledge production, and public health policy and practice [24, 55]. Suicide prevention strategies that employed such community-engaged approaches were transformative and privileged Indigenous knowledge, culture, language, and locally-driven strategies that were decolonizing in nature. Decades have come to pass with no alleviation of suicide rates within Indigenous populations in Canada, the United States, Australia, and New Zealand. Dominant Western approaches to suicide prevention by themselves have largely failed at addressing suicide in Indigenous populations [114]. Consequently, future suicide prevention development and implementation must endeavor to privilege self-determined Indigenous approaches that are developed via comprehensive community engagement processes. While our review examined protective factors for suicide prevention within Indigenous populations in Canada, the United States, Australia, and New Zealand, higher suicide rates among Indigenous peoples compared to non-Indigenous populations have also been reported in Latin American countries including Brazil, Peru, Colombia, and Chile [115]. However, there is limited research evidence on Indigenous suicide in Latin American countries. A possible explanation for this lack of research could be that suicide among Indigenous peoples in those countries is significantly underreported.

Many of the suicide prevention efforts incorporated an evaluation component to measure impacts on suicide-related outcomes and were able to demonstrate some level of evidence for effectiveness. However, few evaluations were scientifically and methodologically rigorous [83, 116, 117]. In a systematic review of evaluations of suicide prevention targeting Indigenous populations and critique of their methodological quality, authors point out that evaluations were vulnerable to bias; heavily focused on proximal outcomes versus intermediate or distal outcomes; measured proxy outcomes like hopelessness and depression while ignoring suicide and self-harm behaviours entirely; and failed to consider cost [117]. In addition, it has been noted that there are considerable challenges related to suicide surveillance, including that national health data systems often lack Indigenous identifiers, do not capture data from some regions, and do not routinely engage Indigenous communities in data governance [118]. While we recognize that evaluation methodologies are heavily grounded in Western ways of knowing and constructs of “value” [119], evaluation outcomes are often crucial for justifying to funders (most often Western/colonial institutions) for continued resources for programming [117]. Moreover, evaluation can provide valuable insight into program goals, activities, strengths, areas for improvement, and cost-effectiveness [120, 121] and do not have to rely purely on Western epistemologies.

Practices to uphold Indigenous self-determination in suicide prevention (i.e. community engaged, participatory approaches) should extend to the evaluation phase in order to ensure outcomes measured are also meaningful to community and reflect Indigenous worldviews and ways of knowing [119, 121]. Evaluation processes that are culturally inclusive and community-driven have a number of crucial benefits. For instance, community-engaged suicide prevention evaluation ensures the needs and knowledge of the community remain central and community ownership, control, access and possession of evaluation outcomes and surveillance data are safeguarded. This includes how outcomes and surveillance data are shared, mitigating potential for harm that Indigenous communities have faced with non-inclusive research and surveillance [119, 122]. Unfortunately, most of the suicide prevention initiatives examined for this scoping review did not extend community engagement processes to the evaluation phase and thus failed to develop measures and evaluation tools based in Indigenous knowledge, focused on community-level outcome measures, or inclusive of Indigenous culture. Future work involving suicide prevention evaluation should ensure processes of community engagement are incorporated.

This critical scoping review is limited in two ways. Firstly, scoping studies provide a narrative or descriptive account of research and do not seek to appraise their quality or effectiveness [29]. Thus, in the context of this paper, which seeks to describe how Indigenous approaches have been integrated into suicide prevention, we do not evaluate individual program content, approaches to establishing programs, their methods, or their impacts. Instead, in employing a critical lens to this review, we sought to examine underlying structures of power and meaning that organize relationships, institutions, and knowledge production in suicide prevention, how Indigenous peoples are challenging these structures, and the resultant impacts on suicide outcomes [86] Secondly, this study is limited to the review of public-facing, written Indigenous suicide prevention only. Further exploration into informal, oral, and/or non-publicly facing initiatives would be required to create a more comprehensive picture of how Indigenous peoples are intervening on suicide in their communities.

## Conclusions

The urgent need to reduce the disproportionately high rates of suicide in Indigenous populations of Australia, New Zealand, Canada and the United States has been widely acknowledged. Conventional Western approaches to suicide prevention by themselves have largely failed at addressing this disproportionate burden. On the other hand, initiatives that are built upon comprehensive community engagement processes and which incorporate Indigenous culture, knowledge, and decolonizing methods have been shown to have substantial impact on suicide-related outcomes in the communities in which they are employed. Indigenous approaches to suicide prevention are diverse, drawing on local culture, knowledge, need and priorities. Nevertheless, substantial barriers persist to implementing such strategies. Existing funding and health service provision systems operate from a Western, biomedical model and may be unable or unwilling to provide the resources required to invest in Indigenous approaches, highlighting the importance of evaluation to establish efficacy. On a practical level, the findings from this critical scoping review may be useful to Indigenous communities and their partners to inform their own endeavours to develop and implement suicide prevention. We hope that the outcomes presented here encourage funders, health promotion experts, and government decision-makers to support community-driven, Indigenous-approaches to suicide prevention.

## Supplementary Information

**Additional file 1: Appendix A.** Academic Literature Search Strategy and Terms by Database.

**Additional file 2: Appendix B.** Academic literature data extraction table [123, 124].

**Additional file 3: Appendix C.** Summary of grey literature articles [125–130].

## Data Availability

The datasets used and/or analyzed during the current study are available from the corresponding author on reasonable request.
