# Supplementary material for: Centering Indigenous knowledge in suicide prevention: a critical scoping review
Source: BMC Public Health. 2022 Dec 19;22:2377. doi: 10.1186/s12889-022-14580-0 (PMC9761945; doi:10.1186/s12889-022-14580-0)
Supplement: Supplementary file 1 — Additional file 1: Appendix A. Academic Literature Search Strategy and Terms by Database. [file 12889_2022_14580_MOESM1_ESM.docx]

**APPENDIX A: Academic Literature Search Strategy and Terms by Database**

**Medline**

| # | Search Statement | Results |
| --- | --- | --- |
| 1 | (suicid* or "self harm").mp. or exp Suicide/ [mp=title, abstract, original title, name of substance word, subject heading word, floating sub-heading word, keyword heading word, organism supplementary concept word, protocol supplementary concept word, rare disease supplementary concept word, unique identifier, synonyms] | 106509 |
| 2 | (communit* or family or families or caregiver* or gatekeeper*).mp. [mp=title, abstract, original title, name of substance word, subject heading word, floating sub-heading word, keyword heading word, organism supplementary concept word, protocol supplementary concept word, rare disease supplementary concept word, unique identifier, synonyms] | 1920397 |
| 3 | [communication.mp](http://communication.mp/). or exp Communication/ or exp Health Communication/ | 597407 |
| 4 | exp Health Education/ or (awareness or prevent* or know* or educat* or train or trained or training).mp. | 5904039 |
| 5 | 3 or 4 | 6285976 |
| 6 | american native continental ancestry group/ or alaska natives/ or indians, central american/ or indians, north american/ or indians, south american/ or inuits/ or oceanic ancestry group/ or exp Health Services, Indigenous/ | 34512 |
| 7 | (Saami or Sampi or (Sami not Ulus) or Samis or Southernsami* or Umesami* or Pitesami* or Lulesami* or Northernsami* or Enaresami* or Kolasami* or Lapp or Lapps or Lappish or Lappland or (Lapland* not longspur) or Lappalainen* or Saamelainen* or reindeer herd* or reindeer culture* or reindeer pastoral* or Lappbys or Samebys or reinbeitesdistrikt or paliskunta or siida).mp. or (((Fennoscandia or Finnmark or Scandinavia or Nordic or Sweden or Norway or Finland or Swedish or Finnish or Norwegian or Norge or Svensk* or Suomi or Barents Region or (Kola not (garcinia or gotu)) or Arctic Europe* or Polar Europe* or North* Europ*).mp. or Finland/ or Norway/ or Sweden/) and ((traditional adj3 (food* or heal* or medicine* or shaman*)) or (Indigen* adj3 (people* or person* or mother* or father* or parent* or child* or boy or boys or girl* or youth* or healer* or patient* or famil* or herder*))).mp.) | 1496 |
| 8 | (Abenaki or Abenakis or Algonquin* or Algonquian* or Anishinabe* or Anishnabeg or Anishinaabe* or Assiniboine or Athapaskan or Beothuk* or Blackfoot or Chipewyan or Cree or Dogrib or Dene or Eskimo* or Esquimau* or Gwich'in or Haudenosaunee or Huron).mp. or exp Inuits/ or (Inuvaluit* or Inuit* or Innu or Innue or Innus or Inuk or (Iroquois not (corn or homeobox or transcription)) or Montagnais or Maliseet or Naskapi* or Micmac or Mic?Mac or Mi?gmaq or Mi?kmaq or Mic mac or Migmaw or Mig maw or Mohawk or Ojibw* or Sahtu or Salteaux or Saulteaux or Slavey or Tlicho or Yellowknives or (Peuple adj (autochtones or indidgenes or premier or racing or natif*)) or "Premiere Nation" or First Nation or First Nations or Metis or Mischif or Mitchif or Metif or Metchif or Bois-brule* or Mixed-blood* or Half Breed* or halfbreed* or (traditional adj1 (medicine* or heal* or food* or health*)) or Urban Indian* or "on reserve" or "off reserve" or country food* or shaman* or medicine m?n or medicine wom?n or ((native* or Indian or Indians) adj2 (person or persons or man or woman or men or women or child* or youth or youths or population* or people* or band or bands))).mp. [mp=title, abstract, original title, name of substance word, subject heading word, floating sub-heading word, keyword heading word, organism supplementary concept word, protocol supplementary concept word, rare disease supplementary concept word, unique identifier, synonyms] | 60959 |
| 9 | (Native American* or (Native adj2 (Alaska* or Hawaiian*)) or American Indian* or Amerindian* or (Absaroke or Alaskan Athabascans or Aleut or A'aninin or Anishinaabe or Aniyunwiya or (Apache not Apache II) or Arapaho or Arikara or Baxoje or Blackfeet or Bode'wadmi or Caddo or Cayuse or Chahta or Cherokee or Cheyenne or Chikasha or Chickasaw or Chippewa or Choctaw or Comanche or Cree or Cayuga or Dakelh or (Dine' not "Dine 1") or Eskimo* or Ewiiaapaayp or Gaigwu or Gayogohono or Gros Ventre or Havasupai or Hinonooeino or Haudenonsaunee or Hidatsa or Ho-Chunk or Hopi or Hualapai or Houma or Illiniwek or Illini or Iyiniwok or Ininiwok or (Iroquois not (homeobox or transcription)) or Kadohadacho or Kanienkehaka or Kanonsionni or Karok or Kickapoo or Kiowa or Kiwigapawa o Klickitat or Kumeyaay or Lanape or Lakota or Lumbee or Maidu or Maklak or Mamaceqtaw or Mandan or Maumee or Menominee or Meskwaki or Miccosukee or Mikasuki or Minisink or Mohawk or Mohegan or Mohican or Mohingan or Muheconneok or Munsee or Muskogee or Myaamia or Nakota or Nanigansek or Nantego or Narragansett or Navajo or Narragansett or Nde or Ndee or Niukonska or Numakiki or Numinu or Nunt'zi or Nuutsiu or Nuxbaaga or Odawa or Ojibway or Ojibwe or Ohkay Owingeh or Olekwo'l or Onandowaga or Oneida or Onondaga or Onundaga'ono or Onyota'aka or Osage or Pahoja or Panawahpskek or Passamaquoddy or Papago or Pend d'O reilles or Penobscot or Peskotomuhkati or Piaute or Pima or Pokanoket or Pomo or Ponca or Potawatomi or Powhatan or Po-wo-ge-oweenge or Pueblo or Puget Sound Salish or Quapaw or Qwulhhwaipum or Sahnish or Sauk or Sekani or Seminole or Shawnee or Shawanwa or Schitsu'msh or Shoshonee or Shuyelpee or Siksika or Skarooren or (Souix not "Souix Falls") or Ta-o-ta or Tetawken or Tete de Boule orTeton or Thlingchadine or Tohano O'odham or Tonkawa or Tlingit-Haida or Tohono or Tsalagi or Tsitsistas or Tuf-shum-tia or Tus-Tah or Tuscarora or O'odham or Ugakhpa or Umon'hon or Ute or Wampanoag or Wendat or Wihyot Winnebago or Who-ge-owenge or Wyandot or Yakima or Yaqui or Yavapai or Yurok or Yuman)).mp. | 29486 |
| 10 | ((((Acatec or Aguactec or Amuzgo or Chatino or Chiapaneca or Chichimeca or Chicomuceltec or Chinantec* or Chocho or Chontal or Chuj or Cochimi or Cocopa or Cuicatec* or Guarijio or Haurijio or Huastec* or Huave or Huichol or Ixcatec* or Jacaltec* or Kanjobal or Kaqchikel or Kekchi or K'iche' or Kickapoo or Kikapu or Kiliwa or Kumiai or Lacandon or Matlatzinca or Mazahua or Mazatec* or Mixe or Mixtec* or Mocho or Motocintleco or Nahua* or Oaxaca or Ocuiltec or Opata or Otomi or Paipai or Pame or Papago or Pima Bajo or Popoloca or Popoluca or (Purepecha not Echeveria) or Raramuri or Tabasco Chontal or Tacaneco or Tacuate or Tarahumara or Tectiteco or Teenek or Tenek or Tepehua or Tepehuan or Tlahuica or Tlapanec or "Tohono O'odham" or Tojolabal or Toltec or Totonac or Trique or Triqui or Tsotsil or (Tubar not ("tubar pregnancy" or "tubar sterility")) or Tzeltal or Tzotzil or Wixarika or Yaqui or Yucatec Maya or Zapotec or Zoque).tw. or ((Cora or Mame or Maya or Mayos or Mayo or Mam or Chol or Ch'ol or Ixil or Seri) adj3 (Indian* or tribe* or tribal or people* or person or elder or youth or child or children or men or men or man or woman or women or adolescent)).mp. or (((Mexico not New Mexico) or Aguascalientes or Baja California or "Los Cabos" or "La Paz" or Loreto or Campeche or Chiapas or Chihuahua or Coahuila or Colima or Manzanillo or Distrito Federal or Durango or "Estado de Mexico" or Guanajuato or "Leon San Miguel de Allende" or Guerrero or Acapulco or Hidalgo or Jalisco or Puerto Vallarta or Guadalajara or Michoacan or Morelia or Morelos or Nayarit or Nuevo Leon or Oaxaca or Huatulco or "Puerto Escondido" or Puebla or Queretaro or Quintana Roo or Cancun or Cozumel or "San Luis Potosi" or Sinaloa or Sonora or Tabasco or Tamaulipas or Tlaxcala or Veracruz or Yucatan or Merida or Zacatecas or Zenacantan* or Zenacanteco*) adj7 (("pre Columbian" adj3 culture*) or (preColumbian adj3 culture*) or Indian or Indians or Aboriginal* or Indigen* or "First Peoples" or "Native Mexican*" or "indos mexicano: or "pueblos indigenas" or shaman* or tribe or tribal or tribes or Amerindian* or traditional or Mesoamerindian*)).tw.) not ((Blinder adj2 Oaxaca) or Guatemala or Pepper Huasteco Virus or opata m85 or polar or cladoceran*).mp.) or ((Indians, North American/ or ((pre Columbian adj3 culture*) or (preColumbian adj3 culture*) or Indian or Indians or Aboriginal* or Indigen* or First Peoples or Native Mexican* or indos mexicano or pueblos indigenas or shaman* or h'iloletik or curander* or curandeiro or yerberos or herbalist* or hueseros or parteras or oracionistas or tabaqueros or ayahausqueros or peyoteros or sobadores or espiritualistas or tribe or tribal or tribes or Amerindian* or traditional or Mesoamerindian*).mp.) and (Mexico/ or (Mexican or mexico or mexico's or mexicano or mexicali).mp.))) not (exp animals/ not (exp humans/ and exp animals/)) | 6162 |
| 11 | (Abipon or Achuar or Achuagua or Akawaio or Amarizana or Andoque or Akawaio or Akuriyo or Anauya or Araona or Arawak or Ayamn or Aguaruna or Amahuaca or Amarakaeri or Andoa or Arabela or Arawak or Arhuaco or Ashaninca or Asheninca or Atsahuaca or Aymara or Ayoreo or Bakairi or Baniva or Barasana or Baniwa or Baure or Bororo or Cabiyari or Cacataibo or Caquinte or Cacua or Cahuarano or Caiua or Camara Indians or Camaracoto or Camsa or Canamari or Candoshi or Canela or Canichana or Capanahua or Carapana or Cariay or Carib or Carijona or Carutana or Cashibo or Cashinahua or Cawishana or Cavinena or Caxuiana or Cayuvava or Chontaquiro or Cocama or Cubeo or Curipaco or Chacobo or Chaima or (Chana not striatus) or Chapacura or Charrua or Chimila or Chitonahua or Chorote or Chipaya or Chiquitano or Chulupi or Carare or Coconuco or Cofan or Coreguaje or Coyaima or Chamacoco or Chamicuro or Chayahuita or Cocama or Culina or Culino or Cubeo or Cuiba or Cuiva or Cumanagoto or Curripaco or Deni or Desano or Embera or Guarani or Guajajara or Guana or Guanano or Guarayo or Guarayu or Guahibo or Guajiro or Guambiano or Guanano or Guayabero or Guarequena or Guinao or Guana or Gayon or Guahibo or Hixkaryana or Huachipairi or Huambisa or Huarayo or Iauanaua or Ikpeng or Ingariko or Irantxe or Itonama or Inapari or Iquito or Isconahua or Jumana or Japreria or Jirajara or Juruti or Jaqaru or Jebero or Kadiweu or Kaingang or Kamayura or Karaja or Karipuna or Kariri or Katukina or Kaxarari or Kayabi or Kayapo or Kuikuro alapalo or Kulina or Kaiwa or Kallawaya or Kogui or Kuna or Kaweskar or Lule or Macuna or Maipure or Mapuche or Mataco or Mocovi or Machinere or Machinerev or Machiguenga or Macushi or Macuna or Madi or Malayo or Mamainde or Manao or Mandauaca or Mandawaka or Mapidian or Mapuche or Mapidian or Maquiritare or Maquiritari or Maragua or Marawan or Mariate or Marubo or Mastanahua or Matipuhy or Matis or Matses or Mawakua or Mawakwa or Maxakali or Mehinaku or Miranha or Moronawa or Munduruku or Mataco or Movima or Muellama or Muinane or Mapoyo or Mashco Piro or Matses or Muniche or Nambikwara or Nocaman or Nuquini or Nomatsiguenga or Nanti or Ocaina or Omagua or Orejon or Opon or Pacahuara or Paez or Paicone or Palicur or Panare or Pano or Paresi or Paumari or Pemon or Pilaga or Puelche or Pauna or Pauserna or Piapoco or Piraha or Piratapuyo or Pisabo or Piaroa or Pijao or Piratapuyo or Paraujano or Pemon or Pemono or Piapoco or Puinave or Patamona or Poyanawa or Puinave or Puquina or Quechua or Quichua or Retuara or Resigaro or Reyesano or Sabanes or Saliba or Saluma or Sarave or Secoya or Selknam or Sensi or Shaninawa or Shapra or Sharanahua or Shebayo or Shiwiar or Shikiana or Sikiana or Siriono or Sinsiga or Siona or Suruwaha or Tacano or Tamanaco or Tiahuanaco or Tariano or Tehuelche or Tariano or Tatuyo or Tembe or Terena or Telembi or Ticuna or Ticuna or Tiriyo or Tiwanaku or Tiwanaku or Torom or Totoro or Tsimane or Tuberao or Tucano or Tunebo or Tuxinawa or Tuyuca or Uainuma or Urarina or Vilela or Waimaha or Waiampi or Waiwai or Wapishana or Waraiku or Warekena or Waura or Wayampi or Wayana or Wirina or Waimaha or Waunana or Wiwa or Warao or Wayuu or Witoto or Xavante or Xipaya or Xiriana or Xokleng or Yabaana or Yaminawa or Yaminahua or Yaruma or Yawalapiti or Yuracare or Yabarana or Yavitero or Yine or Yamana or Yaghan or Yucuna or Yurumangui or Yukpa or Yanesha or Yoranahua or Yagua or Yaminahua or Zaparo or Zamuco or ((Inga or Maca or Leco or Mojo or Uro or Maco or Lengua or Toba or Zoe or Ona or Catio or Passe or Bari or Awa or Bora or Bara or Remo or Pano or Sape) adj3 (Indians or Indian or Indigenous or Amerindian* or Aborigin* or people or peoples or women or men or woman or man or child* or youth or youths or baby or babies or tribe or tribes or tribal or shaman* or native or traditional)) or Trio Indians or More Indians or Bare Indians).mp. or ((Indian* or Amerind* or Aboriginal* or indigenas or Indigenous) and (Argentin* or Bolivia* or Brazil* or Chile* or Colombia* or French Guiana* or Guyana* or Peru or Paraguay or Uruguay or Venezuela or Amazon or Amazons or Amazonia or Andes or Andean)).tw. | 7400 |
| 12 | (Queensland or New South Wales or NSW or Northern Territory or Canberra or (Sydney not Canada) or ((Melbourne not (England or United Kingdom)) or Adelaide or Tasmania or (Perth not Scotland) or Austral*)).mp. and ((Indigen* or Aborig* or tribe or tribal or tribes or traditional or remote or outback or Blackfella* or Aborigin* or Indigenous* or first people* or original people).ti,ab. or Pacific Islander*.mp. or Ngunnawal.mp. or Murrawarri.mp. or Alyawarre.mp. or Anmatjera.mp. or Arrernte.mp. or Gurindiji.mp. or Kunibidji.mp. or Luritja.mp. or Murrinh Patha.mp. or Pitjantjatjara.mp. or Tiwi.mp. or Waripiri.mp. or Yoingu.mp. or Guugu Yimithirr.mp. or Kalkadoon.mp. or Torres Strait Islander*.mp. or Adnyamathanha.mp. or Adynyamathanha.mp. or Dieri.mp. or Kaurna.mp. or Maralinga Tjarutja.mp. or Ngarrindjeri.mp. or Narungga.mp. or Gunai.mp. or Kurnai.mp. or Kulin.mp. or Yorta Yorta.mp. or Bangerang.mp. or Kailtheban.mp. or Wollithiga.mp. or Moira.mp. or Ulupna.mp. or Kwat Kwat.mp. or Yalaba Yalaba.mp. or Ngurai illiam [wurrung.mp](http://wurrung.mp/). or Jarrakan.mp. or Noongar.mp. or Nyungar.mp. or Nyoongar.mp. or Pila Iguru.mp.) [mp=title, abstract, original title, name of substance word, subject heading word, floating sub-heading word, keyword heading word, organism supplementary concept word, protocol supplementary concept word, rare disease supplementary concept word, unique identifier, synonyms] | 16764 |
| 13 | ((maori or tangata whenua or mauori or moriori or mauri).mp. or ((New Zealand or Christchurch or Aukland).mp. or exp New Zealand/)) and (indigenous or aboriginal or "first people*" or shaman* or tribe or tribes or tribal or clan or clans).mp. | 1775 |
| 14 | or/6-13 | 120311 |
| 15 | 1 and 2 and 5 and 14 | 394 |
| 16 | 15 not (exp Africa/ or [africa.mp](http://africa.mp/). or Iran/ or [iran.mp](http://iran.mp/).) | 387 |
| 17 | (2019052* or 201906* or 201907* or 201908* or 201909* or 20191* or 2020* or 2021*).dt,ez,da. | 4385365 |
| 18 | 16 and 17 | 98 |

**Embase <1974 to 2021 November 19>**

| # | Search Statement | Results |
| --- | --- | --- |
| 1 | australoid/ or exp indigenous people/ or exp oceanic ancestry group/ | 34816 |
| 2 | indigenous people/ or alaska native/ or american indian/ or canadian aboriginal/ or first nation/ or indigenous australian/ or taiwanese aborigine/ | 29220 |
| 3 | oceanic ancestry group/ or pacific islander/ or torres strait islander/ | 8252 |
| 4 | exp amerind people/ or exp australian aborigine/ or exp austroasiatic people/ or exp austronesian people/ or exp eskimo-aleut people/ or exp na-dene people/ or exp sino-tibetan people/ | 18636 |
| 5 | (Saami or Sampi or (Sami not Ulus) or Samis or Southernsami* or Umesami* or Pitesami* or Lulesami* or Northernsami* or Enaresami* or Kolasami* or Lapp or Lapps or Lappish or Lappland or (Lapland* not longspur) or Lappalainen* or Saamelainen* or reindeer herd* or reindeer culture* or reindeer pastoral* or Lappbys or Samebys or reinbeitesdistrikt or paliskunta or siida).mp. or (((Fennoscandia or Finnmark or Scandinavia or Nordic or Sweden or Norway or Finland or Swedish or Finnish or Norwegian or Norge or Svensk* or Suomi or Barents Region or (Kola not (garcinia or gotu)) or Arctic Europe* or Polar Europe* or North* Europ*).mp. or Finland/ or Norway/ or Sweden/) and ((traditional adj3 (food* or heal* or medicine* or shaman*)) or (Indigen* adj3 (people* or person* or mother* or father* or parent* or child* or boy or boys or girl* or youth* or healer* or patient* or famil* or herder*))).mp.) | 1927 |
| 6 | (Abenaki or Abenakis or Algonquin* or Algonquian* or Anishinabe* or Anishnabeg or Anishinaabe* or Assiniboine or Athapaskan or Beothuk* or Blackfoot or Chipewyan or Cree or Dogrib or Dene or Eskimo* or Esquimau* or Gwich'in or Haudenosaunee or Huron).mp. or exp Inuits/ or (Inuvaluit* or Inuit* or Innu or Innue or Innus or Inuk or (Iroquois not (corn or homeobox or transcription)) or Montagnais or Maliseet or Naskapi* or Micmac or Mic?Mac or Mi?gmaq or Mi?kmaq or Mic mac or Migmaw or Mig maw or Mohawk or Ojibw* or Sahtu or Salteaux or Saulteaux or Slavey or Tlicho or Yellowknives or (Peuple adj (autochtones or indidgenes or premier or racing or natif*)) or "Premiere Nation" or First Nation or First Nations or Metis or Mischif or Mitchif or Metif or Metchif or Bois-brule* or Mixed-blood* or Half Breed* or halfbreed* or (traditional adj1 (medicine* or heal* or food* or health*)) or Urban Indian* or "on reserve" or "off reserve" or country food* or shaman* or medicine m?n or medicine wom?n or ((native* or Indian or Indians) adj2 (person or persons or man or woman or men or women or child* or youth or youths or population* or people* or band or bands))).mp. | 88922 |
| 7 | (Native American* or (Native adj2 (Alaska* or Hawaiian*)) or American Indian* or Amerindian* or (Absaroke or Alaskan Athabascans or Aleut or A'aninin or Anishinaabe or Aniyunwiya or (Apache not Apache II) or Arapaho or Arikara or Baxoje or Blackfeet or Bode'wadmi or Caddo or Cayuse or Chahta or Cherokee or Cheyenne or Chikasha or Chickasaw or Chippewa or Choctaw or Comanche or Cree or Cayuga or Dakelh or (Dine' not "Dine 1") or Eskimo* or Ewiiaapaayp or Gaigwu or Gayogohono or Gros Ventre or Havasupai or Hinonooeino or Haudenonsaunee or Hidatsa or Ho-Chunk or Hopi or Hualapai or Houma or Illiniwek or Illini or Iyiniwok or Ininiwok or (Iroquois not (homeobox or transcription)) or Kadohadacho or Kanienkehaka or Kanonsionni or Karok or Kickapoo or Kiowa or Kiwigapawa o Klickitat or Kumeyaay or Lanape or Lakota or Lumbee or Maidu or Maklak or Mamaceqtaw or Mandan or Maumee or Menominee or Meskwaki or Miccosukee or Mikasuki or Minisink or Mohawk or Mohegan or Mohican or Mohingan or Muheconneok or Munsee or Muskogee or Myaamia or Nakota or Nanigansek or Nantego or Narragansett or Navajo or Narragansett or Nde or Ndee or Niukonska or Numakiki or Numinu or Nunt'zi or Nuutsiu or Nuxbaaga or Odawa or Ojibway or Ojibwe or Ohkay Owingeh or Olekwo'l or Onandowaga or Oneida or Onondaga or Onundaga'ono or Onyota'aka or Osage or Pahoja or Panawahpskek or Passamaquoddy or Papago or Pend d'O reilles or Penobscot or Peskotomuhkati or Piaute or Pima or Pokanoket or Pomo or Ponca or Potawatomi or Powhatan or Po-wo-ge-oweenge or Pueblo or Puget Sound Salish or Quapaw or Qwulhhwaipum or Sahnish or Sauk or Sekani or Seminole or Shawnee or Shawanwa or Schitsu'msh or Shoshonee or Shuyelpee or Siksika or Skarooren or (Souix not "Souix Falls") or Ta-o-ta or Tetawken or Tete de Boule orTeton or Thlingchadine or Tohano O'odham or Tonkawa or Tlingit-Haida or Tohono or Tsalagi or Tsitsistas or Tuf-shum-tia or Tus-Tah or Tuscarora or O'odham or Ugakhpa or Umon'hon or Ute or Wampanoag or Wendat or Wihyot Winnebago or Who-ge-owenge or Wyandot or Yakima or Yaqui or Yavapai or Yurok or Yuman)).mp. | 47957 |
| 8 | ((((Acatec or Aguactec or Amuzgo or Chatino or Chiapaneca or Chichimeca or Chicomuceltec or Chinantec* or Chocho or Chontal or Chuj or Cochimi or Cocopa or Cuicatec* or Guarijio or Haurijio or Huastec* or Huave or Huichol or Ixcatec* or Jacaltec* or Kanjobal or Kaqchikel or Kekchi or K'iche' or Kickapoo or Kikapu or Kiliwa or Kumiai or Lacandon or Matlatzinca or Mazahua or Mazatec* or Mixe or Mixtec* or Mocho or Motocintleco or Nahua* or Oaxaca or Ocuiltec or Opata or Otomi or Paipai or Pame or Papago or Pima Bajo or Popoloca or Popoluca or (Purepecha not Echeveria) or Raramuri or Tabasco Chontal or Tacaneco or Tacuate or Tarahumara or Tectiteco or Teenek or Tenek or Tepehua or Tepehuan or Tlahuica or Tlapanec or Tohono O'odham or Tojolabal or Toltec or Totonac or Trique or Triqui or Tsotsil or (Tubar not ("tubar pregnancy" or "tubar sterility")) or Tzeltal or Tzotzil or Wixarika or Yaqui or Yucatec Maya or Zapotec or Zoque).tw. or ((Cora or Mame or Maya or Mayos or Mayo or Mam or Chol or Ch'ol or Ixil or Seri) adj3 (Indian* or tribe* or tribal or people* or person or elder or youth or child or children or men or men or man or woman or women or adolescent)).mp. or (((Mexico not New Mexico) or Aguascalientes or Baja California or "Los Cabos" or "La Paz" or Loreto or Campeche or Chiapas or Chihuahua or Coahuila or Colima or Manzanillo or Distrito Federal or Durango or "Estado de Mexico" or Guanajuato or "Leon San Miguel de Allende" or Guerrero or Acapulco or Hidalgo or Jalisco or Puerto Vallarta or Guadalajara or Michoacan or Morelia or Morelos or Nayarit or Nuevo Leon or Oaxaca or Huatulco or Puerto Escondido or Puebla or Queretaro or Quintana Roo or Cancun or Cozumel or San Luis Potosi or Sinaloa or Sonora or Tabasco or Tamaulipas or Tlaxcala or Veracruz or Yucatan or Merida or Zacatecas or Zenacantan* or Zenacanteco*) adj7 ((pre Columbian adj3 culture*) or (preColumbian adj3 culture*) or Indian or Indians or Aboriginal* or Indigen* or First Peoples or Native Mexican* or indos mexicano or pueblos indigenas or shaman* or tribe or tribal or tribes or Amerindian* or traditional or Mesoamerindian*)).tw.) not ((Blinder adj2 Oaxaca) or Guatemala or Pepper Huasteco Virus or opata m85 or polar or cladoceran*).mp.) or ((Indians, North American/ or ((pre Columbian adj3 culture*) or (preColumbian adj3 culture*) or Indian or Indians or Aboriginal* or Indigen* or First Peoples or Native Mexican* or indos mexicano or pueblos indigenas or shaman* or h'iloletik or curander* or curandeiro or yerberos or herbalist* or hueseros or parteras or oracionistas or tabaqueros or ayahausqueros or peyoteros or sobadores or espiritualistas or tribe or tribal or tribes or Amerindian* or traditional or Mesoamerindian*).mp.) and (Mexico/ or (Mexican or mexico or mexico's or mexicano or mexicali).mp.))) not (exp animals/ not (exp humans/ and exp animals/)) | 8529 |
| 9 | (Abipon or Achuar or Achuagua or Akawaio or Amarizana or Andoque or Akawaio or Akuriyo or Anauya or Araona or Arawak or Ayamn or Aguaruna or Amahuaca or Amarakaeri or Andoa or Arabela or Arawak or Arhuaco or Ashaninca or Asheninca or Atsahuaca or Aymara or Ayoreo or Bakairi or Baniva or Barasana or Baniwa or Baure or Bororo or Cabiyari or Cacataibo or Caquinte or Cacua or Cahuarano or Caiua or Camara Indians or Camaracoto or Camsa or Canamari or Candoshi or Canela or Canichana or Capanahua or Carapana or Cariay or Carib or Carijona or Carutana or Cashibo or Cashinahua or Cawishana or Cavinena or Caxuiana or Cayuvava or Chontaquiro or Cocama or Cubeo or Curipaco or Chacobo or Chaima or (Chana not striatus) or Chapacura or Charrua or Chimila or Chitonahua or Chorote or Chipaya or Chiquitano or Chulupi or Carare or Coconuco or Cofan or Coreguaje or Coyaima or Chamacoco or Chamicuro or Chayahuita or Cocama or Culina or Culino or Cubeo or Cuiba or Cuiva or Cumanagoto or Curripaco or Deni or Desano or Embera or Guarani or Guajajara or Guana or Guanano or Guarayo or Guarayu or Guahibo or Guajiro or Guambiano or Guanano or Guayabero or Guarequena or Guinao or Guana or Gayon or Guahibo or Hixkaryana or Huachipairi or Huambisa or Huarayo or Iauanaua or Ikpeng or Ingariko or Irantxe or Itonama or Inapari or Iquito or Isconahua or Jumana or Japreria or Jirajara or Juruti or Jaqaru or Jebero or Kadiweu or Kaingang or Kamayura or Karaja or Karipuna or Kariri or Katukina or Kaxarari or Kayabi or Kayapo or Kuikuro alapalo or Kulina or Kaiwa or Kallawaya or Kogui or Kuna or Kaweskar or Lule or Macuna or Maipure or Mapuche or Mataco or Mocovi or Machinere or Machinerev or Machiguenga or Macushi or Macuna or Madi or Malayo or Mamainde or Manao or Mandauaca or Mandawaka or Mapidian or Mapuche or Mapidian or Maquiritare or Maquiritari or Maragua or Marawan or Mariate or Marubo or Mastanahua or Matipuhy or Matis or Matses or Mawakua or Mawakwa or Maxakali or Mehinaku or Miranha or Moronawa or Munduruku or Mataco or Movima or Muellama or Muinane or Mapoyo or Mashco Piro or Matses or Muniche or Nambikwara or Nocaman or Nuquini or Nomatsiguenga or Nanti or Ocaina or Omagua or Orejon or Opon or Pacahuara or Paez or Paicone or Palicur or Panare or Pano or Paresi or Paumari or Pemon or Pilaga or Puelche or Pauna or Pauserna or Piapoco or Piraha or Piratapuyo or Pisabo or Piaroa or Pijao or Piratapuyo or Paraujano or Pemon or Pemono or Piapoco or Puinave or Patamona or Poyanawa or Puinave or Puquina or Quechua or Quichua or Retuara or Resigaro or Reyesano or Sabanes or Saliba or Saluma or Sarave or Secoya or Selknam or Sensi or Shaninawa or Shapra or Sharanahua or Shebayo or Shiwiar or Shikiana or Sikiana or Siriono or Sinsiga or Siona or Suruwaha or Tacano or Tamanaco or Tiahuanaco or Tariano or Tehuelche or Tariano or Tatuyo or Tembe or Terena or Telembi or Ticuna or Ticuna or Tiriyo or Tiwanaku or Tiwanaku or Torom or Totoro or Tsimane or Tuberao or Tucano or Tunebo or Tuxinawa or Tuyuca or Uainuma or Urarina or Vilela or Waimaha or Waiampi or Waiwai or Wapishana or Waraiku or Warekena or Waura or Wayampi or Wayana or Wirina or Waimaha or Waunana or Wiwa or Warao or Wayuu or Witoto or Xavante or Xipaya or Xiriana or Xokleng or Yabaana or Yaminawa or Yaminahua or Yaruma or Yawalapiti or Yuracare or Yabarana or Yavitero or Yine or Yamana or Yaghan or Yucuna or Yurumangui or Yukpa or Yanesha or Yoranahua or Yagua or Yaminahua or Zaparo or Zamuco or ((Inga or Maca or Leco or Mojo or Uro or Maco or Lengua or Toba or Zoe or Ona or Catio or Passe or Bari or Awa or Bora or Bara or Remo or Pano or Sape) adj3 (Indians or Indian or Indigenous or Amerindian* or Aborigin* or people or peoples or women or men or woman or man or child* or youth or youths or baby or babies or tribe or tribes or tribal or shaman* or native or traditional)) or Trio Indians or More Indians or Bare Indians).mp. or ((Indian* or Amerindian, or Aboriginal* or indigenas or Indigenous) and (Argentin* or Bolivia* or Brazil* or Chile* or Colombia* or French Guiana* or Guyana* or Peru or Paraguay or Uruguay or Venezuela or Amazon or Amazons or Amazonia or Andes or Andean)).tw. | 8408 |
| 10 | ((Queensland or New South Wales or NSW or Northern Territory or Canberra or (Sydney not Canada) or ((Melbourne not (England or United Kingdom)) or Adelaide or Tasmania or (Perth not Scotland) or Austral*)).mp. and ((Indigen* or Aborig* or tribe or tribal or tribes or traditional or remote or outback or Blackfella* or Aborigin* or Indigenous* or first people* or original people).ti,ab. or Torres Strait Island*.mp. or Ngunnawal.mp. or Murrawarri.mp. or Alyawarre.mp. or Anmatjera.mp. or Arrernte.mp. or Gurindiji.mp. or Kunibidji.mp. or Luritja.mp. or Murrinh Patha.mp. or Pitjantjatjara.mp. or Tiwi.mp. or Waripiri.mp. or Yoingu.mp. or Guugu Yimithirr.mp. or Kalkadoon.mp. or Torres Strait Islander*.mp. or Adnyamathanha.mp. or Adynyamathanha.mp. or Dieri.mp. or Kaurna.mp. or Maralinga Tjarutja.mp. or Ngarrindjeri.mp. or Narungga.mp. or Gunai.mp. or Kurnai.mp. or Kulin.mp. or Yorta Yorta.mp. or Bangerang.mp. or Kailtheban.mp. or Wollithiga.mp. or Moira.mp. or Ulupna.mp. or Kwat Kwat.mp. or Yalaba Yalaba.mp. or Ngurai illiam [wurrung.mp](http://wurrung.mp/). or Jarrakan.mp. or Noongar.mp. or Nyungar.mp. or Nyoongar.mp. or Pila Iguru.mp.)) or Torres Strait Islander*.mp. | 21620 |
| 11 | ((maori or tangata whenua or mauori or moriori or mauri).mp. or ((New Zealand or Christchurch or Aukland).mp. or exp New Zealand/)) and (indigenous or aboriginal or "first people*" or shaman* or tribe or tribes or tribal or clan or clans).mp. | 2573 |
| 12 | or/1-11 | 179066 |
| 13 | exp suicide/ or suicid*.mp. [mp=title, abstract, heading word, drug trade name, original title, device manufacturer, drug manufacturer, device trade name, keyword heading word, floating subheading word, candidate term word] | 142729 |
| 14 | (training or trained or train or educat* or know* or awareness).mp. [mp=title, abstract, heading word, drug trade name, original title, device manufacturer, drug manufacturer, device trade name, keyword heading word, floating subheading word, candidate term word] | 4888820 |
| 15 | (community or family or families or caregiver* or gatekeep*).mp. [mp=title, abstract, heading word, drug trade name, original title, device manufacturer, drug manufacturer, device trade name, keyword heading word, floating subheading word, candidate term word] | 2218144 |
| 16 | 12 and 13 and 14 and 15 | 235 |
| 17 | limit 16 to dc=20190524-20211130 | 54 |

**Global Health <1910 to 2021 Week 46>**

| # | Search Statement | Results |
| --- | --- | --- |
| 1 | indigenous people/ or alaska natives/ or american indians/ or inuit/ or exp pacific islanders/ | 15792 |
| 2 | (Saami or Sampi or (Sami not Ulus) or Samis or Southernsami* or Umesami* or Pitesami* or Lulesami* or Northernsami* or Enaresami* or Kolasami* or Lapp or Lapps or Lappish or Lappland or (Lapland* not longspur) or Lappalainen* or Saamelainen* or reindeer herd* or reindeer culture* or reindeer pastoral* or Lappbys or Samebys or reinbeitesdistrikt or paliskunta or siida).mp. or (((Fennoscandia or Finnmark or Scandinavia or Nordic or Sweden or Norway or Finland or Swedish or Finnish or Norwegian or Norge or Svensk* or Suomi or Barents Region or (Kola not (garcinia or gotu)) or Arctic Europe* or Polar Europe* or North* Europ*).mp. or Finland/ or Norway/ or Sweden/) and ((traditional adj3 (food* or heal* or medicine* or shaman*)) or (Indigen* adj3 (people* or person* or mother* or father* or parent* or child* or boy or boys or girl* or youth* or healer* or patient* or famil* or herder*))).mp.) | 927 |
| 3 | (Abenaki or Abenakis or Algonquin* or Algonquian* or Anishinabe* or Anishnabeg or Anishinaabe* or Assiniboine or Athapaskan or Beothuk* or Blackfoot or Chipewyan or Cree or Dogrib or Dene or Eskimo* or Esquimau* or Gwich'in or Haudenosaunee or Huron).mp. or exp Inuits/ or (Inuvaluit* or Inuit* or Innu or Innue or Innus or Inuk or (Iroquois not (corn or homeobox or transcription)) or Montagnais or Maliseet or Naskapi* or Micmac or Mic?Mac or Mi?gmaq or Mi?kmaq or Mic mac or Migmaw or Mig maw or Mohawk or Ojibw* or Sahtu or Salteaux or Saulteaux or Slavey or Tlicho or Yellowknives or (Peuple adj (autochtones or indidgenes or premier or racing or natif*)) or "Premiere Nation" or First Nation or First Nations or Metis or Mischif or Mitchif or Metif or Metchif or Bois-brule* or Mixed-blood* or Half Breed* or halfbreed* or (traditional adj1 (medicine* or heal* or food* or health*)) or Urban Indian* or "on reserve" or "off reserve" or country food* or shaman* or medicine m?n or medicine wom?n or ((native* or Indian or Indians) adj2 (person or persons or man or woman or men or women or child* or youth or youths or population* or people* or band or bands))).mp. [mp=abstract, title, original title, broad terms, heading words, identifiers, cabicodes] | 113905 |
| 4 | (Native American* or (Native adj2 (Alaska* or Hawaiian*)) or American Indian* or Amerindian* or (Absaroke or Alaskan Athabascans or Aleut or A'aninin or Anishinaabe or Aniyunwiya or (Apache not Apache II) or Arapaho or Arikara or Baxoje or Blackfeet or Bode'wadmi or Caddo or Cayuse or Chahta or Cherokee or Cheyenne or Chikasha or Chickasaw or Chippewa or Choctaw or Comanche or Cree or Cayuga or Dakelh or (Dine' not "Dine 1") or Eskimo* or Ewiiaapaayp or Gaigwu or Gayogohono or Gros Ventre or Havasupai or Hinonooeino or Haudenonsaunee or Hidatsa or Ho-Chunk or Hopi or Hualapai or Houma or Illiniwek or Illini or Iyiniwok or Ininiwok or (Iroquois not (homeobox or transcription)) or Kadohadacho or Kanienkehaka or Kanonsionni or Karok or Kickapoo or Kiowa or Kiwigapawa o Klickitat or Kumeyaay or Lanape or Lakota or Lumbee or Maidu or Maklak or Mamaceqtaw or Mandan or Maumee or Menominee or Meskwaki or Miccosukee or Mikasuki or Minisink or Mohawk or Mohegan or Mohican or Mohingan or Muheconneok or Munsee or Muskogee or Myaamia or Nakota or Nanigansek or Nantego or Narragansett or Navajo or Narragansett or Nde or Ndee or Niukonska or Numakiki or Numinu or Nunt'zi or Nuutsiu or Nuxbaaga or Odawa or Ojibway or Ojibwe or Ohkay Owingeh or Olekwo'l or Onandowaga or Oneida or Onondaga or Onundaga'ono or Onyota'aka or Osage or Pahoja or Panawahpskek or Passamaquoddy or Papago or Pend d'O reilles or Penobscot or Peskotomuhkati or Piaute or Pima or Pokanoket or Pomo or Ponca or Potawatomi or Powhatan or Po-wo-ge-oweenge or Pueblo or Puget Sound Salish or Quapaw or Qwulhhwaipum or Sahnish or Sauk or Sekani or Seminole or Shawnee or Shawanwa or Schitsu'msh or Shoshonee or Shuyelpee or Siksika or Skarooren or (Souix not "Souix Falls") or Ta-o-ta or Tetawken or Tete de Boule orTeton or Thlingchadine or Tohano O'odham or Tonkawa or Tlingit-Haida or Tohono or Tsalagi or Tsitsistas or Tuf-shum-tia or Tus-Tah or Tuscarora or O'odham or Ugakhpa or Umon'hon or Ute or Wampanoag or Wendat or Wihyot Winnebago or Who-ge-owenge or Wyandot or Yakima or Yaqui or Yavapai or Yurok or Yuman)).mp. | 9403 |
| 5 | (((Acatec or Aguactec or Amuzgo or Chatino or Chiapaneca or Chichimeca or Chicomuceltec or Chinantec* or Chocho or Chontal or Chuj or Cochimi or Cocopa or Cuicatec* or Guarijio or Haurijio or Huastec* or Huave or Huichol or Ixcatec* or Jacaltec* or Kanjobal or Kaqchikel or Kekchi or K'iche' or Kickapoo or Kikapu or Kiliwa or Kumiai or Lacandon or Matlatzinca or Mazahua or Mazatec* or Mixe or Mixtec* or Mocho or Motocintleco or Nahua* or Oaxaca or Ocuiltec or Opata or Otomi or Paipai or Pame or Papago or Pima Bajo or Popoloca or Popoluca or (Purepecha not Echeveria) or Raramuri or Tabasco Chontal or Tacaneco or Tacuate or Tarahumara or Tectiteco or Teenek or Tenek or Tepehua or Tepehuan or Tlahuica or Tlapanec or Tohono O'odham or Tojolabal or Toltec or Totonac or Trique or Triqui or Tsotsil or (Tubar not ("tubar pregnancy" or "tubar sterility")) or Tzeltal or Tzotzil or Wixarika or Yaqui or Yucatec Maya or Zapotec or Zoque).tw. or ((Cora or Mame or Maya or Mayos or Mayo or Mam or Chol or Ch'ol or Ixil or Seri) adj3 (Indian* or tribe* or tribal or people* or person or elder or youth or child or children or men or men or man or woman or women or adolescent)).mp. or (((Mexico not New Mexico) or Aguascalientes or Baja California or "Los Cabos" or "La Paz" or Loreto or Campeche or Chiapas or Chihuahua or Coahuila or Colima or Manzanillo or Distrito Federal or Durango or "Estado de Mexico" or Guanajuato or "Leon San Miguel de Allende" or Guerrero or Acapulco or Hidalgo or Jalisco or Puerto Vallarta or Guadalajara or Michoacan or Morelia or Morelos or Nayarit or Nuevo Leon or Oaxaca or Huatulco or Puerto Escondido or Puebla or Queretaro or Quintana Roo or Cancun or Cozumel or San Luis Potosi or Sinaloa or Sonora or Tabasco or Tamaulipas or Tlaxcala or Veracruz or Yucatan or Merida or Zacatecas or Zenacantan* or Zenacanteco*) adj7 ((pre Columbian adj3 culture*) or (preColumbian adj3 culture*) or Indian or Indians or Aboriginal* or Indigen* or First Peoples or Native Mexican* or indos mexicano or pueblos indigenas or shaman* or tribe or tribal or tribes or Amerindian* or traditional or Mesoamerindian*)).tw.) not ((Blinder adj2 Oaxaca) or Guatemala or Pepper Huasteco Virus or opata m85 or polar or cladoceran*).mp.) or ((Indians, North American/ or ((pre Columbian adj3 culture*) or (preColumbian adj3 culture*) or Indian or Indians or Aboriginal* or Indigen* or First Peoples or Native Mexican* or indos mexicano or pueblos indigenas or shaman* or h'iloletik or curander* or curandeiro or yerberos or herbalist* or hueseros or parteras or oracionistas or tabaqueros or ayahausqueros or peyoteros or sobadores or espiritualistas or tribe or tribal or tribes or Amerindian* or traditional or Mesoamerindian*).mp.) and (Mexico/ or (Mexican or mexico or mexico's or mexicano or mexicali).mp.)) | 4107 |
| 6 | (Abipon or Achuar or Achuagua or Akawaio or Amarizana or Andoque or Akawaio or Akuriyo or Anauya or Araona or Arawak or Ayamn or Aguaruna or Amahuaca or Amarakaeri or Andoa or Arabela or Arawak or Arhuaco or Ashaninca or Asheninca or Atsahuaca or Aymara or Ayoreo or Bakairi or Baniva or Barasana or Baniwa or Baure or Bororo or Cabiyari or Cacataibo or Caquinte or Cacua or Cahuarano or Caiua or Camara Indians or Camaracoto or Camsa or Canamari or Candoshi or Canela or Canichana or Capanahua or Carapana or Cariay or Carib or Carijona or Carutana or Cashibo or Cashinahua or Cawishana or Cavinena or Caxuiana or Cayuvava or Chontaquiro or Cocama or Cubeo or Curipaco or Chacobo or Chaima or (Chana not striatus) or Chapacura or Charrua or Chimila or Chitonahua or Chorote or Chipaya or Chiquitano or Chulupi or Carare or Coconuco or Cofan or Coreguaje or Coyaima or Chamacoco or Chamicuro or Chayahuita or Cocama or Culina or Culino or Cubeo or Cuiba or Cuiva or Cumanagoto or Curripaco or Deni or Desano or Embera or Guarani or Guajajara or Guana or Guanano or Guarayo or Guarayu or Guahibo or Guajiro or Guambiano or Guanano or Guayabero or Guarequena or Guinao or Guana or Gayon or Guahibo or Hixkaryana or Huachipairi or Huambisa or Huarayo or Iauanaua or Ikpeng or Ingariko or Irantxe or Itonama or Inapari or Iquito or Isconahua or Jumana or Japreria or Jirajara or Juruti or Jaqaru or Jebero or Kadiweu or Kaingang or Kamayura or Karaja or Karipuna or Kariri or Katukina or Kaxarari or Kayabi or Kayapo or Kuikuro alapalo or Kulina or Kaiwa or Kallawaya or Kogui or Kuna or Kaweskar or Lule or Macuna or Maipure or Mapuche or Mataco or Mocovi or Machinere or Machinerev or Machiguenga or Macushi or Macuna or Madi or Malayo or Mamainde or Manao or Mandauaca or Mandawaka or Mapidian or Mapuche or Mapidian or Maquiritare or Maquiritari or Maragua or Marawan or Mariate or Marubo or Mastanahua or Matipuhy or Matis or Matses or Mawakua or Mawakwa or Maxakali or Mehinaku or Miranha or Moronawa or Munduruku or Mataco or Movima or Muellama or Muinane or Mapoyo or Mashco Piro or Matses or Muniche or Nambikwara or Nocaman or Nuquini or Nomatsiguenga or Nanti or Ocaina or Omagua or Orejon or Opon or Pacahuara or Paez or Paicone or Palicur or Panare or Pano or Paresi or Paumari or Pemon or Pilaga or Puelche or Pauna or Pauserna or Piapoco or Piraha or Piratapuyo or Pisabo or Piaroa or Pijao or Piratapuyo or Paraujano or Pemon or Pemono or Piapoco or Puinave or Patamona or Poyanawa or Puinave or Puquina or Quechua or Quichua or Retuara or Resigaro or Reyesano or Sabanes or Saliba or Saluma or Sarave or Secoya or Selknam or Sensi or Shaninawa or Shapra or Sharanahua or Shebayo or Shiwiar or Shikiana or Sikiana or Siriono or Sinsiga or Siona or Suruwaha or Tacano or Tamanaco or Tiahuanaco or Tariano or Tehuelche or Tariano or Tatuyo or Tembe or Terena or Telembi or Ticuna or Ticuna or Tiriyo or Tiwanaku or Tiwanaku or Torom or Totoro or Tsimane or Tuberao or Tucano or Tunebo or Tuxinawa or Tuyuca or Uainuma or Urarina or Vilela or Waimaha or Waiampi or Waiwai or Wapishana or Waraiku or Warekena or Waura or Wayampi or Wayana or Wirina or Waimaha or Waunana or Wiwa or Warao or Wayuu or Witoto or Xavante or Xipaya or Xiriana or Xokleng or Yabaana or Yaminawa or Yaminahua or Yaruma or Yawalapiti or Yuracare or Yabarana or Yavitero or Yine or Yamana or Yaghan or Yucuna or Yurumangui or Yukpa or Yanesha or Yoranahua or Yagua or Yaminahua or Zaparo or Zamuco or ((Inga or Maca or Leco or Mojo or Uro or Maco or Lengua or Toba or Zoe or Ona or Catio or Passe or Bari or Awa or Bora or Bara or Remo or Pano or Sape) adj3 (Indians or Indian or Indigenous or Amerindian* or Aborigin* or people or peoples or women or men or woman or man or child* or youth or youths or baby or babies or tribe or tribes or tribal or shaman* or native or traditional)) or Trio Indians or More Indians or Bare Indians).mp. or ((Indian* or Amerindian, or Aboriginal* or indigenas or Indigenous) and (Argentin* or Bolivia* or Brazil* or Chile* or Colombia* or French Guiana* or Guyana* or Peru or Paraguay or Uruguay or Venezuela or Amazon or Amazons or Amazonia or Andes or Andean)).tw. | 7653 |
| 7 | ((Queensland or New South Wales or NSW or Northern Territory or Canberra or (Sydney not Canada) or ((Melbourne not (England or United Kingdom)) or Adelaide or Tasmania or (Perth not Scotland) or Austral*)).mp. and ((Indigen* or Aborig* or tribe or tribal or tribes or traditional or remote or outback or Blackfella* or Aborigin* or Indigenous* or first people* or original people).ti,ab. or Torres Strait Island*.mp. or Ngunnawal.mp. or Murrawarri.mp. or Alyawarre.mp. or Anmatjera.mp. or Arrernte.mp. or Gurindiji.mp. or Kunibidji.mp. or Luritja.mp. or Murrinh Patha.mp. or Pitjantjatjara.mp. or Tiwi.mp. or Waripiri.mp. or Yoingu.mp. or Guugu Yimithirr.mp. or Kalkadoon.mp. or Torres Strait Islander*.mp. or Adnyamathanha.mp. or Adynyamathanha.mp. or Dieri.mp. or Kaurna.mp. or Maralinga Tjarutja.mp. or Ngarrindjeri.mp. or Narungga.mp. or Gunai.mp. or Kurnai.mp. or Kulin.mp. or Yorta Yorta.mp. or Bangerang.mp. or Kailtheban.mp. or Wollithiga.mp. or Moira.mp. or Ulupna.mp. or Kwat Kwat.mp. or Yalaba Yalaba.mp. or Ngurai illiam [wurrung.mp](http://wurrung.mp/). or Jarrakan.mp. or Noongar.mp. or Nyungar.mp. or Nyoongar.mp. or Pila Iguru.mp.)) or Torres Strait Islander*.mp. | 9436 |
| 8 | ((maori or tangata whenua or mauori or moriori or mauri).mp. or ((New Zealand or Christchurch or Aukland).mp. or exp New Zealand/)) and (indigenous or aboriginal or "first people*" or shaman* or tribe or tribes or tribal or clan or clans).mp. | 1145 |
| 9 | 1 or 2 or 3 or 4 or 5 or 6 or 7 or 8 | 142947 |
| 10 | exp suicide/ or suicid*.mp. [mp=abstract, title, original title, broad terms, heading words, identifiers, cabicodes] | 12024 |
| 11 | (training or trained or train or educat* or know* or awareness).mp. [mp=abstract, title, original title, broad terms, heading words, identifiers, cabicodes] | 665174 |
| 12 | (community or family or families or caregiver* or gatekeep*).mp. [mp=abstract, title, original title, broad terms, heading words, identifiers, cabicodes] | 460875 |
| 13 | 9 and 10 and 11 and 12 | 69 |
| 14 | limit 13 to up=20190527-20211130 | 14 |

APA PsycInfo <1806 to November Week 3 2021>

| # | Search Statement | Results |
| --- | --- | --- |
| 1 | indigenous populations/ or alaska natives/ or american indians/ or inuit/ or exp pacific islanders/ | 14703 |
| 2 | (Saami or Sampi or (Sami not Ulus) or Samis or Southernsami* or Umesami* or Pitesami* or Lulesami* or Northernsami* or Enaresami* or Kolasami* or Lapp or Lapps or Lappish or Lappland or (Lapland* not longspur) or Lappalainen* or Saamelainen* or reindeer herd* or reindeer culture* or reindeer pastoral* or Lappbys or Samebys or reinbeitesdistrikt or paliskunta or siida).mp. or (((Fennoscandia or Finnmark or Scandinavia or Nordic or Sweden or Norway or Finland or Swedish or Finnish or Norwegian or Norge or Svensk* or Suomi or Barents Region or (Kola not (garcinia or gotu)) or Arctic Europe* or Polar Europe* or North* Europ*).mp. or Finland/ or Norway/ or Sweden/) and ((traditional adj3 (food* or heal* or medicine* or shaman*)) or (Indigen* adj3 (people* or person* or mother* or father* or parent* or child* or boy or boys or girl* or youth* or healer* or patient* or famil* or herder*))).mp.) | 523 |
| 3 | (Abenaki or Abenakis or Algonquin* or Algonquian* or Anishinabe* or Anishnabeg or Anishinaabe* or Assiniboine or Athapaskan or Beothuk* or Blackfoot or Chipewyan or Cree or Dogrib or Dene or Eskimo* or Esquimau* or Gwich'in or Haudenosaunee or Huron).mp. or exp Inuits/ or (Inuvaluit* or Inuit* or Innu or Innue or Innus or Inuk or (Iroquois not (corn or homeobox or transcription)) or Montagnais or Maliseet or Naskapi* or Micmac or Mic?Mac or Mi?gmaq or Mi?kmaq or Mic mac or Migmaw or Mig maw or Mohawk or Ojibw* or Sahtu or Salteaux or Saulteaux or Slavey or Tlicho or Yellowknives or (Peuple adj (autochtones or indidgenes or premier or racing or natif*)) or "Premiere Nation" or First Nation or First Nations or Metis or Mischif or Mitchif or Metif or Metchif or Bois-brule* or Mixed-blood* or Half Breed* or halfbreed* or (traditional adj1 (medicine* or heal* or food* or health*)) or Urban Indian* or "on reserve" or "off reserve" or country food* or shaman* or medicine m?n or medicine wom?n or ((native* or Indian or Indians) adj2 (person or persons or man or woman or men or women or child* or youth or youths or population* or people* or band or bands))).mp. [mp=title, abstract, heading word, table of contents, key concepts, original title, tests & measures, mesh] | 13384 |
| 4 | (Native American* or (Native adj2 (Alaska* or Hawaiian*)) or American Indian* or Amerindian* or (Absaroke or Alaskan Athabascans or Aleut or A'aninin or Anishinaabe or Aniyunwiya or (Apache not Apache II) or Arapaho or Arikara or Baxoje or Blackfeet or Bode'wadmi or Caddo or Cayuse or Chahta or Cherokee or Cheyenne or Chikasha or Chickasaw or Chippewa or Choctaw or Comanche or Cree or Cayuga or Dakelh or (Dine' not "Dine 1") or Eskimo* or Ewiiaapaayp or Gaigwu or Gayogohono or Gros Ventre or Havasupai or Hinonooeino or Haudenonsaunee or Hidatsa or Ho-Chunk or Hopi or Hualapai or Houma or Illiniwek or Illini or Iyiniwok or Ininiwok or (Iroquois not (homeobox or transcription)) or Kadohadacho or Kanienkehaka or Kanonsionni or Karok or Kickapoo or Kiowa or Kiwigapawa o Klickitat or Kumeyaay or Lanape or Lakota or Lumbee or Maidu or Maklak or Mamaceqtaw or Mandan or Maumee or Menominee or Meskwaki or Miccosukee or Mikasuki or Minisink or Mohawk or Mohegan or Mohican or Mohingan or Muheconneok or Munsee or Muskogee or Myaamia or Nakota or Nanigansek or Nantego or Narragansett or Navajo or Narragansett or Nde or Ndee or Niukonska or Numakiki or Numinu or Nunt'zi or Nuutsiu or Nuxbaaga or Odawa or Ojibway or Ojibwe or Ohkay Owingeh or Olekwo'l or Onandowaga or Oneida or Onondaga or Onundaga'ono or Onyota'aka or Osage or Pahoja or Panawahpskek or Passamaquoddy or Papago or Pend d'O reilles or Penobscot or Peskotomuhkati or Piaute or Pima or Pokanoket or Pomo or Ponca or Potawatomi or Powhatan or Po-wo-ge-oweenge or Pueblo or Puget Sound Salish or Quapaw or Qwulhhwaipum or Sahnish or Sauk or Sekani or Seminole or Shawnee or Shawanwa or Schitsu'msh or Shoshonee or Shuyelpee or Siksika or Skarooren or (Souix not "Souix Falls") or Ta-o-ta or Tetawken or Tete de Boule or Teton or Thlingchadine or Tohano O'odham or Tonkawa or Tlingit-Haida or Tohono or Tsalagi or Tsitsistas or Tuf-shum-tia or Tus-Tah or Tuscarora or O'odham or Ugakhpa or Umon'hon or Ute or Wampanoag or Wendat or Wihyot Winnebago or Who-ge-owenge or Wyandot or Yakima or Yaqui or Yavapai or Yurok or Yuman)).mp. | 15353 |
| 5 | (((Acatec or Aguactec or Amuzgo or Chatino or Chiapaneca or Chichimeca or Chicomuceltec or Chinantec* or Chocho or Chontal or Chuj or Cochimi or Cocopa or Cuicatec* or Guarijio or Haurijio or Huastec* or Huave or Huichol or Ixcatec* or Jacaltec* or Kanjobal or Kaqchikel or Kekchi or K'iche' or Kickapoo or Kikapu or Kiliwa or Kumiai or Lacandon or Matlatzinca or Mazahua or Mazatec* or Mixe or Mixtec* or Mocho or Motocintleco or Nahua* or Oaxaca or Ocuiltec or Opata or Otomi or Paipai or Pame or Papago or Pima Bajo or Popoloca or Popoluca or (Purepecha not Echeveria) or Raramuri or Tabasco Chontal or Tacaneco or Tacuate or Tarahumara or Tectiteco or Teenek or Tenek or Tepehua or Tepehuan or Tlahuica or Tlapanec or Tohono O'odham or Tojolabal or Toltec or Totonac or Trique or Triqui or Tsotsil or (Tubar not ("tubar pregnancy" or "tubar sterility")) or Tzeltal or Tzotzil or Wixarika or Yaqui or Yucatec Maya or Zapotec or Zoque).tw. or ((Cora or Mame or Maya or Mayos or Mayo or Mam or Chol or Ch'ol or Ixil or Seri) adj3 (Indian* or tribe* or tribal or people* or person or elder or youth or child or children or men or men or man or woman or women or adolescent)).mp. or (((Mexico not New Mexico) or Aguascalientes or Baja California or "Los Cabos" or "La Paz" or Loreto or Campeche or Chiapas or Chihuahua or Coahuila or Colima or Manzanillo or Distrito Federal or Durango or "Estado de Mexico" or Guanajuato or "Leon San Miguel de Allende" or Guerrero or Acapulco or Hidalgo or Jalisco or Puerto Vallarta or Guadalajara or Michoacan or Morelia or Morelos or Nayarit or Nuevo Leon or Oaxaca or Huatulco or Puerto Escondido or Puebla or Queretaro or Quintana Roo or Cancun or Cozumel or San Luis Potosi or Sinaloa or Sonora or Tabasco or Tamaulipas or Tlaxcala or Veracruz or Yucatan or Merida or Zacatecas or Zenacantan* or Zenacanteco*) adj7 ((pre Columbian adj3 culture*) or (preColumbian adj3 culture*) or Indian or Indians or Aboriginal* or Indigen* or First Peoples or Native Mexican* or indos mexicano or pueblos indigenas or shaman* or tribe or tribal or tribes or Amerindian* or traditional or Mesoamerindian*)).tw.) not ((Blinder adj2 Oaxaca) or Guatemala or Pepper Huasteco Virus or opata m85 or polar or cladoceran*).mp.) or ((Indians, North American/ or ((pre Columbian adj3 culture*) or (preColumbian adj3 culture*) or Indian or Indians or Aboriginal* or Indigen* or First Peoples or Native Mexican* or indos mexicano or pueblos indigenas or shaman* or h'iloletik or curander* or curandeiro or yerberos or herbalist* or hueseros or parteras or oracionistas or tabaqueros or ayahausqueros or peyoteros or sobadores or espiritualistas or tribe or tribal or tribes or Amerindian* or traditional or Mesoamerindian*).mp.) and (Mexico/ or (Mexican or mexico or mexico's or mexicano or mexicali).mp.)) | 3068 |
| 6 | (Abipon or Achuar or Achuagua or Akawaio or Amarizana or Andoque or Akawaio or Akuriyo or Anauya or Araona or Arawak or Ayamn or Aguaruna or Amahuaca or Amarakaeri or Andoa or Arabela or Arawak or Arhuaco or Ashaninca or Asheninca or Atsahuaca or Aymara or Ayoreo or Bakairi or Baniva or Barasana or Baniwa or Baure or Bororo or Cabiyari or Cacataibo or Caquinte or Cacua or Cahuarano or Caiua or "Camara Indians" or Camaracoto or Camsa or Canamari or Candoshi or Canela or Canichana or Capanahua or Carapana or Cariay or Carib or Carijona or Carutana or Cashibo or Cashinahua or Cawishana or Cavinena or Caxuiana or Cayuvava or Chontaquiro or Cocama or Cubeo or Curipaco or Chacobo or Chaima or (Chana not striatus) or Chapacura or Charrua or Chimila or Chitonahua or Chorote or Chipaya or Chiquitano or Chulupi or Carare or Coconuco or Cofan or Coreguaje or Coyaima or Chamacoco or Chamicuro or Chayahuita or Cocama or Culina or Culino or Cubeo or Cuiba or Cuiva or Cumanagoto or Curripaco or Deni or Desano or Embera or Guarani or Guajajara or Guana or Guanano or Guarayo or Guarayu or Guahibo or Guajiro or Guambiano or Guanano or Guayabero or Guarequena or Guinao or Guana or Gayon or Guahibo or Hixkaryana or Huachipairi or Huambisa or Huarayo or Iauanaua or Ikpeng or Ingariko or Irantxe or Itonama or Inapari or Iquito or Isconahua or Jumana or Japreria or Jirajara or Juruti or Jaqaru or Jebero or Kadiweu or Kaingang or Kamayura or Karaja or Karipuna or Kariri or Katukina or Kaxarari or Kayabi or Kayapo or "Kuikuro alapalo" or Kulina or Kaiwa or Kallawaya or Kogui or Kuna or Kaweskar or Lule or Macuna or Maipure or Mapuche or Mataco or Mocovi or Machinere or Machinerev or Machiguenga or Macushi or Macuna or Madi or Malayo or Mamainde or Manao or Mandauaca or Mandawaka or Mapidian or Mapuche or Mapidian or Maquiritare or Maquiritari or Maragua or Marawan or Mariate or Marubo or Mastanahua or Matipuhy or Matis or Matses or Mawakua or Mawakwa or Maxakali or Mehinaku or Miranha or Moronawa or Munduruku or Mataco or Movima or Muellama or Muinane or Mapoyo or "Mashco Piro" or Matses or Muniche or Nambikwara or Nocaman or Nuquini or Nomatsiguenga or Nanti or Ocaina or Omagua or Orejon or Opon or Pacahuara or Paez or Paicone or Palicur or Panare or Pano or Paresi or Paumari or Pemon or Pilaga or Puelche or Pauna or Pauserna or Piapoco or Piraha or Piratapuyo or Pisabo or Piaroa or Pijao or Piratapuyo or Paraujano or Pemon or Pemono or Piapoco or Puinave or Patamona or Poyanawa or Puinave or Puquina or Quechua or Quichua or Retuara or Resigaro or Reyesano or Sabanes or Saliba or Saluma or Sarave or Secoya or Selknam or Sensi or Shaninawa or Shapra or Sharanahua or Shebayo or Shiwiar or Shikiana or Sikiana or Siriono or Sinsiga or Siona or Suruwaha or Tacano or Tamanaco or Tiahuanaco or Tariano or Tehuelche or Tariano or Tatuyo or Tembe or Terena or Telembi or Ticuna or Ticuna or Tiriyo or Tiwanaku or Tiwanaku or Torom or Totoro or Tsimane or Tuberao or Tucano or Tunebo or Tuxinawa or Tuyuca or Uainuma or Urarina or Vilela or Waimaha or Waiampi or Waiwai or Wapishana or Waraiku or Warekena or Waura or Wayampi or Wayana or Wirina or Waimaha or Waunana or Wiwa or Warao or Wayuu or Witoto or Xavante or Xipaya or Xiriana or Xokleng or Yabaana or Yaminawa or Yaminahua or Yaruma or Yawalapiti or Yuracare or Yabarana or Yavitero or Yine or Yamana or Yaghan or Yucuna or Yurumangui or Yukpa or Yanesha or Yoranahua or Yagua or Yaminahua or Zaparo or Zamuco or ((Inga or Maca or Leco or Mojo or Uro or Maco or Lengua or Toba or Zoe or Ona or Catio or Passe or Bari or Awa or Bora or Bara or Remo or Pano or Sape) adj3 (Indians or Indian or Indigenous or Amerindian* or Aborigin* or people or peoples or women or men or woman or man or child* or youth or youths or baby or babies or tribe or tribes or tribal or shaman* or native or traditional)) or Trio Indians or More Indians or Bare Indians).mp. or ((Indian* or Amerindian, or Aboriginal* or indigenas or Indigenous) and (Argentin* or Bolivia* or Brazil* or Chile* or Colombia* or French Guiana* or Guyana* or Peru or Paraguay or Uruguay or Venezuela or Amazon or Amazons or Amazonia or Andes or Andean)).tw. | 2140 |
| 7 | ((Queensland or New South Wales or NSW or Northern Territory or Canberra or (Sydney not Canada) or ((Melbourne not (England or United Kingdom)) or Adelaide or Tasmania or (Perth not Scotland) or Austral*)).mp. and ((Indigen* or Aborig* or tribe or tribal or tribes or traditional or remote or outback or Blackfella* or Aborigin* or Indigenous* or "first people*" or "original people").ti,ab. or Torres Strait Island*.mp. or Ngunnawal.mp. or Murrawarri.mp. or Alyawarre.mp. or Anmatjera.mp. or Arrernte.mp. or Gurindiji.mp. or Kunibidji.mp. or Luritja.mp. or Murrinh Patha.mp. or Pitjantjatjara.mp. or Tiwi.mp. or Waripiri.mp. or Yoingu.mp. or Guugu Yimithirr.mp. or Kalkadoon.mp. or Torres Strait Islander*.mp. or Adnyamathanha.mp. or Adynyamathanha.mp. or Dieri.mp. or Kaurna.mp. or Maralinga Tjarutja.mp. or Ngarrindjeri.mp. or Narungga.mp. or Gunai.mp. or Kurnai.mp. or Kulin.mp. or Yorta Yorta.mp. or Bangerang.mp. or Kailtheban.mp. or Wollithiga.mp. or Moira.mp. or Ulupna.mp. or Kwat Kwat.mp. or Yalaba Yalaba.mp. or Ngurai illiam [wurrung.mp](http://wurrung.mp/). or Jarrakan.mp. or Noongar.mp. or Nyungar.mp. or Nyoongar.mp. or Pila Iguru.mp.)) or Torres Strait Islander*.mp. | 5704 |
| 8 | ((maori or tangata whenua or mauori or moriori or mauri).mp. or ((New Zealand or Christchurch or Aukland).mp. or exp New Zealand/)) and (indigenous or aboriginal or "first people*" or shaman* or tribe or tribes or tribal or clan or clans).mp. | 1087 |
| 9 | 1 or 2 or 3 or 4 or 5 or 6 or 7 or 8 | 37821 |
| 10 | exp suicide/ or suicid*.mp. [mp=title, abstract, heading word, table of contents, key concepts, original title, tests & measures, mesh] | 75073 |
| 11 | (training or trained or train or educat* or know* or awareness).mp. [mp=title, abstract, heading word, table of contents, key concepts, original title, tests & measures, mesh] | 1441205 |
| 12 | (communities or family or families or caregiver* or elder or eldersor gatekeep*).mp. [mp=title, abstract, heading word, table of contents, key concepts, original title, tests & measures, mesh] | 542721 |
| 13 | 9 and 10 and 11 and 12 | 184 |
| 14 | limit 13 to up=20190527-20211130 | 27 |

**Scopus Searched November 22, 2021 Results = 6 (of total of 48 before date limit)**

( ( TITLE-ABS-KEY ( ( habenaria OR habenaria OR algonquin* OR algonquian* OR anishinabe* OR anisate OR anishinaabe* OR asinine OR athapaskan OR beothuk* OR blackfoot OR chipewyan OR cree OR dgrip OR dene OR eskimo* OR esquimau* OR gwich'in OR adenosine OR huron ) ) ) OR ( TITLE-ABS-KEY ( inuvaluit* OR inuit* OR inn OR inoue OR incus OR ink OR iroquois OR montagnei OR mainsheet OR naskapi* OR micmac OR micmac OR migma OR milkman OR micmac OR migma OR migma OR mohawk OR ojibw* OR pashtu ) ) OR ( TITLE-ABS-KEY ( maroteaux OR maroteaux OR slavey OR tricho OR yellowknife ) ) OR ( TITLE-ABS-KEY ( "premiere nation" OR first AND nation OR first AND nations OR metis OR mischief OR litchi OR motif OR metorchis OR bois-brule* OR mixed-blood* OR half AND breed* OR halfbreed* ) ) OR ( TITLE-ABS-KEY ( urban AND indian* OR "one reserve" OR "off reserve" OR country AND food* OR shaman* ) ) OR ( TITLE-ABS-KEY ( ( ( ( native* OR indian OR indians ) W/2 ( person OR persons OR man OR woman OR men OR women OR child* OR youth OR youths OR population* OR people* OR band OR bands ) ) not AND plant* ) ) ) ) AND ( TITLE-ABS-KEY ( suicid* OR "self harm" ) ) AND ( TITLE-ABS-KEY ( communit* OR family OR families OR caregiver* OR gatekeeper* ) ) AND ( TITLE-ABS-KEY ( train OR trained OR training OR educat* OR know* OR awareness ) ) AND PUBYEAR > 2019

**Cochrane Library Searched May 27, 2019 Results = 5**

## 1 Review and 4 Trials matching suicid* or "self harm" in Title Abstract Keyword AND communit* or family or families or gatekeeper* or caregiver* in Title Abstract Keyword AND know* or awareness or train or training or trained or educat* in Title Abstract Keyword AND Indigenous* or Aboriginal* or Aboriginie or Maori or "Torres Strait Islander*" or Amerind* or Cree or Blackfoot or Chipewyan or Dene or Haudenosaunee or Huron or Mohawk or Anishinabe* or Inuvialuit or Assiniboine or Abenaki or Algonquin or Haida or Salish or Gwitchin or Athapaskan or Inuit* or Innu or Innue or Innus or Inuk or Metis or "Mixed-blood" or "Bois Brule" or "First Nation" or "First Nations" or Metchif or Meschif or Metif or autochtone* or Indidgenes or Eskimo or Esquimau* in Title Abstract Keyword NOT Africa or Iran or Rwanda* in Title Abstract Keyword - (Word variations have been searched)

**CINAHL Searched November 23, 2021**

| \| # \| Query \| Results \| \| --- \| --- \| --- \| \| S1 \| (MH "Indigenous Peoples+") or (MH "Health Services, Indigenous") OR (MH "Indigenous Health") or (MH "Aboriginal Australians") OR "Indigenous" OR (MH "Eskimos+") or (MH Maori") or (MH "Native Americans") \| 28,402 \| \| S2 \| (Abenaki or Abenakis or Algonquin* or Algonquian* or Anishinabe* or Anishnabeg or Anishinaabe* or Assiniboine or Athapaskan or Beothuk* or Blackfoot or Chipewyan or Cree or Dogrib or Dene or Eskimo* or Esquimau* or Gwich'in or Haudenosaunee or Huron) or (Inuvaluit* or Inuit* or Innu or Innue or Innus or Inuk or (Iroquois not (corn or homeobox or transcription)) or Montagnais or Maliseet or Naskapi* or Micmac or Mic-Mac or Mi-gmaq or Mi-kmaq or "Mic mac" or Migmaw or "Mig maw" or Mohawk or Ojibw* or Sahtu or Salteaux or Saulteaux or Slavey or Tlicho or Yellowknives or (Peuple N1 (autochtones or indidgenes or premier or racing or natif*)) or "Premiere Nation" or "First Nation" or "First Nations" or Metis or Mischif or Mitchif or Metif or Metchif or Bois-brule* or Mixed-blood* or "Half Breed*" or halfbreed* or (traditional N1 (medicine* or heal* or food* or health*)) or "Urban Indian*" or "on reserve" or "off reserve" or "country food*" or shaman* or "medicine man" or "medicine woman" or ((native* or Indian or Indians) N2 (person or persons or man or woman or men or women or child* or youth or youths or population* or people* or band or bands))) \| 49,324 \| \| S3 \| ("Native American*" or (Native N2 (Alaska* or Hawaiian*)) or "American Indian*" or Amerindian* or (Absaroke or "Alaskan Athabascans" or Aleut or A'aninin or Anishinaabe or Aniyunwiya or (Apache not "Apache II") or Arapaho or Arikara or Baxoje or Blackfeet or Bode'wadmi or Caddo or Cayuse or Chahta or Cherokee or Cheyenne or Chikasha or Chickasaw or Chippewa or Choctaw or Comanche or Cree or Cayuga or Dakelh or (Dine' not "Dine 1") or Eskimo* or Ewiiaapaayp or Gaigwu or Gayogohono or "Gros Ventre" or Havasupai or Hinonooeino or Haudenonsaunee or Hidatsa or Ho-Chunk or Hopi or Hualapai or Houma or Illiniwek or Illini or Iyiniwok or Ininiwok or (Iroquois not (homeobox or transcription)) or Kadohadacho or Kanienkehaka or Kanonsionni or Karok or Kickapoo or Kiowa or Kiwigapawa or Klickitat or Kumeyaay or Lanape or Lakota or Lumbee or Maidu or Maklak or Mamaceqtaw or Mandan or Maumee or Menominee or Meskwaki or Miccosukee or Mikasuki or Minisink or Mohawk or Mohegan or Mohican or Mohingan or Muheconneok or Munsee or Muskogee or Myaamia or Nakota or Nanigansek or Nantego or Narragansett or Navajo or Narragansett or Nde or Ndee or Niukonska or Numakiki or Numinu or Nunt'zi or Nuutsiu or Nuxbaaga or Odawa or Ojibway or Ojibwe or "Ohkay Owingeh" or Olekwo'l or Onandowaga or Oneida or Onondaga or Onundaga'ono or Onyota'aka or Osage or Pahoja or Panawahpskek or Passamaquoddy or Papago or "Pend d'Oreilles" or Penobscot or Peskotomuhkati or Piaute or Pima or Pokanoket or Pomo or Ponca or Potawatomi or Powhatan or Po-wo-ge-oweenge or Pueblo or "Puget Sound Salish" or Quapaw or Qwulhhwaipum or Sahnish or Sauk or Sekani or Seminole or Shawnee or Shawanwa or Schitsu'msh or Shoshonee or Shuyelpee or Siksika or Skarooren or (Souix not "Souix Falls") or Ta-o-ta or Tetawken or "Tete de Boule" or Teton or Thlingchadine or Tohano O'odham or Tonkawa or Tlingit-Haida or Tohono or Tsalagi or Tsitsistas or Tuf-shum-tia or Tus-Tah or Tuscarora or O'odham or Ugakhpa or Umon'hon or Ute or Wampanoag or Wendat or Wihyot Winnebago or Who-ge-owenge or Wyandot or Yakima or Yaqui or Yavapai or Yurok or Yuman)) \| 20,847 \| \| S4 \| (((Acatec or Aguactec or Amuzgo or Chatino or Chiapaneca or Chichimeca or Chicomuceltec or Chinantec* or Chocho or Chontal or Chuj or Cochimi or Cocopa or Cuicatec* or Guarijio or Haurijio or Huastec* or Huave or Huichol or Ixcatec* or Jacaltec* or Kanjobal or Kaqchikel or Kekchi or K'iche' or Kickapoo or Kikapu or Kiliwa or Kumiai or Lacandon or Matlatzinca or Mazahua or Mazatec* or Mixe or Mixtec* or Mocho or Motocintleco or Nahua* or Oaxaca or Ocuiltec or Opata or Otomi or Paipai or Pame or Papago or Pima Bajo or Popoloca or Popoluca or (Purepecha not Echeveria) or Raramuri or Tabasco Chontal or Tacaneco or Tacuate or Tarahumara or Tectiteco or Teenek or Tenek or Tepehua or Tepehuan or Tlahuica or Tlapanec or "Tohono O'odham" or Tojolabal or Toltec or Totonac or Trique or Triqui or Tsotsil or (Tubar not ("tubar pregnancy" or "tubar sterility")) or Tzeltal or Tzotzil or Wixarika or Yaqui or "Yucatec Maya" or Zapotec or Zoque) or ((Cora or Mame or Maya or Mayos or Mayo or Mam or Chol or Ch'ol or Ixil or Seri) N3 (Indian* or tribe* or tribal or people* or person or elder or youth or child or children or men or men or man or woman or women or adolescent)) or (((Mexico not "New Mexico") or Aguascalientes or Baja California or "Los Cabos" or "La Paz" or Loreto or Campeche or Chiapas or Chihuahua or Coahuila or Colima or Manzanillo or "Distrito Federal" or Durango or "Estado de Mexico" or Guanajuato or "Leon San Miguel de Allende" or Guerrero or Acapulco or Hidalgo or Jalisco or "Puerto Vallarta" or Guadalajara or Michoacan or Morelia or Morelos or Nayarit or "Nuevo Leon" or Oaxaca or Huatulco or "Puerto Escondido" or Puebla or Queretaro or "Quintana Roo" or Cancun or Cozumel or "San Luis Potosi" or Sinaloa or Sonora or Tabasco or Tamaulipas or Tlaxcala or Veracruz or Yucatan or Merida or Zacatecas or Zenacantan* or Zenacanteco*) N7 (("pre Columbian" N3 culture*) or (preColumbian N3 culture*) or Indian or Indians or Aboriginal* or Indigen* or "First Peoples" or "Native Mexican*" or "indos mexicano" or "pueblos indigenas" or shaman* or tribe or tribal or tribes or Amerindian* or traditional or Mesoamerindian*))) not ((Blinder N2 Oaxaca) or Guatemala or "Pepper Huasteco Virus" or "opata m85" or polar or cladoceran*).mp.) or (((MH "Indians, North American" or (("pre Columbian" N3 culture*) or (preColumbian N3 culture*) or Indian or Indians or Aboriginal* or Indigen* or "First Peoples" or "Native Mexican*" or "indos mexicano" or "pueblos indigenas" or shaman* or h'iloletik or curander* or curandeiro or yerberos or herbalist* or hueseros or parteras or oracionistas or tabaqueros or ayahausqueros or peyoteros or sobadores or espiritualistas or tribe or tribal or tribes or Amerindian* or traditional or Mesoamerindian*)) and ((MY "Mexico" or (Mexican or mexico or mexico's or mexicano or mexicali))) \| 1,578 \| \| S5 \| (Abipon or Achuar or Achuagua or Akawaio or Amarizana or Andoque or Akawaio or Akuriyo or Anauya or Araona or Arawak or Ayamn or Aguaruna or Amahuaca or Amarakaeri or Andoa or Arabela or Arawak or Arhuaco or Ashaninca or Asheninca or Atsahuaca or Aymara or Ayoreo or Bakairi or Baniva or Barasana or Baniwa or Baure or Bororo or Cabiyari or Cacataibo or Caquinte or Cacua or Cahuarano or Caiua or "Camara Indians" or Camaracoto or Camsa or Canamari or Candoshi or Canela or Canichana or Capanahua or Carapana or Cariay or Carib or Carijona or Carutana or Cashibo or Cashinahua or Cawishana or Cavinena or Caxuiana or Cayuvava or Chontaquiro or Cocama or Cubeo or Curipaco or Chacobo or Chaima or (Chana not striatus) or Chapacura or Charrua or Chimila or Chitonahua or Chorote or Chipaya or Chiquitano or Chulupi or Carare or Coconuco or Cofan or Coreguaje or Coyaima or Chamacoco or Chamicuro or Chayahuita or Cocama or Culina or Culino or Cubeo or Cuiba or Cuiva or Cumanagoto or Curripaco or Deni or Desano or Embera or Guarani or Guajajara or Guana or Guanano or Guarayo or Guarayu or Guahibo or Guajiro or Guambiano or Guanano or Guayabero or Guarequena or Guinao or Guana or Gayon or Guahibo or Hixkaryana or Huachipairi or Huambisa or Huarayo or Iauanaua or Ikpeng or Ingariko or Irantxe or Itonama or Inapari or Iquito or Isconahua or Jumana or Japreria or Jirajara or Juruti or Jaqaru or Jebero or Kadiweu or Kaingang or Kamayura or Karaja or Karipuna or Kariri or Katukina or Kaxarari or Kayabi or Kayapo or "Kuikuro alapalo" or Kulina or Kaiwa or Kallawaya or Kogui or Kuna or Kaweskar or Lule or Macuna or Maipure or Mapuche or Mataco or Mocovi or Machinere or Machinerev or Machiguenga or Macushi or Macuna or Madi or Malayo or Mamainde or Manao or Mandauaca or Mandawaka or Mapidian or Mapuche or Mapidian or Maquiritare or Maquiritari or Maragua or Marawan or Mariate or Marubo or Mastanahua or Matipuhy or Matis or Matses or Mawakua or Mawakwa or Maxakali or Mehinaku or Miranha or Moronawa or Munduruku or Mataco or Movima or Muellama or Muinane or Mapoyo or "Mashco Piro" or Matses or Muniche or Nambikwara or Nocaman or Nuquini or Nomatsiguenga or Nanti or Ocaina or Omagua or Orejon or Opon or Pacahuara or Paez or Paicone or Palicur or Panare or Pano or Paresi or Paumari or Pemon or Pilaga or Puelche or Pauna or Pauserna or Piapoco or Piraha or Piratapuyo or Pisabo or Piaroa or Pijao or Piratapuyo or Paraujano or Pemon or Pemono or Piapoco or Puinave or Patamona or Poyanawa or Puinave or Puquina or Quechua or Quichua or Retuara or Resigaro or Reyesano or Sabanes or Saliba or Saluma or Sarave or Secoya or Selknam or Sensi or Shaninawa or Shapra or Sharanahua or Shebayo or Shiwiar or Shikiana or Sikiana or Siriono or Sinsiga or Siona or Suruwaha or Tacano or Tamanaco or Tiahuanaco or Tariano or Tehuelche or Tariano or Tatuyo or Tembe or Terena or Telembi or Ticuna or Ticuna or Tiriyo or Tiwanaku or Tiwanaku or Torom or Totoro or Tsimane or Tuberao or Tucano or Tunebo or Tuxinawa or Tuyuca or Uainuma or Urarina or Vilela or Waimaha or Waiampi or Waiwai or Wapishana or Waraiku or Warekena or Waura or Wayampi or Wayana or Wirina or Waimaha or Waunana or Wiwa or Warao or Wayuu or Witoto or Xavante or Xipaya or Xiriana or Xokleng or Yabaana or Yaminawa or Yaminahua or Yaruma or Yawalapiti or Yuracare or Yabarana or Yavitero or Yine or Yamana or Yaghan or Yucuna or Yurumangui or Yukpa or Yanesha or Yoranahua or Yagua or Yaminahua or Zaparo or Zamuco or ((Inga or Maca or Leco or Mojo or Uro or Maco or Lengua or Toba or Zoe or Ona or Catio or Passe or Bari or Awa or Bora or Bara or Remo or Pano or Sape) N3 (Indians or Indian or Indigenous or Amerindian* or Aborigin* or people or peoples or women or men or woman or man or child* or youth or youths or baby or babies or tribe or tribes or tribal or shaman* or native or traditional)) or "Trio Indians" or "More Indians" or "Bare Indians") or ((Indian* or Amerindian, or Aboriginal* or indigenas or Indigenous) and (Argentin* or Bolivia* or Brazil* or Chile* or Colombia* or French Guiana* or Guyana* or Peru or Paraguay or Uruguay or Venezuela or Amazon or Amazons or Amazonia or Andes or Andean)) \| 15,300 \| \| S6 \| ((Queensland or New South Wales or NSW or Northern Territory or Canberra or (Sydney not (Canada or Scotland)) or ((Melbourne not (England or United Kingdom)) or Adelaide or Tasmania or (Perth not Scotland) or Austral*)) and ((Indigen* or Aborig* or tribe or tribal or tribes or traditional or remote or outback or Blackfella* or Aborigin* or Indigenous* or "first people*" or "original people") or Ngunnawal or Murrawarri or Alyawarre. or Anmatjera or Arrernte. or Gurindiji or Kunibidji or Luritja or Murrinh Patha or Pitjantjatjara or Tiwi or Waripiri or Yoingu or Guugu Yimithirr or Kalkadoon or Adnyamathanha or Adynyamathanha or Dieri or Kaurna or Maralinga Tjarutja or Ngarrindjeri or Narungga or Gunai or Kurnai or Kulin or "Yorta Yorta" or Bangerang or Kailtheban or Wollithiga or Moira or Ulupna or "Kwat Kwat" or "Yalaba Yalaba" or "Ngurai illiam wurrung" or Jarrakan or Noongar or Nyungar or Nyoongar or "Pila Iguru")) or "Torres Strait Islander*" \| 14,423 \| \| S7 \| (Saami or Sampi or (Sami not Ulus) or Samis or Southernsami* or Umesami* or Pitesami* or Lulesami* or Northernsami* or Enaresami* or Kolasami* or Lapp or Lapps or Lappish or Lappland or (Lapland* not longspur) or Lappalainen* or Saamelainen* or "reindeer herd*" or "reindeer culture*" or "reindeer pastoral*" or Lappbys or Samebys or reinbeitesdistrikt or paliskunta or siida) or (((Fennoscandia or Finnmark or Scandinavia or Nordic or Sweden or Norway or Finland or Swedish or Finnish or Norwegian or Norge or Svensk* or Suomi or Barents Region or (Kola not (garcinia or gotu)) or "Arctic Europe*" or "Polar Europe*" or "North* Europ*") or (MH "Finland") or (MH "Norway") or (MH Sweden/) and ((traditional N3 (food* or heal* or medicine* or shaman*)) or (Indigen* N3 (people* or person* or mother* or father* or parent* or child* or boy or boys or girl* or youth* or healer* or patient* or famil* or herder*))) \| 83,624 \| \| S8 \| s1 or s2 or s3 or s4 or s5 or s6 or s7 \| 185,139 \| \| S9 \| (MH "Suicide") or (MH "Suicide, Attempted") \| 26,399 \| \| S10 \| (MH "Suicidal Ideation") \| 8,893 \| \| S11 \| suicid* or "self-harm" \| 48,161 \| \| S12 \| s9 or s10 or s11 \| 48,161 \| \| S13 \| ( (communit* or caregiver* or family or families or gatekeeper*) ) AND ( traini or trained or training or educat* or know* or awareness ) \| 239,007 \| \| S14 \| (( (communit* or caregiver* or family or families or gatekeeper*) ) AND ( traini or trained or training or educat* or know* or awareness )) AND (S8 AND S12 AND S13) \| 200 \| \| S15 \| s14 NOT ( Africa or Iran or Malawi or Mallee ) \| 198 \| \| S16 \| s14 NOT ( Africa or Iran or Malawi or Mallee ) Published Date: 20190501-20211231 \| 37 \| |
| --- | --- | --- | --- | --- | --- | --- | --- | --- | --- | --- | --- | --- | --- | --- | --- | --- | --- | --- | --- | --- | --- | --- | --- | --- | --- | --- | --- | --- | --- | --- | --- | --- | --- | --- | --- | --- | --- | --- | --- | --- | --- | --- | --- | --- | --- | --- | --- | --- | --- | --- | --- |

**PROSPERO Searched November 23, 2021**

**Line Search for Hits**

#1 Indigenous* or Aboriginal* or Aboriginie or Maori or "Torres Strait Islander*"
 or Amerind* or Cree or Blackfoot or Chipewyan or Dene or Haudenosaunee or
Huron or Mohawk or Anishinabe* or Inuvialuit or Assiniboine or Abenaki or Algonquin
 or Haida or Salish or Gwitchin or Athapaskan or Inuit* or Innu or Innue or Innus or
 Inuk or Metis or "Mixed Blood" or "Bois Brule" or "First Nation" or "First Nations"
 or Metchif or Meschif or Metif or autochtone* or Indidgenes or Eskimo or Esquimau* 744

#2 Communit* or family or families or gatekeeper or caregiver* 24090

#3 know* or awareness or train or training or trained or educat* 78850

#4 suicid* or "self harm" WHERE CD FROM 01/05/2019 TO 31/12/2021 1571

#5 #1 AND #2 AND #3 AND #5 13

**SocIndex** **Searched May 27, 2019**

| EBSCO SocIndex Searched November 22, 2021  Limiters - Date of Publication: 20190501-20211231  Search modes - Find all my search terms   \| **#** \| **Query** \| **Results** \| \| --- \| --- \| --- \| \| S1 \| ( ( ((((((DE "INDIGENOUS peoples" OR DE "INDIGENOUS children" OR DE "INDIGENOUS youth") AND (DE "INDIGENOUS peoples of the Americas" OR DE "NATIVE Americans" OR DE "NATIVE Americans")) OR (DE "MAORI (New Zealand people) -- Ethnic identity")) OR (DE "FIRST Nations")) OR (DE "ESKIMOS")) OR (DE "ARCTIC peoples")) OR (DE "METIS") OR (Abenaki or Abenakis or Algonquin* or Algonquian* or Anishinabe* or Anishnabeg or Anishinaabe* or Assiniboine or Athapaskan or Beothuk* or Blackfoot or Chipewyan or Cree or Dogrib or Dene or Eskimo* or Esquimau* or Gwich'in or Haudenosaunee or Huron) or exp Inuits/ or (Inuvaluit* or Inuit* or Innu or Innue or Innus or Inuk or (Iroquois not (corn or homeobox or transcription)) or Montagnais or Maliseet or Naskapi* or Micmac or Mi gmaq or Mi kmaq or Mic mac or Migmaw or Mig maw or Mohawk or Ojibw* or Sahtu or Salteaux or Saulteaux or Slavey or Tlicho or Yellowknives or (Peuple adj (autochtones or indidgenes or premier or racing or natif*)) or "Premiere Nation" or First Nation or First Nations or Metis or Mischif or Mitchif or Metif or Metchif or Bois-brule* or Mixed-blood* or Half Breed* or halfbreed* or (traditional N1 (medicine* or heal* or food* or health*)) or Urban Indian* or "on reserve" or "off reserve" or country food* or shaman* or medicine man or medicine woman or ((native* or Indian or Indians) N2 (person or persons or man or woman or men or women or child* or youth or youths or population* or people* or band or bands)) ) AND ( ((DE "SUICIDE" OR DE "ASSISTED suicide" OR DE "COPYCAT suicide" OR DE "MASS suicide" OR DE "RATIONAL suicide" OR DE "SELF-immolation" OR DE "SUICIDE bombings" OR DE "SUICIDE pacts" OR DE "SUICIDAL behavior" OR DE "SUICIDAL ideation" OR DE "SUICIDE pacts" OR DE "SUICIDE -- Cross-cultural studies" OR DE "SUICIDE prevention") or suicide*) ) AND ( training or trained or train or educat* or know* or awareness ) AND ( community or family or families or caregiver* or gatekeep* ) NOT ( africa or Mallee or Egypt or malawi or zimbabwe ) AND ( ( training or trained or train or educat* or know* or awareness ) ) AND ( ( community or family or families or caregiver* or gatekeep* ) ) NOT ( africa or Mallee or Egypt or malawi or zimbabwe ) \| 60 \| |
| --- | --- | --- | --- | --- | --- | --- |

**Proquest Dissertations and These Global Searched November 23, 2021
Results =11**

noft(indigenous OR "American indian" OR aboriginal OR Metis OR inuit OR "first nation" OR "first nations" or cree or blackfoot or Chipewyan or Dogrib or Dene or inuvialuit or montagnais or maliseet or naskapi* or micmac or migmaw or mohawk or ojibw* or sahtu or salteaux or saulteaux or slavey or tlicho or yellowknives or Gwich'in or Hudenosaunee or Huron or Iroquois or "Alaska native*" or "torres strait islander*" or maori or aboriginie* or saami or "first peoples" ) AND noft(suicid* OR "self harm") AND noft(train OR trained OR training OR educat* OR awareness OR know*) AND noft(communit* OR family OR families OR caregiver* OR gatekeeper*)
