# Supplementary material for: Centering Indigenous knowledge in suicide prevention: a critical scoping review
Source: BMC Public Health. 2022 Dec 19;22:2377. doi: 10.1186/s12889-022-14580-0 (PMC9761945; doi:10.1186/s12889-022-14580-0)
Supplement: Supplementary file 2 — Additional file 2: Appendix B. Academic literature data extraction table [123, 124]. [file 12889_2022_14580_MOESM2_ESM.docx]

**APPENDIX B: Academic literature data extraction table**

| Author(s) | Type of paper | Country | Indigenous population | Name of intervention | Type of intervention | Indigenous understandings of suicide | Processes for community engagement and cultural inclusion^1,2^ | Main Outcomes |
| --- | --- | --- | --- | --- | --- | --- | --- | --- |
| Allen, 2009 [39] | Primary research, Peer-reviewed  Non-randomized study design  n=54 | AK, USA | Alaskan Native | The People Awakening Project | Community prevention: “Cultural prevention program” | Not discussed/defined | - CBPR - Local community planning group and university researchers involved in development of program. - Key informants involved in development of Community Readiness Assessment tool (CRA) - Cultural intervention | - Community protective factors & readiness increased - Community moved from stage of resistance to preparation/readiness to address suicide; leadership readiness moved to the initiation stage (program implementation) |
| Allen, 2014 [73] | Descriptive paper, Peer-Reviewed | AK, USA | Alaskan Native | The People Awakening Project | Community prevention: “Strengths-based, multi-level community intervention” | Locally developed theory of protective factors from suicide and alcohol abuse includes community (*Nunamta*), family (*Ilaput*), individual (*Yuum ayuqucia*) characteristics; social environment (*Nunaput*); reflective processes (*Umyuancaryaraq*); and reasons for life (*Yuuyaraqegtaar*) | - CBPR - Local and statewide Indigenous groups involved in developing underlying theory, cultural framework and intervention activities | - Generation of a robust, locally developed theory of protective factors from suicide and alcohol abuse - Presents justification for multilevel interventions that simultaneously address community, family, and individual levels rather than interventions directed at any single level |
| Allen, 2018 [39] | Primary research, Peer-reviewed  Non-randomized control study design  Treatment arm n=54  Control arm n=74 | AK, USA | Alaskan Native | Qungasvik | Community prevention: “Multi-level Culturally-grounded intervention” | Not discussed/defined | - CBPR - Involved Indigenous Elders, experts and community leaders - Cultural intervention | - Direct intervention effects on proximal or intermediate variables of protective factors at the individual, family, community, and peer influences levels lead to later change on the ultimate prevention outcome variables of Reasons for Life protective from suicide risk, and Reflective Processes about alcohol use consequences protective from alcohol risk. Mixed effects regression models contrasted treatment and comparison arms, and identified significant intervention effects on Reasons for Life (d = 0.27, p < .05) but not Reflective Processes. |
| Anang, 2019 [45] | Primary research, Peer-reviewed  Qualitative via focus groups  n=36 | Canada | Inuit | Building on Strengths in Naujaat | Community prevention: “Youth resiliency project” | - Focusing on resiliency resonated strongly, Elder stated “Why do we talk about suicide all the time!? Let’s talk about love!” (pg. 3) - Shift of focus from deficits and problems leading to suicide to community strengths and assets, life promotion. | - CBPR - Community Health Council, Elders, Hamlet Council and youth involved throughout | - Major themes: importance of long-term relationships built on mutual respect and trust, the minimisation of the “us” and “them” divide, and the use of stories, open communication and listening as pathways to transcultural understanding - Youth ownership in the design and implementation helped to generate knowledge and collective visions and agency for a better future - Potential to enhance community uptake & sustainability and thus contribute to greater resilience & self-determination of youth |
| Antonio, 2020 [84] | Primary research, Peer-reviewed  Qualitative via semi-structured interviews  n=26 | Hawaii, USA | Hawaii Native | The Hawai‘i’s Caring Communities Initiative (HCCI) for Youth Suicide Prevention | Community prevention: “Strength-based, youth- leadership approach to suicide prevention” | Disparities in suicide among Native Hawaiians “may be attributed to historical trauma and structural inequalities stemming from colonization. Despite these disparities, strengths persist in Native Hawaiian communities.” (p. 96) | - Partnership between University of Hawai‘i Department of Psychiatry, the Prevent Suicide Hawai‘i Task Force, and 6 rurally-based organizations that serve Native Hawaiian and Other Pacific Islander youth - Community coordinators and youth leaders certified as Connect trainers - “Culturally-tailored” | - Five themes emerged that fit with an adapted socio-ecological model framework, which included increased knowledge in suicide risk, pride in leadership identity, sense of positive relationships, positive affirmation from community members, and sustainability. |
| Armstrong, 2018 [46] | Primary research in a peer-reviewed journal  Qualitative via Delphi consensus method  n=27 | Australia | Australian Aboriginal and Torres Strait Islander | N/A | Gatekeeper training: “Cultural adaption of a Mental Health First Aid course” | Suicide “is a complex socio-cultural, political, biological and psychological phenomenon that needs to be understood in the context of colonisation, loss of land and culture, transgenerational trauma, grief and loss, and racism and discrimination." (pg. 2) | - Indigenous experts in suicide prevention asked to evaluate components of training but not engaged in development - 3 authors are Aboriginal and Torres Strait Islanders - Cultural adaption | - From total of 301 statements shown to the expert panel, 172 were endorsed as helping statements to be included in re-developed guidelines |
| Armstrong, 2020 [47] | Primary research, peer-reviewed  Pre/post survey design  n=192 | Australia | Aboriginal and Torres Strait Islander people | Talking About Suicide | Gatekeeper training: Evaluation of a “cultural adaption of a suicide gatekeeper training course” | Course acknowledges that "the high rates of suicide in Aboriginal and Torres Strait Islander people today stem from the disrupting effects and systemic harms caused by colonisation and its aftermath, including loss of land and culture, trans-generational trauma, racism and discrimination." (pg. 4) | - Course guide developed by local Aboriginal experts - Authors engaged Aboriginal and Torres Strait Islander Project Reference Group for cultural guidance and support throughout study and in developing course - “Culturally-appropriate intervention”/ cultural adaption | - Course improved participants’ attitudes and intended and actual assisting behaviours - Assisting actions recommended during the training were implemented by many participants in the 4 months since participating in course, demonstrating immediate benefits for Aboriginal and Torres Strait Islander people experiencing suicidal thoughts |
| Asher BlackDeer, 2020 [81] | Review paper,  Evidence Mapping, Peer-reviewed | USA | American Indian/Alaska Native | N/A | Review: Evidence map of culturally specific mental health interventions for American Indian youth | Suicide within Indigenous populations needs to be understood within the larger context of historical and ongoing trauma and “American Indian and Alaska Natives (AI/AN) have a longstanding history of colonialism, which has had far-reaching impacts on culture and spirituality.” (p. 50). Contrasted with “assumptions embedded in the Western medical model which emphasizes distress and dysfunction.” (p. 50) | Details not provided | - 9 interventions were mapped as research-supported interventions for American Indian mental health. - Interventions fell into one or more of four main categories: school-based services, cultural adaptions, culture as treatment, and community involvement. |
| Barnett, 2020 [36] | Primary research, Peer-reviewed  Pre/post survey design  n=111 | USA | Alaska Native youth | Culture Camps | Community prevention: Pilot evaluation of “Culture camps as a health promotion intervention” | Highlights relations between colonialism, compounded trauma and suicide in Indigenous populations. States, "higher rates of suicide in tribal communities are linked to a lack of cultural continuity." (pg. 365) | Details not provided | - Pilot evaluation of the culture camps uses a quantitative pre/post design to examine the outcomes of self-esteem, emotional states, belongingness, mattering to others, and coping skills among participants. - Results indicate culture camps can significantly increase positive mood, feelings of belongingness, and perceived coping of participants. |
| Caldwell, 2008 [85] | Descriptive paper, Peer-reviewed | Canada | 2 Indigenous communities in Atlantic Canada | The Suicide Prevention Continuum/ Aboriginal Community Youth Resilience Network | Community prevention: “Research framework for supporting Aboriginal  community-led suicide prevention with academic technical resources” | - “Suicide can adversely affect the well being of entire communities — a public health dimension probably even more important in close-knit and often isolated Aboriginal communities…. This requires a much stronger community…role in suicide prevention, not just as implementers of externally motivated programs, but in the conceptualization and design of prevention initiatives that build systematically on Aboriginal strengths.” (p. 1) - “The idea was to look at suicide risk as something that might be eliminated or reduced before it ever became a threat, protecting youth from suicide before they ever reached the point of making an attempt.” (p. 1) | - With technical assistance, 2 Indigenous communities in Atlantic Canada explored adolescent suicide and community-guided approaches to suicide prevention - First step was to document the differences between those who were resilient to suicide and those who were at risk - With this inventory of resilience characteristics, communities formulated their own interventions to strengthen these traits and conditions - Discussions with working groups from the communities led to a questionnaire to look at youth wellness - Working groups talked about issues facing their youth and, based on this, developed an adolescent survey to assess factors related to suicide risk - Project evolved into Aboriginal Community Youth Resilience Network | - Suicide Prevention Continuum was developed - Classifies prevention into primary, secondary, and tertiary interventions: primary preventions focus on building/strengthening protective factors in youth increasing their resiliency to suicide; secondary prevention (also called intervention) involves intervening early when risk factors for suicide have emerged or are emerging to prevent the onset of suicide related behaviour; tertiary preventions (also called postvention) are designed for those already displaying suicide related behaviours |
| Capp, 2001 [52] | Primary research, Peer-reviewed  Qualitative study design  n=48 Indigenous  n=9 non-Indigenous | Australia | Aboriginal and Torres Strait Islanders in New South Wales | N/A | Gatekeeper training | Not discussed/defined | - Authors consulted with communities and youth to identify culturally appropriate ways to develop prevention strategies, outcomes of which informed development of series of community gatekeeper training workshops - Language and cultural appropriateness was assessed by the Aboriginal members of the project team, followed by the community Aboriginal Mental Health Advisory Group - Not clear if communities were involved in the development or implementation | - Increase in participants’ knowledge about suicide, greater confidence in identification of people who are suicidal, and high levels of intentions to provide help. - Attitudes, subjective norms and barriers predicted intentions to help |
| Chung-Duo, 2016 [48] | Primary research, Peer-reviewed  Qualitative via focus groups  n=33 | Hawaii, USA | Hawaii Native | National Alliance on  Mental Illness-New Hampshire’s (NAMI-NH) Connect Suicide Prevention Program | Community prevention AND Gatekeeper training: Cultural analysis of NAMI-NH Connect Suicide Prevention Program | Disparity in suicide among Native Hawaiians “may be attributed to multiple risk factors including the intergenerational effects of historical trauma and cultural loss, which may be perpetuated by the use of [evidence-based programs] uninformed by cultural values, beliefs, practices, and epistemology.” (p. 2) | - Initial focus group conducted with community leaders to inform a cultural adaption and implementation of the Connect Suicide Prevention Program in Hawai‘i - Involved local suicide prevention experts and community leaders in the study and created a framework that can be used to culturally adapt evidence-based programs - Level of community engagement in design, implementation and evaluation processes unclear | - 4 major themes of cultural needs were identiﬁed by community leaders, which emphasized the importance of honoring community knowledge and prioritizing relationships. - Findings were used to inform a programmatic cultural framework that can guide those who wish to implement or culturally adapt evidence-based programs with minority communities. |
| Cox, 2014 [62] | Primary research, Peer-reviewed  Qualitative via community engagement workshop  n=unclear | Australia | Aboriginal and Torres Strait Islanders | Cultural, Social and Emotional Well-being (CSEWB) Program | Community prevention: “Cultural, Social and Emotional Well-being (CSEWB) Program that aims to  promote the cultural, social and emotional well-being of  individuals and communities” | - “Social and emotional well-being problems in Aboriginal and Torres Strait Islander communities are attributed to factors including disadvantage and social, historic and political determinants that contribute to suicide, mental health problems and chronic disease.” (p. 346) - Focus to “promote social and emotional well-being and build resilience” (p. 345) | - National Advisory Committee consisting of experts and leaders in social emotional well-being developed relationships with Aboriginal organizations in communities - 2 co-researchers selected from each community - Individual, family, community contributors to suicide identified via consultations and workshops with community members & local organizations | - Individuals: restore & strengthen connections to culture, family & community; focus on youth; focus on health; offer life skills programs - Families: restore & strengthen connections within & between families through shared activities (to restore a sense of community); offer life skills programs; & provide access to education and/or training, as well as transport - Communities: focus on youth; restore & strengthen a sense of community through shared activities; self-determination; men’s and women’s groups; and provide access to employment, education, housing and transport |
| Cwik, 2016 [49] | Primary research, Peer-reviewed  Pre/post study design  n=13 | AZ, USA | American Indians residing on Fort Apache Indian Reservation | New Hope | Education/ awareness: Cultural adaption of Rotheram-Borus intervention | Not discussed/defined | - Community-driven participatory approach included involvement of “key stakeholders” throughout - Community Advisory Board (CAB) included: tribal leaders, study staff, representatives from tribal agencies, ED providers, and Elders - Cultural adaption | - Reductions in adolescents 'negative thinking, depression, and suicidal ideation, and an increase in psychological service utilization. - Key innovations include delivery by American Indian paraprofessionals and potential to strengthen the continuum of care between emergency department and outpatient settings |
| Cwik, 2016b [79] | Primary research, Peer-reviewed  Pre/post study design  n=75 Indigenous  n=8 non-Indigenous | AZ, USA | American Indian (Fort Apache Reservation) | ASIST | Gatekeeper training: “community-based cross-sectional pre-post  evaluation of ASIST” | “Barriers to care , lack of attention to historical trauma and power dynamics, and cross-cultural issues specific to suicide prevention can result in: (a) a mismatch between community needs and values with services, (b) the low use of mental health counseling, and (c) further compounding of existing risks.” (p. 403) | - Authors state that the work was done in partnership with the tribe, but no further information | - Significant increases in knowledge (p<0.001) and self-efficacy (p<0.001) were observed post-test - High satisfaction (3.53=5) and intent to use skills daily 36.4%) or monthly (66.3%). - Lowest rated was how the training addressed cultural differences (2.93). - Raises concerns about using standardized gatekeeper trainings “off the shelf” - Some potential areas of mismatch: participants were significantly less likely to endorse that helping requires professional skills, creating implications for intention to refer to mental health - Typical referral mechanisms are promoted by ASIST may be inconsistent with attitudes and access barriers |
| Cwik, 2019 [60] | Community-based participatory approach (CBPA), Peer reviewed  Qualitative interviews  n=8 | AZ, USA | American Indian (Fort Apache Reservation) | Nowhinalze’dayuweh bee goldoh dolee' and translated in English as “Let our Apache Heritage and Culture Live on Forever and Teach the Young Ones” | Community prevention: “Culturally-based, Elders prevention curriculum using a ground-up approach” | - “Our own research…as well as other leading researchers has highlighted culture as a protective factor for suicide…. Community stakeholders have also emphasized specifically generational rifts between Elders and younger generations as a risk factor with implications for culture as prevention.” (p. 138) - “[G]round up” prevention intervention focused on cultural strengths and resiliency.” (p. 143) | - CBPR - Tribal partners decided on intervention according to local risk, protective factors, and service gaps - Elders integral part of the intervention and Elder involvement was sustained even after grant funding ended - Elders initiated the idea to create curriculum and were fully engaged in design, development and implementation process | - Having local, Apache Celebrating Life (CL) suicide prevention staff to support the Elders was crucial - The ground-up process was very intensive & took 4½ years. This time was important to allow the Elders comfortability in generating topics and words to include in the curriculum, agree on content, fully engage in refinement process & make the curriculum their own - Ideal for an Elder or other local expert as curriculum writer, particularly when it comes to native language expertise - Ideal if local teachers are involved in the development and training of Elders, all preferably language speakers |
| Davies, 2020 [51] | Primary research, Peer-reviewed  Mixed-methods: surveys, in-depth interviews & workshop  observations  n=varied by data collection method | Australia | Aboriginal people in rural New South Wales | We Yarn training | Gatekeeper training: culturally safe suicide prevention skills training for  Aboriginal people and for those who work with Aboriginal communities and persons | Suicide knowledge is not defined; however, the authors state, "a culturally appropriate mental health framework must consider social, emotional and spiritual elements, as well as whole-of-community approaches." (pg. 580) | - Content developed in collaboration with local Aboriginal stakeholders and with guidance from Elders - Cultural intervention | - Significant improvements in understanding the links between cultural strengths, social and emotional wellbeing and suicide prevention - However, health professionals with existing knowledge wanted a stronger focus on clinical training |
| Fanian, 2015 [41] | Primary Research, Per-Reviewed  Mixed-methods: observational field notes, focus groups, questionnaires,  & reflective practice  n=varied by data collection method | NWT, Canada | Thcho in Behchoko, NWT | Ko` ts’iı`htła, means “We light the fire” | Community prevention: Arts-based suicide prevention program for youth focused on empowerment and building resiliency | - Recognitions of creative arts as effective health promotion tools to empower, engage and improve the health and well-being in Indigenous youth communities. - Focus on strengthening resiliency through culturally meaningful ways. | - Community-based and youth-led project - Project conceptualized during a suicide prevention workshop - Engagement of existing Community Action Research Team (CART) to build research capacity of the project - Cultural intervention | - Youth reported gaining confidence and new skills, both artistic and personal - Many youth found the workshop to be engaging, enjoyable and culturally relevant - Youth expressed an interest in continuing their involvement with the arts and spreading their messages through art to other youth and others in their communities |
| Fraser, 2020 [88] | Primary research, Peer-reviewed  Qualitative via workshops  n=unclear | Canada | Inuit in Nunavut and Nunavik | N/A | Community prevention: CBPR on suicide prevention | Suicide knowledge is not explicitly defined, however, the authors indicate "there is a direct link between the increase in suicide rates in Northern communities and the cumulative transgenerational effects of the historical processes of colonisation, and social marginalisation." (pg. 1) | - Engagement process guided by implementation research methods - Partnership with community and key local Inuit stakeholders, engagement occurred via subgroups organized according to priorities of action plan and workshops - Project did not complete objective; community partners were critical of the implementation science approach taken by the researchers | - Importance of self- governance and supporting community capacity so implementation and research can be designed and led by community and local/regional agencies - 3 identified priorities: (1) focusing on community mobilisation; (2) supporting access to scientific information; and (3) supporting the adaption of evaluation criteria and protocols of ongoing community activities |
| Goebert, 2018 [90] | Descriptive paper, Peer-reviewed | Hawaii, USA | Hawaii Natives | Hawaii’s Caring Communities Initiative (HCCI) for Youth Suicide Prevention & The Hawai‘i Youth Leadership Council for Suicide Prevention | Community prevention: “youth empowerment and leadership program” | - “Native Hawaiian values are at the forefront of our suicide prevention work, providing a deeper meaning and process.” (p. 334) - Four Native Hawaiian values as guiding principles: *aloha* (to love unconditionally), *ola* (life), *mālama* (to take care of, tend, attend, care for and show reverence) and *pilina* (connectivity and relationships)*.* | - Descriptive paper discussing how Hawaii cultural values can be understood and applied towards suicide prevention - Emphasis on how specific cultural notions/values as vital in suicide prevention efforts - Values allow youth to have experiential learning opportunities that integrate their thoughts and feelings | - Native Hawaiian values can build resilience among youth, building upon the strengths of Native Hawaiian youth and their respective communities - Contemporary approaches underpinned by ancestral traditions that resonate and thereby strengthen the resolve of youth to wait out with hope, defying thoughts of suicide - Through indigenization, involved communities can evoke history and tradition to develop culturally relevant, locally directed healing interventions - Suicide prevention and intervention efforts for Native Hawaiian communities must be culturally guided |
| Goodkind, 2010 [78] | Primary research, peer-reviewed  Non-randomized, one-group longitudinal study design  n=24 | NM, USA | 3 Southwest New Mexico Tribes (students) | Cultural adaptation to the Cognitive Behavioral Intervention for Trauma in Schools | Education/ awareness: “Cultural adaptation to the Cognitive Behavioral Intervention for Trauma in Schools” | Not discussed/defined | - Researcher obtained approval from Tribal or Chapter Councils and the School Boards in 3 communities, school administrators and teachers at each of the schools, and one tribal institutional review board. - This involved numerous community presentations and written documents from which they received support and feedback from tribal leaders, school board members, service providers, and community members that addressing trauma and violence exposure were priorities - Researchers shared revised curriculum and results of the study with tribal officials, school personnel, and other community members - Cultural adaption | - Participants experienced significant decreases in anxiety and PTSD symptoms, and avoidant coping strategies, as well as a marginally significant decrease in depression symptoms - Improvements in anxiety and depression were maintained 6 months post-intervention; improvements in PTSD and avoidant coping strategies were not - Concerns exist about the acceptability, feasibility, and appropriateness of intervention for American Indian communities, namely the screening process |
| Hamilton, 2010 [70] | Literature review, Peer-reviewed. | USA | American Indians | Numerous interventions | Review: Numerous interventions | - Emphasis of impact of suicide beyond individual/family to entire community and ensuring culturally competent approaches. - “Social workers need to be willing to take both a macro and a clinical stance on suicide prevention.” (p. 286) | Details not provided | - Importance of incorporating culturally specific programs and addressing suicide at a community level in an attempt to enhance the well-being of at-risk American Indian Adolescents |
| Harder, 2015 [67] | Primary research, peer-reviewed  Mixed-methods: pre/post survey & qualitative interviews  n=130 | BC, Canada | First Nations (Carrier Sekani of Northern BC) | Suicide prevention manual: “Nges Siy (I love you)” | Community prevention: Community-based interventions in youth suicide prevention | - Goal of project was to empower the communities to define suicide through their own lens; however this was not included in paper. - Authors state that they were “committed to placing Indigenous epistemology and ontology at the core of [their] work” (pg. 23) | - Authors are either Indigenous scholars or scholars who reside at the territories and with strong ties to the communities - A Leadership Advisory Council (LAC) founded to guide research and intervention development; consisted of Elders and youth whom represented 11 communities involved - Community members such as representatives of Elders and youth were involved in all steps of project - Cultural intervention | - Culturally appropriate and specific interventions can have an impact on reducing adolescent Aboriginal suicide via cultural awareness, connection, and identity; self-awareness, enhancement, and discovery; and attitudinal, behavioural, and developmental changes - By fostering good mental health and wellness, including pride in who one is and where one is from, leading indicators of suicide risk can be influenced in such a way that the person affected is less likely to make an attempt at suicide - Interventions must be designed uniquely for each community, and a pan-Indigenous approach must not be used |
| Harlow, 2014 [117] | Review, Peer-reviewed | Australia, Canada, New Zealand, and the United States. | Indigenous youth in Australia, Canada, New Zealand, and the United States. | N/A | Review: N/A | N/A | - Details not provided | - The search yielded 229 articles; 90 abstracts were assessed, and 11 articles describing nine programs were reviewed. - 2 Australian programs and seven American programs were included. - Programs were culturally tailored, flexible, and incorporated multiple-levels of prevention. - No randomized controlled trials were found, and many programs employed ad hoc evaluations, poor program description, and no process evaluation. |
| Holliday, 2018 [68] | Primary research, Peer-reviewed  Mixed-methods: Photovoice  (n = 16), digital storytelling (n = 4), & community capacity surveys (n = 128) | AK, USA | Alaska Native | Gathering  of Native American’s curriculum | Community prevention: Using CBPR to develop a pilot project to prevent suicide and substance use | - Important to start suicide prevention research with cultural assessment. They state that the first step to addressing suicide prevention is to ask the community “what is happening?” - “Culture-centered understanding of the issues will then be used in conjunction with the other community assessment data to plan and implement decision-making processes aimed at community-level policy change.” (p. 68) | - CBPR - Project led by a community advisory board (CAB) which was approved by the Tribal Council - Employed a mixed-methods community assessment to determine a relevant and culturally-centered community-wide suicide and substance use prevention intervention | - Quantitative and qualitative findings supported the need to (1) address youth suicide and substance abuse on the reservation and (2) use pre-existing resources available in the community - Results led to the development of a strengths-based intervention incorporating the Gathering of Native American’s curriculum |
| Isaac, 2010 [89] | Primary research, Peer-reviewed  Qualitative interviews  n=136 | Canada | 8 northwestern Manitoba First Nations | The Swampy Cree Suicide Prevention Team and Research Project | Community prevention | - “Established risk factors for suicide in the global Aboriginal population are community alienation, substance abuse, psychiatric problems, physical abuse, disconnection with cultural history, childhood separation and loss, lack of interpersonal support, lack of social capital and community participation and trust, exposure to suicide attempts or completions, cultural stress and a weakening of belief systems and spirituality.” (p. 259) | - Participatory action research - Collaborative partnership between researchers and members of Cree Nation Tribal Health - Critical to the establishment of the was a long-standing pre-existing relationship - Project was approved by the Swampy Cree Tribal Council Board of Directors, the Chief and Council of each of the communities as well as Cree Nation Tribal Health - Advisory councils developed in each community to lead the development and implementation of suicide prevention strategies - Initial stage was to have Indigenous knowledge guide understanding of suicidal behaviour | - Process evaluation - Key challenges: communities are over researched, local staff migration, recruitment issues and resistance to participation, jurisdictional challenges, difficulties accessing remote communities, interviewers felt despair and helplessness when confronted with participant life circumstances and history - Lessons learned: suitability of sharing circle format vs. focus group, importance of community-driven knowledge translation, sharing of emotional and personal information in focus group sessions sometimes necessitates individual interviews |
| Jacono, 2008 [61] | Descriptive paper, Peer-reviewed | Canada | Mi'Kmaq youth | Puppetry for Health  Promotion and Suicide Prevention | Community prevention | “While many factors contribute to suicide plans, the literature  would suggest “discontinuity” with heritage to have a particularly negative impact that needs priority redress.” (p. 50) | - Utilized a *two-eyed seeing approach*: blending of Indigenous knowledge with Western Science…”the aim of which is to examine the universe we live in from different but equal perspectives to come up with solutions.” (p. 52) - Partnership with the Integrative Science Program at Cape Breton University (a unique program designed for aboriginal youth), a culturally and educationally varied interdisciplinary team worked hand in hand with a group of esteemed tribal elders from Eskasoni First Nations on Cape Breton Island to produce several initiatives, the goal of which was the development of strategies to assist in the amelioration of mental health problems found in Mi’Kmaq First Nation communities in the Cape Breton Region - Cultural intervention | - Health promotion and suicide intervention involved using puppets made from natural forest materials to promote culture, language, and history and reduce “discontinuity” - Building continuity theorized to happen via puppetry performances by fostering connection to the land (claiming stewardship and knowledge of traditional hunting/gathering lands/learning the traditional names of gathered materials and their traditional functional or ritualistic use); bringing puppet makers into close contact with respected elders (reattachment of the young to their elders, the traditional holders of knowledge); emphasizing brotherhood, the need to look out for the welfare of others, especially when these others are unseen or out of sight; the need to share resources and to guard and preserve the life-giving properties of the resource - No formal evaluation conducted thus authors have ”no research evidence to support” (p. 53) |
| Jansen, 2021 [50] | Primary research, Peer-reviewed  Qualitative: focus groups  n=189 | USA | Alaska Native and American Indian (AN/AI) | Culturally adaption of 'Caring Contacts' | Community prevention: “Cultural adaption of suicide prevention intervention focused on increasing social connection” | “Community involvement is important when conducting research in AI/AN communities for two reasons: (1) research designed with and for AI/AN communities maximizes local strengths and resilience and (2) supporting AI/AN communities in self-determination resists the adverse impact of colonialism on AI/AN communities” (p. 3) | - CBPR - A Community Advisory Board (CAB) comprising two AI/AN people from each site guided study development and implementation by providing feedback on all aspects of the study - Focus group participants provided several suggestions on how to improve the cultural aspects of the participant experience - Cultural adaption | - 189 community members and other health system stakeholders in four tribal communities participated in focus groups and interviews - Caring Contacts was perceived in all communities to be acceptable - Feedback for intervention adaptions focused on the themes of trial eligibility criteria; instruments; message frequency, timing, and content; and cultural considerations |
| Kral, 2009 [97] | Review paper, Peer-Reviewed  Qualitative Narrative analysis | Canada | Inuit | N/A | Review: N/A | - According to Inuit traditional knowledge & practice, survival and thus suicide prevention involves hope, feeling loved and cared for, being able to talk about problems, having skills and taking responsibility for a better future and being proud of oneself. - Emphasis on patience, perseverance, love and caring in the family and community, communication, awareness of self and others, confidentiality and respect, and the taking of personal responsibility as important traditional values. - In relation to resilience, the Elders recommended tolerance and the understanding of others, helping others develop positive thinking, and that “parents must listen to and pay loving attention to children, showing they are cared for” (pg. 305). | - Details not provided | - Economic advancement is occurring in Inuit Nunaat following land claim settlements, and territorial and provincial governments are overseeing Inuit well-being. - Inuit community engagement in suicide prevention is taking place and studies are being planned to evaluate the efficacy of such action for suicide prevention and community mental health. - Initial evidence demonstrates that community control over suicide prevention itself can be effective towards preventing suicide. |
| LaFromboise, 1995 [96] | Primary research, Peer-reviewed  Mixed-method: self-report, behavioral  observation, & peer rating  n= varied by data collection method | USA | Zuni Tribe | Zuni Life Skills Development Program | Education/ awareness: A School/Community-Based Suicide  Prevention Intervention | “Suicide is an especially distressing phenomenon for the Zuni because suicide is forbidden in their traditional culture. Therefore, Zuni leaders initiated the development of a suicide prevention program in their high school with the purpose of reducing the factors associated with suicidal behavior.” (p. 479) | - Intervention developed in partnership with community (see LaFromboise, 2008 below) - Details not provided on engagement for development of evaluation | - Students exposed to the curriculum scored better than the no-intervention group at post-test on suicide probability and hopelessness. - The intervention group showed greater ability to perform problem-solving and suicide intervention skills in a behavioral assessment |
| LaFromboise, 2008 [56] | Descriptive paper, Peer-reviewed | USA | Zuni Tribe | Zuni Life Skills Development Program | Education/ awareness: A School/Community-Based Suicide  Prevention Intervention | - Intervention informed by a cultural hypothesis “that Zuni youth were engaged in fatal suicidal behaviour due to rising levels of hopelessness and decreased involvement in cultural traditions, which in turn was associated with increased family fragmentation and economic hardship.” (p. 351) - Authors also wanted to include a measure of family cohesion to test cultural hypothesis but it was deemed too personal for inclusion into study by stakeholders | - Community-based research - Community reached out to researchers to help with need to reduce youth suicide - Committee established of included members from Zuni Tribal council, Zuni board of education and Stanford University approved project. - On-going consultation with community leaders and members - Community took part in development and evaluation - Zuni professionals involved as cultural brokers between the students’ home and school contexts - Culturally tailored - Encouragement of use of Indigenous language - Each non-Zuni facilitator paired with a Zuni person to deliver the content | - Using a multi-method evaluation approach including self-report, behavioral observation, and peer rating, the intervention was found to reduce suicidal thoughts and behaviours and feelings of hopelessness among Zuni youth - Found to increase problem-solving skills and suicide intervention skills |
| LaFromboise, 2016 [80] | Book Chapter, Descriptive | USA | American Indian/Alaska Native | American Indian Life Skills (AILS) curriculum, also known as Zuni Life Skills Development Program | Education/ awareness: “school-based, culturally grounded, life-skills training program” | - Acknowledgement that "successful, the content of AI/AN suicide prevention interventions should be based first and foremost on comprehensive knowledge of culturally unique factors. Second, intervention developers should be responsive to the needs of the AI/AN community and actively engage its members in intervention design, evaluation, and implementation." (pg. 224) - Authors identify historical trauma, acculturation stress, and community violence on individual adaptation as unique risk factors for AI/AN youth - Enculturation was identified as a protective factor | - See above under Zuni Life Skills Development Program - Rather than being a one-size-fits-all intervention, the AILS encourages interventionists to incorporate traditional and contemporary worldviews of the tribes and communities they work with into the curriculum without compromising the core psychological components of the program or displacing the skills training outlined in the manual | - Program is promising for reducing depression and depressive symptoms and reducing suicidal thoughts and behaviors - Program is ineffective for improving self-concept |
| Lopes, 2012 [42] | Primary research, Peer-reviewed  Qualitative interviews  n=10 | Australia | Central Australian Indigenous communities | “Suicide Story” | Education/ awareness: Cultural adaption ASIST video training resource | Not discussed/defined | - Participatory action research approach - Indigenous people were involved in planning, implementing and dissemination of the program. - DVD resource was developed in collaboration with local Indigenous artists and film makers; Indigenous art was used throughout teaching materials and manuals. - Guided by principles of cultural safety; “culturally sensitive approach” (p. 226) - Cultural adaption | - DVD increased trainees’ knowledge and understanding about suicide particularly how to notice warning signs and how to help someone at risk of suicide and myths associated with Indigenous suicide - DVD was useful in initiating and encouraging conversation, providing trainees strength and empowerment and determination in acknowledging and dealing with these issues, and in providing a sense of hope - Trainees were supportive of future plans to deliver the training resource in other Indigenous communities. |
| May, 2005 [59] | Primary research, Peer-reviewed  Non-randomized study design  n=unclear | NM, USA | American Indian Tribal Nation (Western Athabaskan Tribal National of New Mexico) | The Adolescent Suicide Prevention Project | Community prevention: “Community wide systems suicide prevention model" p. 1239 | Not discussed/defined | - Project designed collaboratively by Indian Health Service (IHS) and Tribal officials. - Community-wide systems suicide prevention model development involved tribal leaders, Elders, youths, clients, and healthcare providers. - Community workshops held to answer three questions:   - What are the problems and issues in the community?   - What are the barriers to resolving these problems?   - What can be done to solve problems and overcome barriers? | - Both descriptive and linear regression analyses indicated that a substantial drop occurred in suicidal gestures and attempts. - Suicide deaths neither declined significantly nor increased, although the total number of self-destructive acts declined by 73% (P=.001). |
| Mehl-Madrona, 2020 [43] | Primary research, Peer reviewed  Qualitative narrative approach | North American Countries | North American Indigenous people | N/A | Culture as Treatment: Evaluation of Narrative Approach to Suicide Therapy | - "Culture matters. In Canada, communities with a strong sense of culture, language, and community ownership have lower rate of suicide than the Canadian mean and sometimes none at all." (pg. 1) - “Culturally relevant care ideally consists of advocacy, outreach, community-based interventions, and inclusion of indigenous psychotherapists.” (p. 1) - “Most of the psychotherapy is provided by nonindigenous practitioners and is not necessarily culture informed or proceeding from a social justice perspective. The methods and values of conventional mental health services may be incompatible with indigenous peoples, and the provision of services may further alienate and subjugate indigenous communities.” (p. 1) | - No community engagement processes appeared to take place - Authors hypothesized if a narrative approach to suicide psychotherapy would be culturally acceptable to Indigenous patients | - Nine major strategies within a narrative approach that appeared to be successful with this population identified - Introducing novel contradictory ideas to the beliefs people held about suicide appeared helpful - Using stories to introduce the idea that the desired effects of suicide might not be forthcoming seemed beneficial, including the use of story to find means other than attempting suicide to reach the same ends - Creating stories of a positive future appeared helpful - Finding ways to bring humor into the discussion and to refer to and involve traditional culture in which suicide was rare aided in changing perspective - Of 29 patients engaged in this narrative approach, 26 had no further suicide attempt - Retention in counseling was high, and patients reported enjoying the process |
| Middlebrook, 2001 [72] | Critical review, published in a peer-reviewed journal | AK, USA | American Indians & Alaska Natives | Numerous interventions | Review: Numerous interventions | Not discussed/defined | Details not provided | - Intervention methods included intergenerational events, cultural events, tribal courts, cultural practices, spiritual practices, and school-based curriculum with cultural relevance - Majority of programs identified in this review support two themes: the need for cultural relevance in all aspects of program development and implementation, and importance of community involvement - Local AI/AN communities are in a better position to understand the complexities of the problems that may affect them, and, as a result, they should ultimately create the solutions to these problems |
| Mohatt, 2014 [69] | Primary research, Peer-Reviewed  Pre/post survey design  n=114 | AK, USA | Yup'ik Native Youth | The People Awakening Program; Qungasvik | Community prevention: Strength-based, multi-level, community / culturally-grounded | - Intervention founded on an “Indigenous model of protection from alcohol use disorder that was also found to provide protection from suicide risk among AN youth - Feasibility assessment aimed to assess the extent to which (1) the intervention could be implemented in rural Alaska Native communities, and (2) the intervention was capable of producing measurable effects | - CBPR - Intervention created by community planning groups comprised of youth, parents, community leadership, community members, Elders, and university researchers - Cultural intervention | - In one community, medium dose response effects (d=.30–.50), with dose defined as number of intervention activities attended, were observed in the growth of intermediate protective factors and ultimate variables. - In the other community, medium dose effects were observed for one intermediate protective factor variable, and small dose effects were observed in ultimate variables. - Differences across communities in resources supporting intervention explain these contrasting outcomes. - Results suggest implementation in these settings is feasible when sufficient resources are available to sustain high levels of local commitment. In such cases, measurable effects are sufficient to warrant a prevention trial. |
| Muehlenkamp, 2009 [95] | Primary research, Peer-Reviewed  Pre/post study design  n=90 | USA | American Indian Students | N/A | Community prevention AND gatekeeper training AND education/ awareness: Cultural adaption medicine wheel suicide  prevention model | “[P]rogram is grounded within the AI cultural symbol of the medicine wheel….A key principle of the medicine wheel is interconnectedness, which emphasizes  that all aspects of one’s life influence the others. Thus, healing in one area can be impacted by healing in another. | - Details not provided on engagement for development of intervention - Program counselor is based in the American Indian Student Services Center - In order to address the spiritual needs of AI students on the University of North Dakota campus, a Spiritual Advisory Committee was formed to coordinate all spiritual ceremonies - Cultural adaptation | - Approximately 90 AI students (24.5% of total AI student enrollment; N=368) have utilized at least one aspect of the AI suicide prevention program - Of those having contact with the program, 4 were directly referred for crisis services, 36 attended program workshops or trainings, around 35 have requested or utilized ceremonial venues, and a number have had informal contact with program staff for support services, psychoeducation, or relationship building - Preliminary analyses, collected from 22 AI students who participated in gatekeeper trainings, indicate a high baseline suicide knowledge; Results from the post-test show significant, albeit small, improvement in knowledge. - Subjective reports from participants indicate that for 45.7%, the material presented was at least somewhat new to them, 72.5% stated they would definitely use the information, and 86.3% reported being very satisfied with the training. |
| Nasir, 2017 [82] | Primary research, Open Peer-Reviewed  Qualitative semi-structured interviews & focus groups  n=29 | Southern Queensland, Australia | Indigenous Australians | Indigenous Network Suicide Intervention Skills Training (INSIST) | Gatekeeper training | “Indigenous communities share elements of culture, language and heritage, and it is essential that suicide prevention and intervention programs encourage connectedness, facilitate assimilation, and maintain cultural heritage. Reports have shown that a community-led participatory approach is required to develop  a comprehensive, effective and long-term program that can tackle the multi-dimensional aspects of suicide  prevention and intervention” (p. 1) | - Indigenous community members consulted to evaluate content of existing suicide prevention gatekeeper programs for cultural appropriateness, especially Indigenous-specific interventions, and to define what would constitute a culturally-appropriate gatekeeper program | - Existing programs were time-intensive, expensive, unsustainable, included content irrelevant to Indigenous people, and were identified as burdensome. - Culturally inappropriate content, trainers did not know Indigenous culture, not relatable, lacked focus on social, emotional, and spiritual underpinnings of community wellbeing - Need for ready, practical, easy to use interventions, short duration, generalizable/adaptable across communities, relevant language and scenarios, inexpensive, empowering, sustainable, focus on social, emotional, and spiritual underpinnings of community wellbeing |
| O’Keefe, 2019 [94] | Primary research, Peer-reviewed  Randomized control trial  n=304 | USA | White Mountain Apache Tribe | New Hope and Elder's Resilience with Optimized Case Management | Community prevention: Resilience-based suicide prevention programs for AI youth | Authors indicate protection from suicide for AI/AN communities has been linked to cultural factors, including tribal spirituality, participation in cultural activities, social support from tribal leaders, and holistic connectedness to self/ family/ community/ land.   - New Hope video featuring AI actors portraying scenes specific to the characteristics of suicide attempts, ideation and related binge substance use among youth in community, with Elders speaking in Apache (with subtitles) about how life is sacred, how suicide is not the Apache way, how self-harm impacts the entire community, their concern for the youth, and the importance of each youth’s life - In Elder’s Resilience, each lesson is comprised of Elders introducing youth to Apache language, cultural knowledge, and stories, with a strong focus on respect and the sacredness of life. The curriculum features specific themes (e.g., respect, self-worth, spirituality, relationships/clan system) and relevant seasonal teachings | - Interventions adapted and/or co-created through strong tribal-academic partnership to target locally relevant modifiable risk and protective factors for suicide and related behaviors - Inclusion of culturally-grounded evaluation measures: involves subset of local White Mountain Apache Tribe-generated items relating to changes observed among youth - Collaborators also identified several items on proposed evaluation measures scales that did not seem culturally relevant, which were then excluded or re-worded | - Aim to evaluate:   - Primary outcomes: 1) suicide ideation as measured by the Suicide Ideation Questionnaire (SIQ/SIQ-JR), and 2) resilience as measured by a modified version of the Resiliency Scales for Children and Adolescents (RSEA)   - Secondary outcomes: depressive and anxiety symptoms, impulsivity, self-efficacy and communal mastery, importance of following AI values and cultural practices, self-esteem, hope, substance use, and a subset of local WMAT generated items relating to changes observed among youth who have previously received New Hope and/or Elders’ Resilience interventions |
| Philip, 2016 [93] | Primary research, published in a peer-reviewed journal  Pre/post study design  n=57 | AK, USA | Alaska Native Youth | Study of youth social networks’ predictive relationship to protective factors (Qungasvik model) from suicide and alcohol use disorder risk | Community prevention: Study of youth social networks in order to evaluate the extent to which network statistics are predictive of protective factors from suicide and alcohol use disorder risk | - Protective factors model (Qungasvik) is a Yup’ik community-level cultural model to increase protection against suicide and alcohol use disorder - Acknowledgement of influence of social factors in suicide and potential for social networks to play a role. | - Protective factors model (Qungasvik) emerged from CBPR processes - This study, however, does not detail community engagement processes | - Selected social network characteristics function as predictors of protective factors from suicide and alcohol use disorders among rural Yup’ik Alaska Native youth:   - Connections to Adults, associated with both family and community but not individual protective factors   - Connections to Elders and density if social network approached significance in relation to family protective factors   - minimal effects of social network on individual level protective factors - These findings provide empirical grounding for specific social network variables as targets for the Qungasvik intervention, constituting intermediate variables for prevention research and, potentially, are important elements in the process of change from intervention - Additional research on the role of social networks in improving Alaska Native and American Indian health and well-being is critically needed |
| Rasmus, 2014 [76] | Descriptive paper, Peer-reviewed | AK, USA | Yup'ik Alaska Native Youth | Qungasvik | Community prevention: Yup’ik Alaska Native approach to the prevention of suicide and alcohol abuse. | Suicide knowledge is defined by the Yup’ik Alaska Native community’s perspective. However, it is not explicitly stated. The community decided to take a strengths-based approach to suicide and alcohol abuse prevention. This is in alignment with Yup’ik perspectives which “focused more on understanding and addressing what is going on around the individual than addressing what is going on inside.” (p. 8) | - Program originally created by community; researchers subsequently invited to collaborate - CBPR - Research team consulted community’s joint resource group (consisting of local church, tribe, city, corporation, and school representatives; as well as Elders and natural helpers) - Community Planning Group and Working Group created to guide intervention and select Yup’ik settings promoting protective and prosocial activities within the community and culture respectively (groups consisted of Elders, community leaders, parents and youth) | - Modules each are representative of the process that the community co-researcher team took to develop and implement protective experiences that: (1) create supportive community, (2) strengthen families, and (3) give individuals tools to be healthy and strong |
| Rasmus, 2019 [53] | Descriptive paper, Peer-reviewed | AK, USA | Yup'ik Alaska Native Youth | The Qasgiq Model; Qungasvik | Community prevention: Yup’ik Alaska Native approach to the prevention of suicide and alcohol abuse. | The term ‘*qasgiq*’ means communal house, or can refer to a central place for community gatherings, ceremonies and celebrations. Derived from *qasgiqirayaq*. “*Qasgirarneq* (qaz gee raar neq) has a meaning to encircle. In coming together around our youth in the ways of our ancestors, we are strengthening our collective spirit in an effort to cast the spirit of suicide and substance abuse out from our communities, forever.” – Yup’ik Elder (p. 2) | - CBPR - Cultural interventions - Indigenous knowledge integrated with Western knowledge “when synergistic to the intervention goals” (p. 10) | - Recommended next steps for communities and researchers seeking mutual engagement in health prevention and promotion activities involve: a) advancing Indigenous Knowledge (IK) in community health interventions to reduce disparities and promote well-being; b) allowing IK to take lead in community health intervention research; c) focusing on community-level factors and cultural mechanisms in the development and evaluation of Indigenous interventions; d) developing measures and evaluation tools based in the IK; e) utilizing language, terms, symbols and theories from the culture and IK; f) identifying underlying functions of cultural mechanisms and process that may generalize across local contexts rather than rigid adherence to form as in strict components views of intervention ; and g) keeping in mind how ‘all communities have a qasgiq.’ |
| Rasmussen, 2018 [40] | Primary research, Peer-reviewed  Retrospective study design  n=335 | Australia | Aboriginal prisoners in Australia | No name provided for program | Community prevention: Aboriginal art program for prisoners | Not discussed/defined | - Art program facilitated by a cultural liaison officer - Cultural space provided Aboriginal prisoners with a social environment to practice Aboriginal art, socialise and make contact with visiting Elders - No specific description of how this program was designed to be culturally appropriate other than having cultural liaisons during the sessions (whose involvement or background is not discussed), and visits from Elders (details of which not indicated), no other efforts were taken to ensure cultural relevance. Seemed to be taking a more generalized pan-Indigenous approach. | - Of 335 Aboriginal prisoners, 108 (32.2%) attended the Aboriginal art program at least once and 227 did not. - Those who attended the Aboriginal art program were less likely to have a history of psychiatric illness (10.2% versus 19.8%), but more likely to have a history of violent offences (90.7% versus 67.4%) and more likely to have presented with grief/loss issues at receptions (24.1% versus 14.5%). - Both suicide/self-harm history and number of days attending Aboriginal art was associated with the incidence rate of suicide/self-harm risk assessments. - Controlling for a history of suicide self-harm, each day (and additional day) of attendance to the Aboriginal art program reduced the incidence rate of suicide/self-harm assessment by a factor of 0.81 (CI 95%: 0.70–0.95). |
| Ridani, 2015 [83] | Review paper, Peer-Reviewed | Australia | Aboriginal and Torres Strait Islander peoples | N/A | N/A: Review of primarily grey literature | “The emergence of suicide among  Aboriginal and Torres Strait Islander communities was not evidenced until 1980 and occurred amid a backdrop of major sociopolitical changes, including deinstitutionalization, the introduction of alcohol canteens, and the forcible removal of children. The Stolen Generation  resulted in interrupted parenting and  mentorship processes and displaced Aboriginal and Torres Strait Islander peoples from their culture and spirituality… the widespread intergenerational trauma, dispossession, and rapid social changes may have impacted child development environments and contributed to a collective loss of autonomy and cultural identity, thus  increasing the risk factors for suicide” (p. 112) | Details not provided | - Most programs targeted the whole community and were delivered through workshops, cultural activities, or creative outlets - Curriculums included suicide risk and protective factors, warning signs, and mental health. Many programs were poorly documented and evaluations did not include suicidal outcomes - Most evaluations considered process variables - Results from available outcome evaluations suggest that employing a whole of community approach and focusing on connectedness, belongingness and cultural heritage may be of benefit - Despite the challenges, there is a clear need to evaluate outcomes if prevention is to be progressed |
| Robinson, 2016 [98] | Descriptive paper, Peer-reviewed | North Australia, West Arnhem region | Indigenous students at Maningrida Community College in West Arnhem region of North Australia | N/A | Education/ awareness: Classroom-based suicide prevention program | Not discussed/defined | - Literature reviews, community consultations, and learning on emotional and social levels in Indigenous and non-Indigenous groups, were utilized to develop the program - Researchers, college staff, and regional youth services created the program’s curriculum | - The pilot program confirmed the need to adjust both pedagogical approach and curriculum content for the program to have resonance with students from this linguistic and cultural background and with varying levels of exposure to multiple stressors in disadvantaged community settings |
| Sareen, 2013 [37] | Primary research, Peer-reviewed  Randomized control trial  n=55 | Canada | First Nations on-reserve, northwestern Manitoba | ASIST; Resilience Retreat (RR) | Gatekeeper training/ Community prevention | Not discussed/defined | - Swampy Cree Community Advisory Committee was consulted with in regards to Resilience Retreat program creation, processes, content, appropriateness, recruitment, etc. | - In comparison with the Resilience Retreat, ASIST was not associated with a significant impact on all outcomes of the study based on intention-to-treat analysis. - There was a trend toward an increase in suicidal ideation among those who participated in the ASIST in comparison to those who were in the Resilience Retreat. - Lack of efficacy of ASIST in a First Nations on-reserve sample is concerning in the context of widespread policies in Canada on the use of gatekeeper training in suicide prevention. |
| Tighe, 2012 [57] | Primary research, Peer-reviewed | Australia | Indigenous youth (males) from the Kimberley region of northwestern Australia | Alive and Kicking! (AKG) | Education/ awareness: “sport-based suicide prevention  program” | “[F]ocus on risk factors negatively frames the lived experience of an Indigenous community by constructing suicide in terms of blame and powerlessness. Recent  suicide prevention strategies have subsequently focused on enhancing protective factors, such as community connectedness, personal capacity  and ownership of the programs.” (p. 240) | - Peer Educators (PE’s), Indigenous young people, determined what activities to facilitate, what to teach, and how to teach it. - PEs had full control of how suicide knowledge was being taught and disseminated. - However, external interventions such as ASIST were also used in programming | - Created a safe space for healing which allowed these young men to come to terms with the ‘cycle of grief ’ experienced in the region - Also tackled suicide stigma as the PEs, who were well-respected sportsmen, demonstrated that seeking help does not display weakness - At the conclusion of the pilot, 16 young men became PEs - They learned practical skills in suicide awareness and prevention which they can teach within and across the different communities within the Kimberley region |
| Tighe, 2017 [44] | Primary research, Peer-reviewed  Randomized controlled trial  n=61 | Australia | Indigenous Australian youth in remote communities | iBobbly mobile app | Culture as treatment: Self-help mobile app targeting suicidal ideation | - Informed by the social determinants of Indigenous health - “Indigenous suicide differs from non-Indigenous not least in terms of the different historical contexts, the broader sociocultural and sociopolitical issues, the triggers for suicidal behaviour, the suicidal behaviours themselves, and the conceptualisation of suicide within the context of social and emotional well-being.” (p.1) | - App co-developed with local Indigenous communities - Indigenous graphic designers and artists developed the imagery that accompanied activities and messages - Study examined the cultural relevance and effectiveness of app. | - Although preintervention and postintervention changes in depression (via the Depressive Symptom Inventory—Suicidality Subscale) were significant in the iBobbly arm (t=2.40; df=58.1; p=0.0195), these differences were not significant compared with the control arm (t=1.05; df=57.8; p=0.2962) - However, participants in the iBobbly group showed substantial and statistically significant reductions in distress (via Patient Health Questionnaire 9 and Kessler Psychological Distress Scale) - No differences were observed in impulsivity - Indigenous youth regarded the iBobbly app to be culturally safe and of therapeutic value |
| Tighe, 2020 [87] | Primary research, Peer-reviewed  Mixed-methods: survey & qualitative semi-structured interviews  n=13 | Australia | Aboriginal and Torres Strait Islander | iBobbly mobile app | Culture as treatment: Self-help mobile app targeting suicidal ideation | - The authors state, "Indigenous suicide and suicidal behavior are different from those of non-Indigenous Australians and influenced by poverty and a range of historical, sociocultural, and sociopolitical factors...the terms “mental health/illness” - Community members view mental health through their holistic interconnected model of Social and Emotional Wellbeing (SEWB). This collectivist understanding of health care takes a community-wide view and values connection to family, culture, ancestry, land, and spirituality as some of the factors in maintaining wellness." (pg. 1) | - App co-developed with local Indigenous communities - Indigenous graphic designers and artists developed the imagery that accompanied activities and messages - Study evaluated app’s impact on psychological outcomes via psychological scales and thematic analysis of semi-structured interviews - Also examined the cultural relevance and effectiveness of app. | - Regression analysis indicated that app use improved psychological outcomes (suicidal ideation and distress), although only minimally, and effects were not significant - Results of the thematic analysis indicated that the iBobbly app was deemed effective, acceptable, and culturally appropriate |
| Tingey, 2016 [58] | Primary research, Peer-reviewed  Randomized control trial  n=199 | USA | White Mountain Apache Community youth | Arrowhead Business Group-Apache Youth Entrepreneurship Program (ABG) | Education/ awareness: “youth entrepreneurship education model to promote culturally based protective factors for substance use and suicide prevention” | - Informed by a positive youth development framework which posits that “youth who develop mastery and are supported by caring adults and peers to cultivate new skills are more likely to exercise control over their lives by making healthy choices and withstanding external pressures.” (p. 251) - Discussion on how predominating prevention models focus primarily on risk and utilize deficit-based approaches. The fields of substance use and suicide prevention research urge for positive youth development frameworks that are strength based and target change at individual and community levels | - Program and the evaluation plan were created in partnership between the White Mountain Apache Tribe (Apache) and the John Hopkins Center for American Indian Health (JHU). - A Community Advisory Board (CAB) formulated to explore areas for the development of interventions; comprised of youth and adults from the Apache community. - CAB in collaboration with researchers developed goals of the program using a positive youth development framework - Life skills curriculum content was adopted from pre-existing curriculum and activities that were created in partnership between the tribal community and academic partners. - Apache community members were facilitators of the intervention. and led the activities for the control group | - Only outcomes of process development discussed - Entrepreneurship education is a promising model of positive youth development to promote life skills and school connectedness and to reduce high risk behaviors in AI communities - Evaluation of such a program is a highly innovative approach to substance use and suicide prevention with potential for replication in other Indigenous and similarly stressed communities - If study aims are achieved, the field will have a new strength-based strategy to reduce adolescent substance use and suicide, the largest health disparities faced by AI communities |
| Trout, 2018 [55] | Primary research, Peer-reviewed  Qualitative via learning circles  n=unclear | USA | Alaskan Native | Evaluating feasibility and acceptability outcomes of Promoting Community Conversations About Research to End Suicide (PC CARES) | Education/ awareness: Evaluation of “Community of Practice to Prevent Suicide Through Multiple Channels” | - "[M]any Indigenous people point out that suicide is not readily circumscribed by medical understandings and strategies for intervention. - Acknowledgement of social inequity, historical trauma, and rapid and imposed social change as drivers of suicide risk that warrant a nonmedical, tribally- governed, and community-driven response - "Participating communities promoted the idea that Indigenous youth suicide is not just a matter of distressed individuals, but of a complex history reflected in the fundamentally social act of suicide and that effective interventions require community-level change." (pg. 403) - “[P]articipating communities promoted the idea that indigenous youth suicide is not just a matter of distressed individuals, but of a complex history reflected in the fundamentally social act of suicide—and that effective interventions require community-level change.” (p. 11) | - For details specific to development and implementation of PC CARES, see Wexler, 2016 - Details of how the evaluation was developed and whether community was engaged (including on selection of outcome measures) was not provided | - First, PC CARES as process emphasizes decoloniality by bringing community members together to acknowledge their collective strength and abilities. - Second, PC CARES as pedagogy supports a decolonial process by inviting participants to use their own knowledge to determine if and how the research evidence relates to their experiences and community - Third, PC CARES as practice emphasizes decoloniality by inviting community-based action. - The approach facilitates dialogue and planning for suicide prevention by utilizing research evidence, while also supporting local people in articulating their own ideas for personal and collective suicide prevention. - Results support our hypothesis that this educational model is both feasible and acceptable as a framework for incorporating both research evidence and Indigenous ways of knowing. - PC CARES builds the capacity, confidence, and collective commitment to self-determined suicide prevention practice among Alaska Native people. |
| Wexler, 2015 [91] | Descriptive paper, Peer-reviewed | USA | Alaska Native Youth | Collaborations for At-Risk (youth) Engagement and Support (CARES) | Community prevention: “Engaged storytelling approach to suicide prevention” | - “Suicide is an act with many meanings” - Youth suicide does not carry a single meaning, nor is it a stable, certain or ‘tame’ problem. As such, it cannot be solved or contained, through an exclusive reliance on predetermined, standardized, decontextualized interventions’ - Recognizes that many of biggest threats to well being among Indigenous youth arise from existing structural inequities that cannot be addressed through the provision of mental health services - Thus, any approach to suicide prevention or healing needs to take account of the enduring negative effects of colonization and the unique role of historical trauma in the lives of Indigenous youth, families, and communities. | - CARES created in collaboration with Indigenous community members and leaders - Facilitated by local individuals - Suicide prevention research formulates elements of the program, but majority of the content is driven by discussions among local community members (including their own experiences and stories). - Utilizes local knowledge through its storytelling approach. - Local facilitators encourage community discussions that seek to generate ideas and enhance readiness to respond to a person in need - Wisdom and knowledge generated on how to respond to suicidality, or those in need, is generated by the community and for the community | - Engaged and storied approach of CARES respects the role of history, tradition, cultural protocols, stories, and local community norms as important resources to include in any educational endeavor, including gatekeeper training. - Instead of relying on referrals to formal mental health services as the primary goal of gatekeeper training, authors recommend placing more emphasis on the important role that stories and communities play in providing stability, hope, cultural connectedness, and a sense of belonging for young people |
| Wexler, 2016 [54] | Descriptive paper, Peer-reviewed | USA | Alaska Native | Promoting Community Conversations About Research to End Suicide (PC CARES) | Education/ awareness: “Community of Practice to Prevent Suicide Through Multiple Channels” | - “[S]uicide does not carry a single meaning, nor is it a stable, certain or ‘tame’ problem….it cannot be solved or contained, through an exclusive reliance on predetermined, standardized, decontextualized interventions” (p. 5) - “Basically, suicide—as a ‘soul wound’—requires a ‘postcolonial form of therapeutic intervention.’ This kind of intervention must acknowledge local wisdom and practices and rely on indigenous ways of knowing and doing. As such, it is important to reflect relational, familial, social, and spiritual dimensions of selfhood more than decontextualized, expert-driven, individualistic, biomedical understandings of distress.” - Authors acknowledge impacts of forced social change, colonization, has led to intergenerational trauma, and the social, economic, and political inequalities experienced by these communities create conditions that increase suicide risk and can reduce people’s access to shared protective factors and processes. - “Imperative that suicide prevention includes decolonization…while also utilizing the “best practices” from research to effectively address the issue from multiple levels.” (p. 2) | - Intervention developed with indigenous leaders and service providers from rural Alaska; details on development process not provided. - Delivered by “mostly Indigenous” facilitators - Uses a “community of practice” model that invites community stakeholders, tribal leaders, rural providers of health and human services, law enforcement, religious heads, and others to come together each month to learn “what we know, think, and want to do” about suicide and suicide prevention - Intervention offers way to address locally identified gaps in understanding and collaboration through (a) engaging key community members in ongoing learning about suicide prevention based on scientific research, (b) applying this knowledge to their villages and lives, and (c) supporting a broad range of actions to prevent suicide and promote wellness, on participants’ own terms. | - Pilot sessions, conducted in six village communities suggest that these aims are supported by the PC CARES approach - Further evaluation study is pending |
| Wexler, 2017 [92] | Primary research, peer-reviewed  Pre/post study design  n=32 | AK, USA | Alaska Native Youth | Evaluating learning and behavioural outcomes of Promoting Community Conversations About Research to End Suicide (PC CARES) | Education/ awareness: Evaluation of “Community of Practice to Prevent Suicide Through Multiple Channels” | See above | - For details specific to development and implementation of PC CARES, see Wexler, 2016 - All evaluation research was approved by the institutional review board of the [host institution], and conducted in partnership with the tribal health and social services organisation serving the region - Details of this partnership, how the evaluation was developed and whether community was engaged (including on selection of outcome measures) was not provided - Research team brought active local, Indigenous Facilitators came together twice to reflect on the feasibility and value of the model, to identify and address areas for improvement, reflect on the whole intervention, and provide ideas for increasing successful implementation in the future | - 20 of the 32 trained facilitators in 10 of the 11 participating villages have hosted 54 LCs, with a total of 309 unique community members - Coding of these learning circles by 2 independent raters indicate acceptable levels of fidelity and accurate dissemination of research evidence by facilitators - Facilitator reflections were positive overall, suggesting PC CARES is feasible, acceptable and potentially impactful as a way to translate research to practice in under-resourced, rural AN communities - PC CARES represents a practical community education and mobilisation approach to Indigenous youth suicide prevention that displays preliminary success in learning and behavioural outcomes of local facilitators |
| Wexler, 2019 [123] | Primary research, Peer-reviewed  Mixed-methods: pre/post surveys, learning circles, & non-randomized control trial  n=varied by data collection method | USA | Inuit and Alaska Native | Evaluation of process and preliminary outcomes of Promoting Community Conversations About Research to End Suicide (PC CARES) | Education/ awareness: Evaluation of process and preliminary outcomes of Promoting Community Conversations About Research to End Suicide (PC CARES) | "[F]orced settlement and mandatory schooling, including residential schools, changed family structures, and young people's experiences of growing up change people in significant ways. These forms of colonization and intergenerational trauma can create conditions that increase suicide risk and can reduce people's access to shared protective factors and processes. Considering this, suicide prevention must prioritize cultural and local knowledge, respond to local sensibilities and conditions, while also utilizing scientific prevention research to effectively address the issue." (pg. 399) | - For details specific to development and implementation of PC CARES, see Wexler, 2016 - Details of how the evaluation was developed and whether community was engaged (including on selection of outcome measures) was not provided | - Local facilitators achieved acceptable fidelity to the model (80%), and interpreted the research accurately 81% of the time. - Discussions reflected participants’ understanding of the research content and its use in their lives. - Participants showed positive changes in perceived knowledge, skills, and attitudes and strengthened their ‘community of practice’ from baseline to follow-up. - Social network analyses indicate social impact, sustaining and enhancing prevention activities of non-participants who were ‘close to’ participants. These close associates were more likely take preventive actions than other non-participants after the intervention. - PC CARES offers a practical, scalable method for community-based translation of research evidence into self-determined, culturally-responsive suicide prevention practice. |

^1^CBPR: Community-based participatory research (CBPR) is identified in articles that explicitly referenced using this approach to community engagement. CBPR is an approach which researchers and community stakeholders engage as equal partners in all steps of the research process with special attention to power relationships that are inherently embedded in Western knowledge production. The approach advocates for power to be shared between the researcher and the researched, acknowledges the legitimacy of experiential knowledge, and focuses on research aimed at bringing about social change [124].

^2^Cultural intervention refers to those interventions identified in the literature in which Indigenous culture is both a central focus of the intervention activities and underlies the theory guiding the intervention. This contrasts interventions that were identified as “cultural adaptions” or “culturally-appropriate, -sensitive, -tailored, or -safe,” which may have incorporated Indigenous cultural activities, teachings, and/or language but were still primarily rooted in conventional Western/colonial understandings of suicide and mental health.
