# Supplementary material for: Centering Indigenous knowledge in suicide prevention: a critical scoping review
Source: BMC Public Health. 2022 Dec 19;22:2377. doi: 10.1186/s12889-022-14580-0 (PMC9761945; doi:10.1186/s12889-022-14580-0)
Supplement: Supplementary file 3 — Additional file 3: Appendix C. Summary of grey literature articles [125–130]. [file 12889_2022_14580_MOESM3_ESM.docx]

**APPENDIX C: Summary of grey literature articles**

| Source | Article Name | Country/ Region | Indigenous population | Type of Resource/ Description | Type of Publication |
| --- | --- | --- | --- | --- | --- |
| Lifeline, 2005 [125] | Aboriginal Suicide Prevention Information | Australia | Illawarra/Shoalhaven Aboriginal communities | Suicide Intervention Toolkit | Brochure |
| Paul, 1988 [63] | Early detection key to suicide prevention | North America | Native people | Interview with North American researchers about suicide prevention and early detection | News Article |
| Ontario Secondary School Teachers’ Federation, 2012 [126] | Full Circle: First Nations, Métis, Inuit Ways of Knowing A Common Threads Resource | Ontario, Canada | First Nations, Métis or Inuit | Curricular materials that focus on First Nations, Métis and Inuit people, history and culture. | Curriculum document |
| Alberta Health Services, 2009 [75] | Honouring Life Aboriginal Youth and Communities Empowerment Strategy | Alberta, Canada | Indigenous youth in Alberta | Aboriginal Youth  Suicide Prevention Strategy | Regional health promotion strategy |
| Ajunnginiq Centre of the National Aboriginal Health Organization, 2006 [127] | Suicide Prevention: Inuit Traditional Practices That Encouraged Resilience and Coping | Canada | Inuit | Outcomes of a research project to gather some of the beliefs and strategies that were used by Inuit to promote and preserve life | Research /community engagement outcomes report |
| McCormack, Mohammed, O’Brien, 2001 [100] | Learning to Work with the Community: The Development of the Wujal Guidelines for Supporting People Who are At Risk | Australia | Wujal Wujal community | Process review for development of a suicide prevention project | Primary research, Peer-reviewed |
| Daniels, 2001 [99] | Bubu Dayirr Mal ("The Sunrise") | Australia | Wujal Wujal community | Description of the Mass Media Tools Project to address suicide prevention and life promotion | Journal article, Peer-reviewed |
| Small and Big Horn Jr, n.d. [128] | Native H.O.P.E. Helping Our People Endure | USA | American Indian/Alaska Native youth | Suicide education and awareness curriculum | Curriculum document |
| Durand, 2012 [129] | Ondinnok: the First  Nations Theatre of  Quebec | Canada | First Nations | Introduction/overview of First Nations theatre in Quebec. Author calls theatre grounded in First Nations mythology, not merely as an act of reconstitution but as an act of “cultural reconstruction” in the face of continuous dispossession & assimilation | Magazine article |
| Henderson, 2017 [65] | Of the Heart Scoping Review of Indigenous Youth Suicide and Prevention | International (Canadian focus) | Indigenous peoples | Scoping review that explores Canadian and international peer-reviewed literature and Canadian grey literature addressing Indigenous youth suicide and resilience | Scoping review, not peer-reviewed |
| Inungni Sapujjijiit Task Force on Suicide Prevention and Community Healing, 2003 [66] | Our Words Must Come Back To Us: Report of the Inungni Sapujjijiit Task Force on Suicide Prevention and Community Healing | Canada | Inuit (Nunavummiut) in Territory of Nunavut | Community engagement outcomes to understand suicide among the Nunavummiut and gather recommendations to address the issue | Government report (Nunavut) |
| Gooda, 2014 [64] | The Elders’ Report  Into Preventing Indigenous Self-harm  & Youth Suicide | Australia | Indigenous Australians | Community engagement outcomes with Elders and community representatives on how to address the youth suicide and self-harm crisis | Non-governmental organization report (People Culture  Environment & Our Generation  Media) |
| U.S. Department of Health and Human Services, 2010 [71] | To Live To See the Great Day That Dawns: Preventing Suicide by American Indian and Alaska Native Youth and Young Adults | USA | American Indian and Alaska Native Youth | Guide is to support AI/AN  communities and those who serve them in  developing effective, culturally appropriate  suicide prevention plans. | Government report (U.S. Department of Health and Human Services) |
| Kelly, 2007 [130] | Traditional and Contemporary Approaches to Youth Suicide Prevention | Canada | First Nations | Guide for the National Youth Council: compilation of research covering best practices of suicide prevention | First Nations government report (National Youth Council, Assembly of First Nations) |
| Assembly of First Nations, n.d. [75] | Working Together to Address Suicide in First Nations Communities: A select illustration of collaborative, community-based, suicide prevention projects | Canada | First Nations | Resource sharing  stories of prevention and resiliency: highlights examples of thriving collaborative, community-based, suicide prevention projects that are funded through the National  Aboriginal Youth Suicide Prevention Strategy | First Nations government report (Assembly of First Nations) |
| First Nations Inuit Health Branch, 2006 [77] | What is Working, What is Hopeful: Supporting Suicide Prevention Strategies Within Indigenous Communities (and any other community that’s interested) | - Canada | - Indigenous communities in Canada | - Guide on how to collect and tell community stories as they relate to suicide - Stories are about how suicide impacted the community, those situations and events that contributed to suicidal behavior, and how the community recovered, is recovering, or will recover | Government / health services report (First Nations and Inuit Health Branch) |
